# Ultrasound-assisted synthesis of kojic acid-1,2,3-triazole based dihydropyrano[3,2-*b*]pyran derivatives using Fe_3_O_4_@CQD@CuI as a novel nanomagnetic catalyst

**DOI:** 10.1038/s41598-022-24089-6

**Published:** 2022-11-19

**Authors:** Zahra Najafi, Soheila Esmaili, Behnam Khaleseh, Saeed Babaee, Mehdi Khoshneviszadeh, Gholamabbas Chehardoli, Tahmineh Akbarzadeh

**Affiliations:** 1 Department of Medicinal Chemistry, School of Pharmacy, Hamadan University of Medical Sciences, Hamadan, Iran; 2 Department of Organic Chemistry, Faculty of Chemistry, Bu-Ali Sina University, Hamedan, Iran; 3 Department of Medicinal Chemistry, School of Pharmacy, Shiraz University of Medical Sciences, Shiraz, Iran; 4 Department of Medicinal Chemistry, Faculty of Pharmacy, Tehran University of Medical Sciences, Tehran, Iran

**Keywords:** Chemistry, Nanoscience and technology

## Abstract

The magnetic nanoparticles coated with carbon quantum dot and copper (I) iodide (Fe_3_O_4_@CQD@CuI) were used as eco-friendly heterogeneous Lewis / Brønsted acid sites and Cu (I) nanocatalysts. In the first step, it was applied in the synthesis of kojic acid-based dihydropyrano[3,2-*b*]pyran derivatives in a three-component reaction and in the second step, as a recyclable catalyst for the synthesis of kojic acid-1,2,3-triazole based dihydropyrano[3,2-*b*]pyran derivatives in the CuI-catalyzed azide/alkyne cycloaddition (CuAAC) reaction. The catalyst was characterized fully by using the different techniques including fourier transform infrared spectroscopy (FT-IR), elemental mapping analysis, X-ray photoelectron spectroscopy (XPS), scanning electron microscopy (SEM), X-ray spectroscopy (EDX), transmission electron microscopy (TEM), thermal gravimetric (TG) and value-stream mapping (VSM) methods. The final synthesized derivatives were identified by ^1^H- and ^13^C-NMR spectroscopy.

## Introduction

Carbon quantum dots (CQDs) are the latest class and one of the usage nanoparticles including carbon and heteroatoms in their structure. The CQDs because of the three-dimensional truncation have more atoms on their surfaces^1,2^. These materials have a parallel arrangement of carbons with a large number of carboxylic acid groups on their surface that caused to be the good solubility in aqueous media. This type of structure plays a major role for CQDs in various applications such as catalyst^3,4^, biotechnology^5,6^, sensors^7^, and chemiluminescence^8^, waste water^9^ and food safety^10^. CQDs have a wide variety of functional groups on their surface used as catalysts and substrates are used in the preparation of various catalysts^3,4,11–14^.

Performing a chemical reaction under ultrasound condition can be explained by a physical phenomenon called cavitation: cavitation is a phenomenon in which a decrease in pressure causes the liquid to evaporate locally and bubbles to form^15,16^. The bursting of bubbles produces a shock wave with enough energy to break the covalent bond. Sonication can be used to speed dissolution, by breaking intermolecular interactions^17^. Ultrasonic is used in the synthesis of various biological, pharmaceutical, and chemical compounds in mild or green conditions^15,18,19^. Ultrasonic provides the possibility of performing various chemical reactions such as coupling^20^, compaction, nitration^21^, and click^22^ in milder conditions, higher efficiency and green and environmentally friendly solvents.

Heterocyclic compounds are a group of organic chemical compounds in which some or all of the atoms of its molecules in the ring consist of an atom of an element other than carbon (C)^23^. The emergence of heteroatoms in the skeleton of chemical compounds is a reason for the emergence of various biological properties that can change the applications of chemical compounds and be used as drugs, pesticides, and solar cells^24–32^. The heteroatomic polycyclic compounds exhibit broad biological properties compared to simple mono-cyclic compounds^33–37^. The presence of each ring in the skeleton is a reason for the occurrence of biological and medicinal properties in the structure^38,39^. In 2001, Club, Finn, and Sharpless introduced click reaction as a group of chemical reactions in the synthesis of heterocycles that have potential advantages over traditional reactions such as ease of execution, easy separation, and inexpensive solvents. The most used “click” reaction that can fulfil these conditions is by far the CuI-catalyzed azide/alkyne cycloaddition (CuAAC)^40–48^.

**Figure 1:**
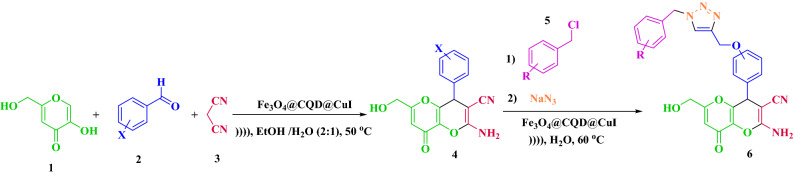
Synthesis of kojic acid based dihydropyrano-pyran and kojic acid-triazole based dihydropyrano-pyran in click reaction by Fe_3_O_4_@CQD@CuI as catalyst in the optimized conditions.

In this paper, we successfully developed a new method for the synthesis of heterocyclic polycyclic compounds using a new heterogeneous nanocatalyst based on CQDs as a nano-catalyst under ultrasonic condition. The novel catalyst Fe_3_O_4_@CQD@CuI was used to synthesize kojic acid-based dihydropyrano[3,2-*b*]pyran derivatives in a multicomponent reaction of kojic acid, malononitrile, and various aldehydes and kojic acid-triazole based dihydropyrano-pyran derivatives via a click reaction, respectively. Subsequently, the newer triazole compounds were synthesized using benzyl halide derivatives and sodium azide (Fig. 1).

## Experimental

### General

Transmission electron microscopy (TEM) was determined by TEM Philips EM 208S. X-ray photoelectron spectroscopy (XPS) was recorded by BESTEC (EA 10). Vibrating sample magnetometer (VSM) was created by LBKFB model Meghnatis Daghigh Kavir Company. Scanning electron microscope (SEM) was produced by FE-SEM ZEISS Sigma 300. Energy dispersive X-ray (EDX) was performed by Fesem Tescan Mira 2.

#### Synthesis of Fe_3_O_4_ nanoparticles

**Figure 2:**
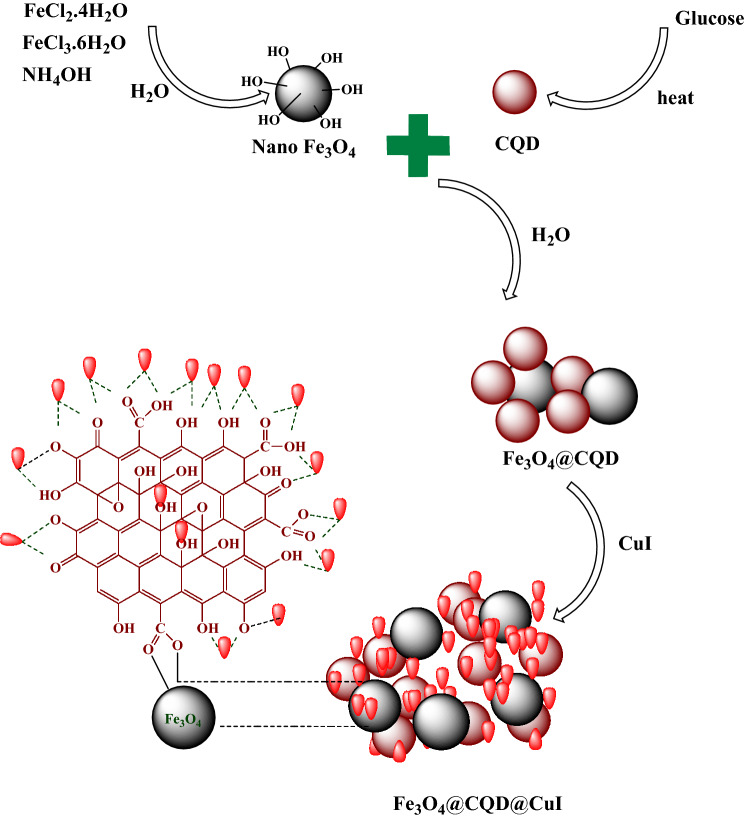
Schematic diagram of Fe_3_O_4_@CQD@CuI synthesis^3^.

In a 250 mL round-bottomed flask, 10 mmol FeCl_3_·6H_2_O and 5 mmol FeCl_2_·4H_2_O were well dissolved in 100 mL distilled water and was stirred. Then 10 mL NH_4_OH drop by drop was added to the mixture until the pH reached to 11. Then, the mixture was stirred under reflux condition for 1 h under N_2_ atmosphere. Finally, iron oxide nanoparticles were separated with an external magnet and washed several times with distilled water (Fig. 2)^49^.

#### Synthesis of CQD with glucose

In a 100 mL round-bottomed flask, 5 g of glucose was added to 10 cc of oil, a mixture of oleic acid (65%), linoleic acid (30%) and stearic acid (5%), which had already been heated to 250 °C. Half-burning of glucose in a mixture of the above acids led to the formation of carbon quantum dot. With the browning of glucose, the burning of glucose stopped and after cooling the mixture, by adding 30 mL of water and 30 mL of diethyl ether, carbon dot was separated through the aqueous phase (Fig. 2)^13^.

#### Synthesis of Fe_3_O_4_@CQD nanocomposite

Iron oxide nanoparticles Fe_3_O_4_ (1 g) was dispersed in 50 mL of water for 15 min with ultrasonic, then 0.05 g of carbon dot was added and stirred well for 24 h at room temperature. Finally, it was easily separated by an external magnetic field and washed twice with distilled water (Fig. 2)^14^.

#### Synthesis of Fe_3_O_4_@CQD@CuI

Loading of copper iodide on the Fe_3_O_4_@CQD was done by dispersing 1 g of Fe_3_O_4_@CQD in 50 mL of methanol, on the other hand 1 mmol of copper iodide was sonicated in 5 mL of methanol and then two solutions were mixed and stirred for 12 h under reflux condition. Finally, it was separated with a super magnet and washed with methanol (3 × 5) (Fig. 2).

### General procedure for the synthesis of kojic acid based dihydropyrano[3,2-b]pyran-derivatives using Fe_3_O_4_@CQD@CuI

A mixture of kojic acid (5-hydroxy-2-(hydroxymethyl)-4*H*-pyran-4-one) (1 mmol, 0.142 g), aromatic aldehydes (1 mmol), malononitrile (1.2 mmol, 0.066 g) and nano catalyst Fe_3_O_4_@CQD@CuI (5 mg) in a round-bottomed flask were sonicated in an ultrasonic bath in mixture of ethanol and H_2_O (2:1) as solvent. The progress of the reaction was monitored using TLC (*n*-hexane:ethyl acetate, 1:3). After the reaction was completed, the insoluble catalyst was easily separated by an external magnet bar. After evaporation of solvent, the precipitate was collected and recrystallized with ethanol (5 mL) to afford the pure product (Fig. 1). The analytic results (melting points, FT-IR, NMR) are shown in the supporting file (Supplementary Information).

### General procedure for the synthesis of kojic acid-triazole based dihydropyrano-pyran derivatives using Fe_3_O_4_@CQD@CuI

In a 25 mL round-bottomed flask, A mixture of kojic acid based dihydropyrano[3,2-b]pyran-derivatives (4-hydroxy, 3hydroxy and vanillin) (1 mmol), sodium azide (1.2 mmol, 0.078 g), benzyl chloride derivatives (1.2 mmol) and nano catalyst Fe_3_O_4_@CQD@CuI (0.01 g) were sonicated in 5 mL water. The progress of the reaction was monitored using TLC (ethyl acetate:MeOH, 8:1). After the reaction was completed, 5 mL ethyl acetate was added and the catalyst was easily separated by an external magnet bar. Then, the product was separated through the organic phase. The residue was purified by plate chromatography (ethyl acetate: methanol, 95:5) to give the desired products (Fig. 1). The analytic results (melting points, FT-IR, NMR) are provided in the supporting file (Supplementary Information).

## Result and discussion

The structure of Fe_3_O_4_@CQD@CuI as a nano magnetic catalyst coated with carbon quantum dot containing the hydroxyl and carboxyl groups on its surface with copper iodide, was studied and fully characterized by FT-IR, elemental mapping analysis, the scanning electron microscopy (SEM), X-ray spectroscopy (EDX), transmission electron microscopy (TEM), Thermal gravimetric (TG-DTG), X-ray photoelectron spectroscopy (XPS) and value-stream mapping (VSM) methods.

**Figure 3:**
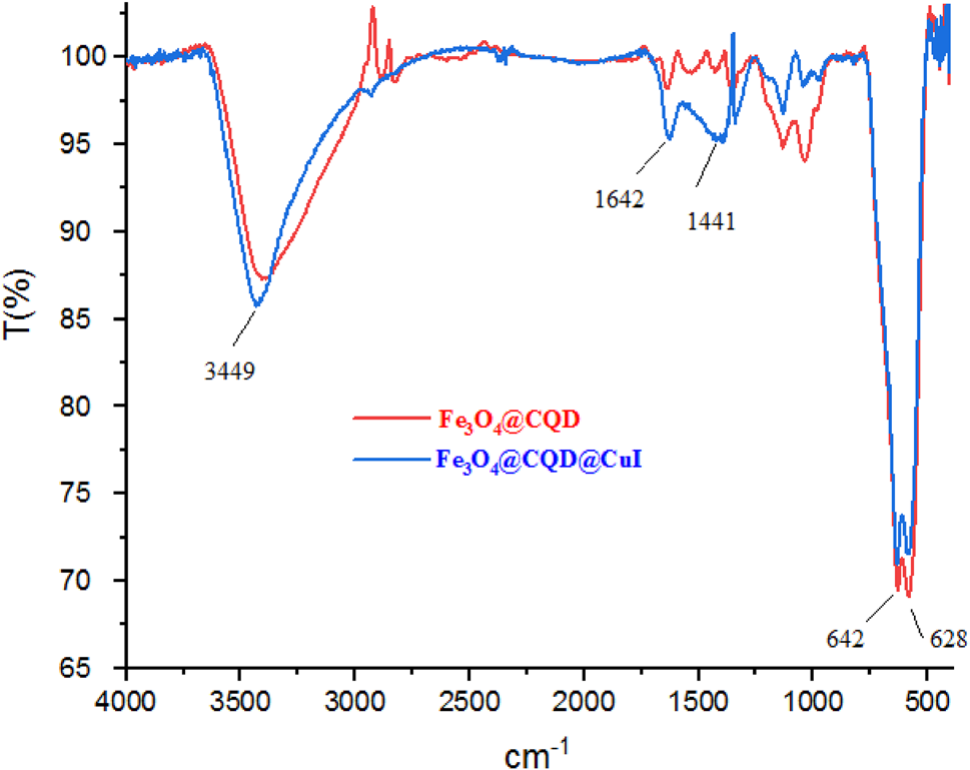
FT-IR spectra of Fe_3_O_4_@CQD and Fe_3_O_4_@CQD@CuI in KBr.

The characterization of Fe_3_O_4_@CQD and Fe_3_O_4_@CQD@CuI were confirmed and compared by FT-IR spectroscopy in Fig. 3. The broad peak that appeared at 3000–3500 cm^−1^ was related to OH and CO_2_H groups of CQD. Also, the absorption bands appeared at 1642 cm^−1^ and 1441 cm^−1^ Which are related to stretching modes C=O and C=C bonds, respectively. The peak in the region of 1000 to 1200 cm^−1^ is related to the C–O stretching modes of CQD. The aromatic ring in the carbon dot skeleton was observed. Furthermore, the peak of Fe–O of Fe_3_O_4_ appeared at 642 cm^−1^.

**Figure 4:**
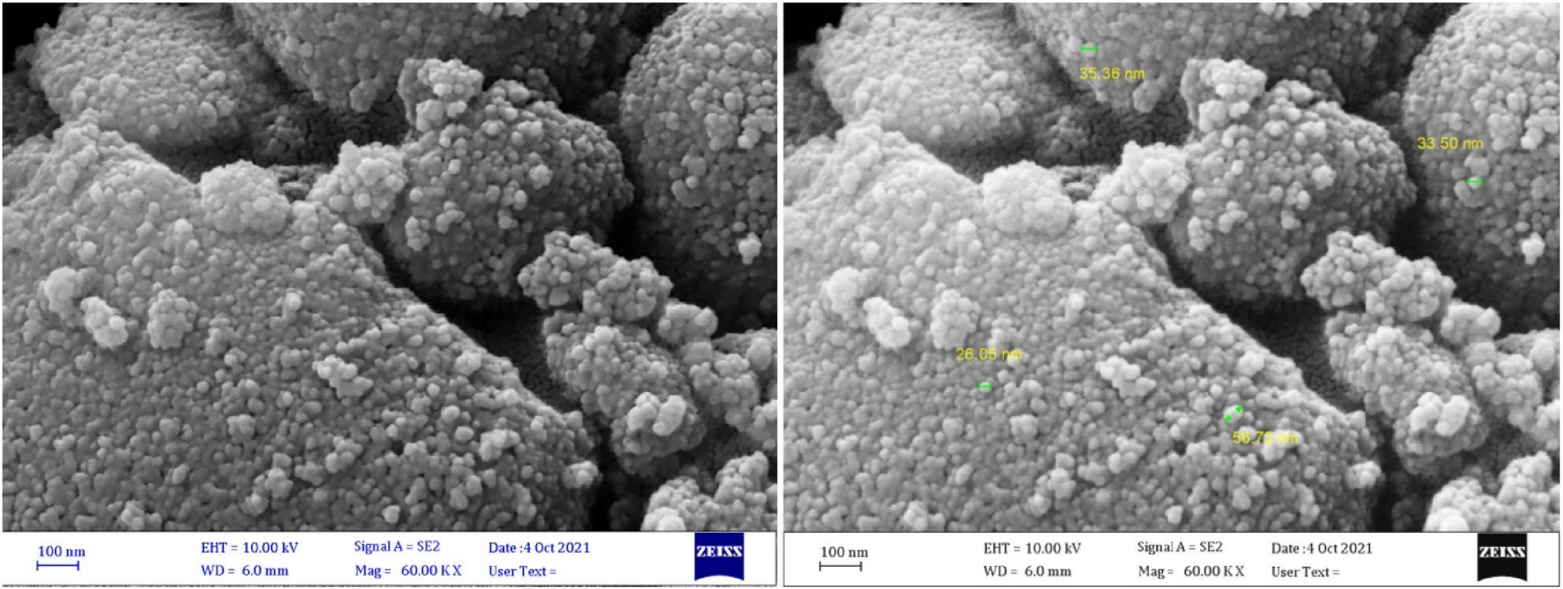
SEM images of Fe_3_O_4_@CQD@CuI.

**Figure 5:**
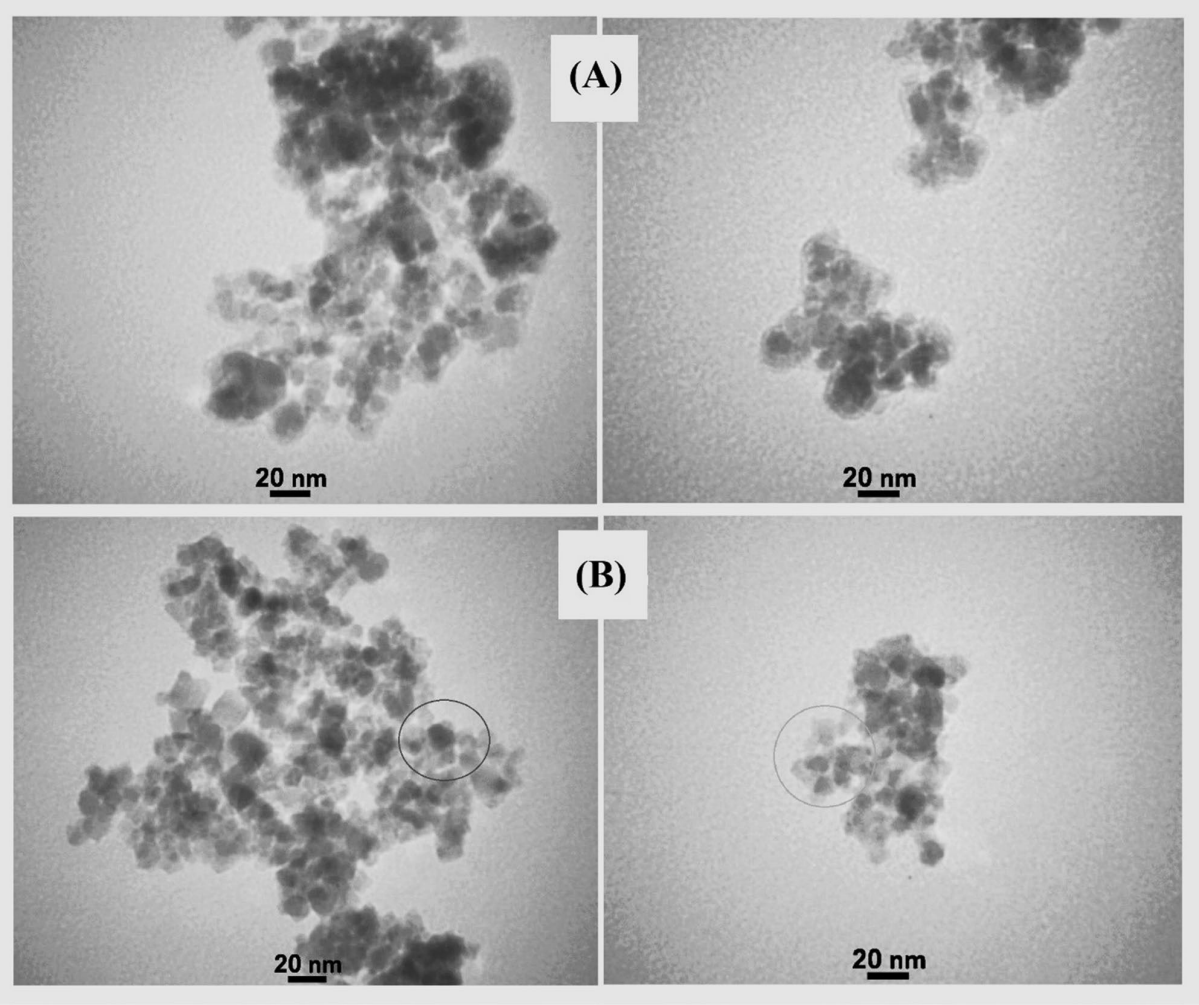
TEM images of (**A**) Fe_3_O_4_@CQD and (**B**) Fe_3_O_4_@CQD@CuI.

Using SEM, the morphology of the surface and particle size of Fe_3_O_4_@CQD@CuI were investigated. In Fig. 4, SEM images revealed that the shape of particles was spherical and dimensions were in a nanoscale size (approximately 26–55 nm based on images of SEM). TEM images (Fig. 5A,B) showed that the morphology of Fe_3_O_4_ nanoparticles was also spherical and the average size was less than 20 nm. TEM images also indicated numerous small particles (CQDs) with sizes about fewer than 10 nm surrounding Fe_3_O_4_ nanoparticle, evidencing that CQDs were successfully synthesized on the Fe_3_O_4_ nanoparticles^11^.

**Figure 6:**
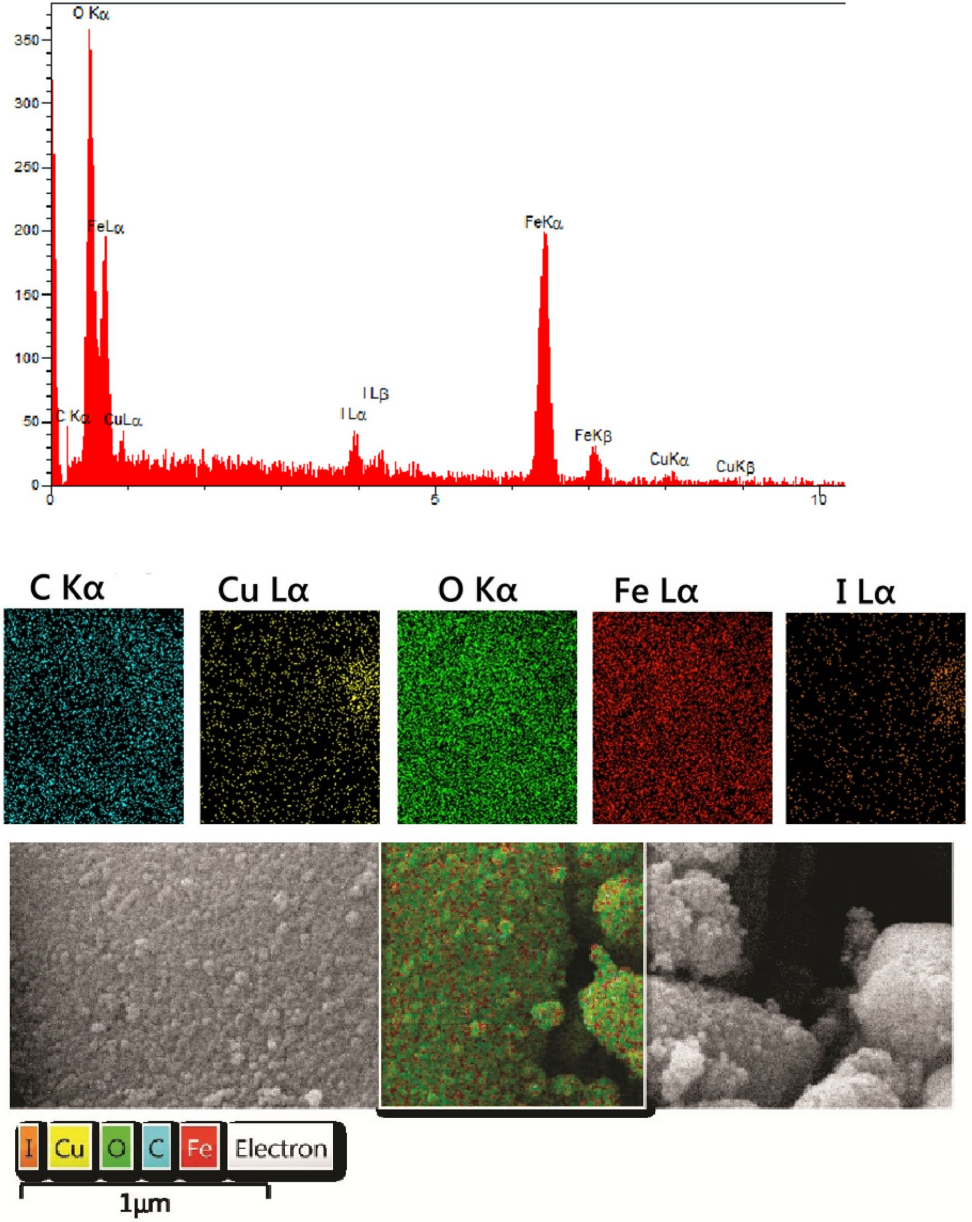
(**A**) Energy-dispersive X-ray spectroscopy (EDX), (**B**) Elemental mapping analysis of Fe_3_O_4_@CQD@CuI.

EDX and elemental mapping analysis confirmed the presence of iron (Fe), carbon (C), oxygen (O), copper (Cu) and iodine (I) components in the catalyst based on Fig. 6. The results of elemental mapping analysis also revealed that the elements had a uniform distribution in the catalyst structure.

**Figure 7:**
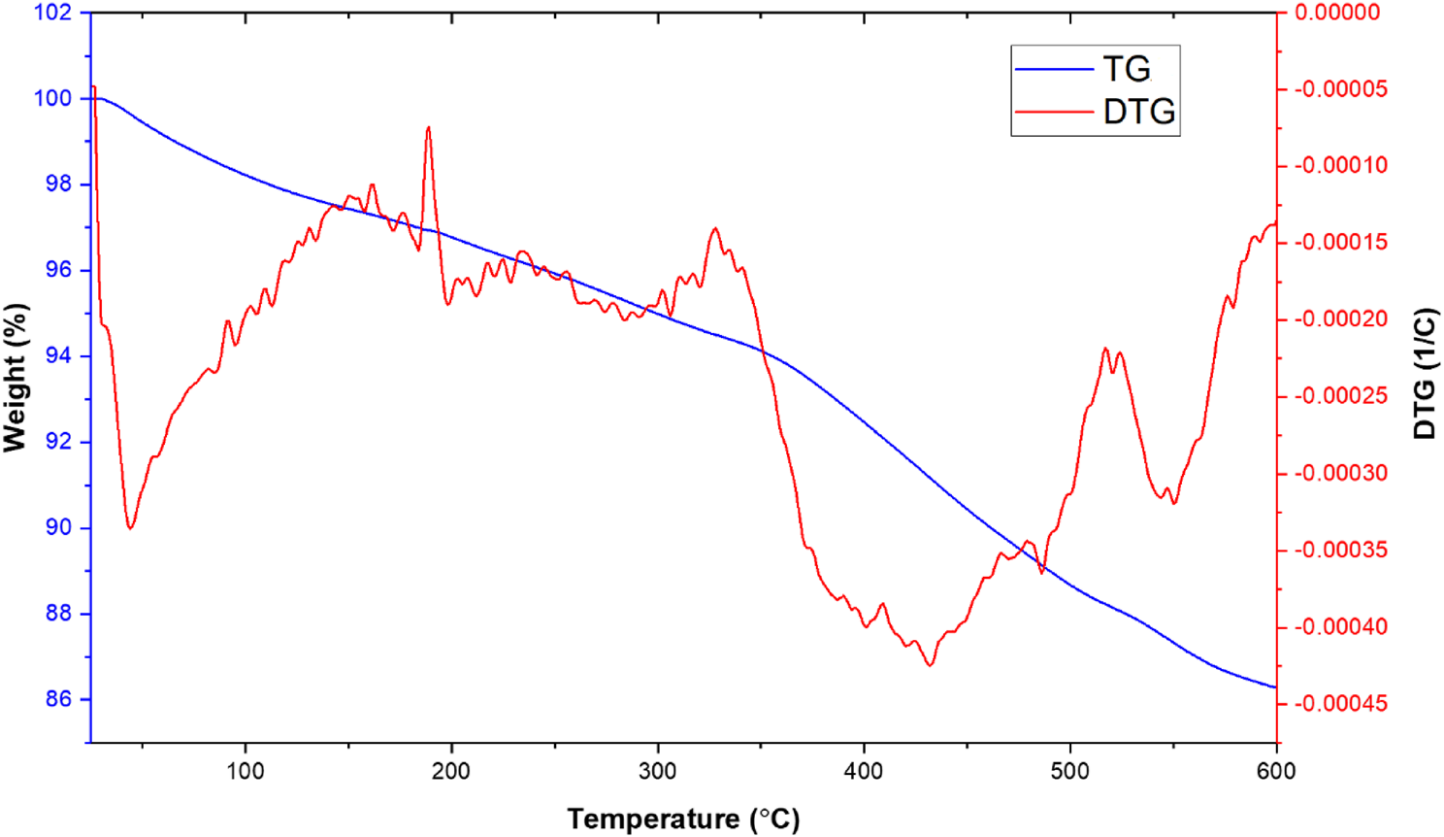
TGA and DTG analysis of Fe_3_O_4_@CQD@CuI.

The pattern of thermal gravimetric (TGA-DTG) curve of Fe_3_O_4_@CQD@CuI as shown in Fig. 7, revealed three stages of weight loss for Fe_3_O_4_@CQD@CuI up to 600 °C. The first weight loss (1–2%) which observed between 25 and 100 °C was related to the removal of moisture from the catalyst structure. The second weight loss (5–6%) appeared at 400 °C, which was attributed to the release of CO_2_ groups due to C–C bond breaking of the aromatic ring and the carboxylic acid groups. The final stage of weight loss (10–12%) at 600 °C was assigned to the decomposition of the carbon quantum dot coated on the Fe_3_O_4_. In addition, The Differential Thermogravimetric (DTG) curve shows endothermic peaks in this region which confirms the successful chemical adsorption of organic complex layers via chemical bonding on the support.

**Figure 8:**
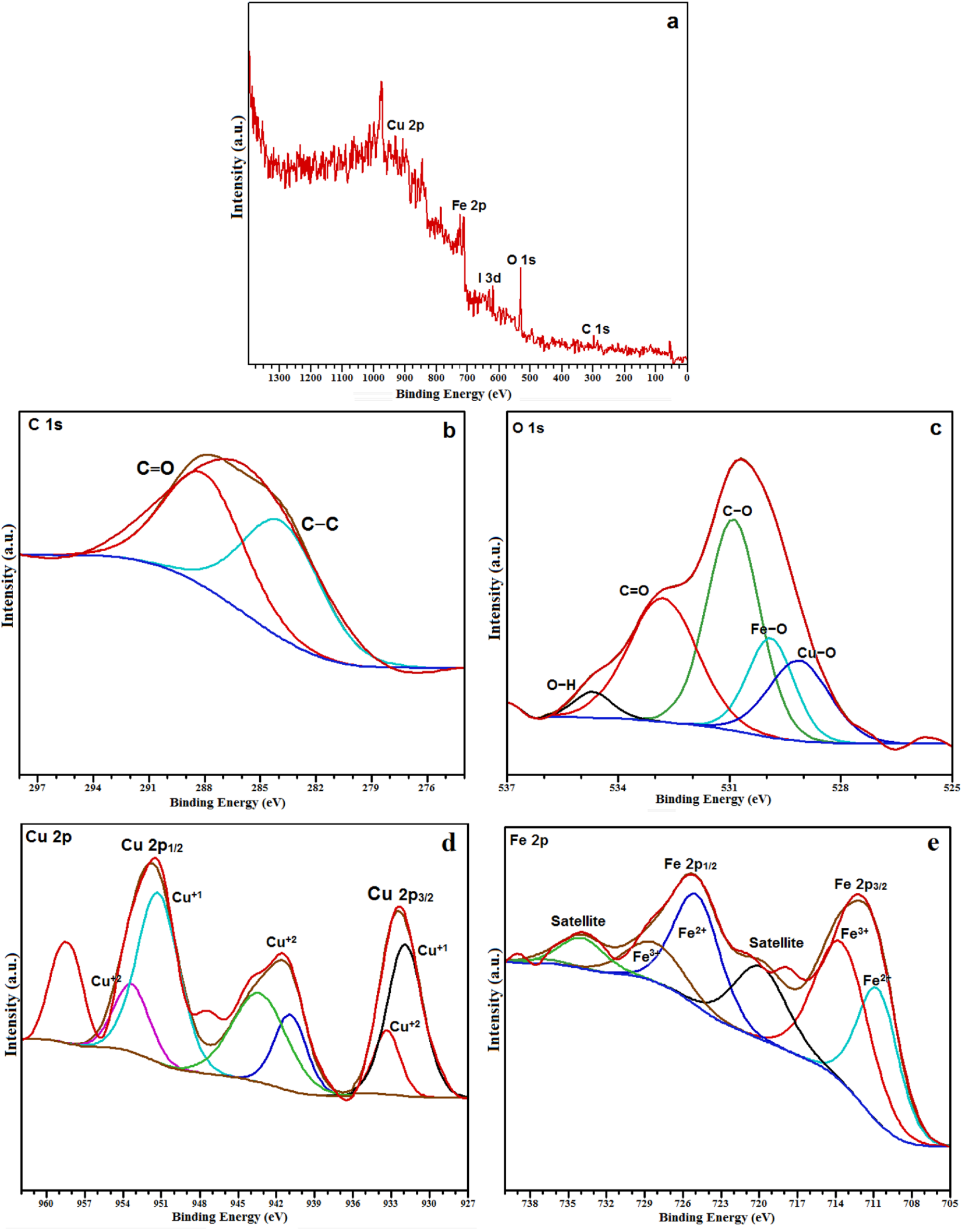
XPS spectrum of Fe_3_O_4_@CQD@CuI; XPS survey spectrum (**a**), C 1s (**b**), O1s (**c**), Cu 2p (**d**) and Fe 2p (**e**).

XPS analysis is a powerful surface sensitive technique that has been used to confirm the chemical composition, purity, and oxidation states of element. The C 1s (carbon 1s) peak at 284.60 eV was used as a reference for the calibration of all binding energies. Figure 8a shows the wide scan spectrum XPS (survey spectrum) of the Fe_3_O_4_@CQD@CuI nanocatalyst with characteristic peaks of the elements including copper (Cu), oxygen (O), carbon (C), iodine (I), and iron (Fe). Figure 8b–e show the high-resolution spectra of C 1s, O 1s, Cu 2p, and Fe 2p, respectively. In Fig. 8b, two peaks at 284.18 and 288.41 eV can be attributed to the bonds C–C and C=O^49^. The spectral band of O 1s consists of five peaks including 534.72 eV, 532.84 eV, 530.92 eV, 529.93 eV, and 529.11 eV, which are related to the H–O, C=O, C–O, Cu–O and Fe–O bonds, respectively (Fig. 8c)^50^. Figure 8d shows the spectrum of the nucleus of a copper atom on the surface of the catalyst. The peak at 932.37 eV and 951.61 eV are related to Cu 2p_3/2_ and Cu 2p_1/2_, respectively. Also, the appearance of two satellite peaks at 95.53 eV and 942.43 eV confirms the existence of Cu–O bonds^51–53^. The XPS results of Cu 2p indicate that copper ions exist in two oxidation states. The 931.92 and 951.45 eV bond energy bands are assigned to Cu^+1^ 2p_3/2_ and Cu^+1^ 2p_1/2_, respectively, and the peaks of 933.37, 937–946.5 (satellite peaks), and 953.46 eV are corresponded to Cu^+2^ 2p_3/2_ and Cu^+2^ 2p_1/2_ (Fig. 8d)^54^. The two spectral bands at 712.20 eV and 725.47 eV are related to Fe 2p_3/2_ and Fe 2p_1/2_ (Fig. 8e). The two weak satellite peaks at 720.04 eV and 734.24 eV indicate the purity and presence of the Fe_3_O_4_ phase in the Fe_3_O_4_@CQD@CuI catalyst. Also, the presence of Fe^+3^ and Fe^+2^ species, which are the characteristics of Fe_3_O_4_ nanoparticles, is shown in the Fig. 8e^55^.

**Figure 9:**
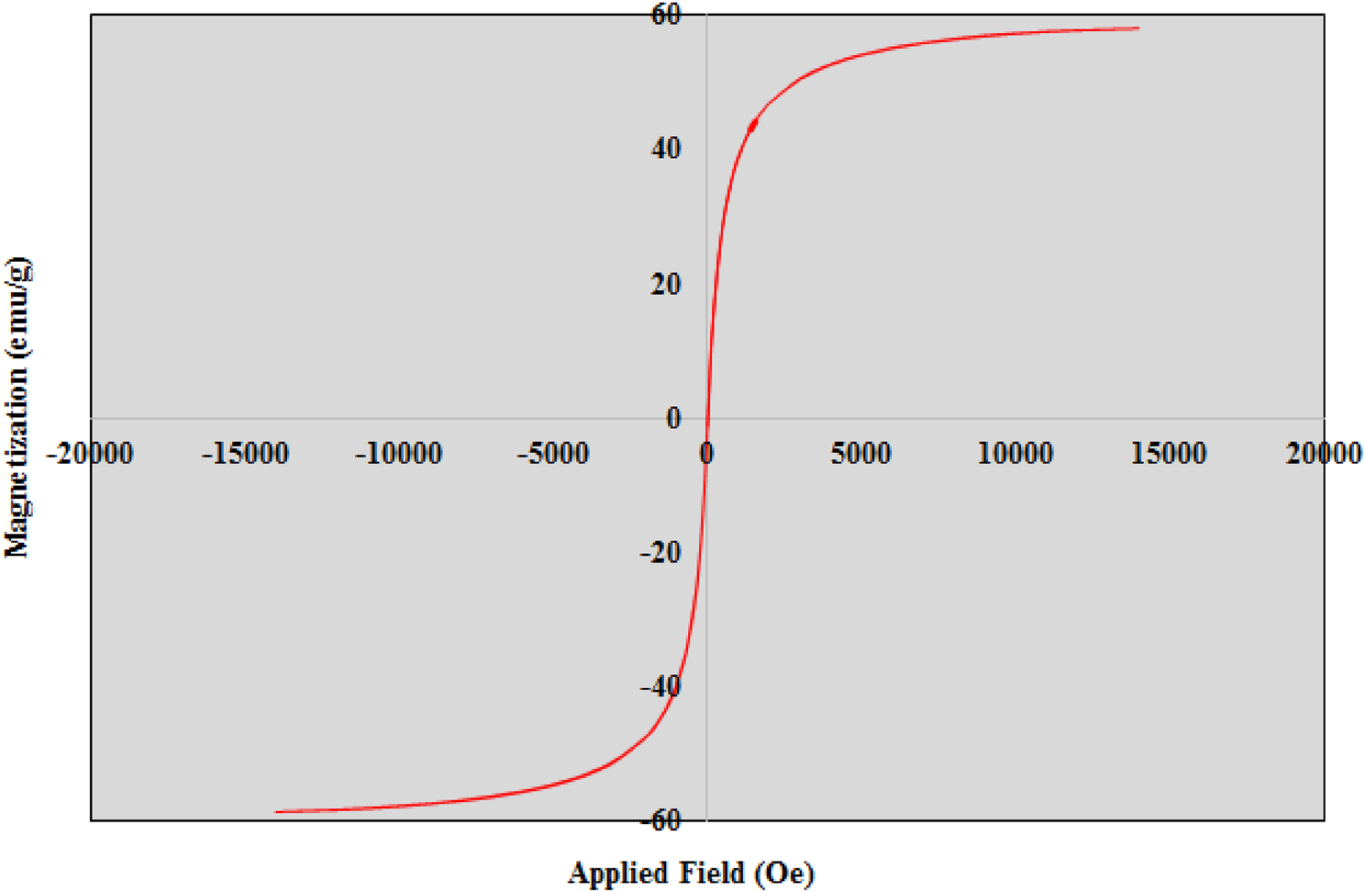
The vibrating sample magnetometer (VSM) of Fe_3_O_4_@CQD@CuI.

An attempt was made to investigate magnetic measurements of Fe_3_O_4_@CQD@CuI at the room temperature using vibrating sample magnetometer (VSM). As shown in Fig. 9, based on magnetization curves, the saturation of the obtained catalyst dropped to 58.11 emu g^−1^.

### Application of Fe_3_O_4_@CQD@CuI as magnetic nanoparticle (MNP) catalyst in the synthesis of kojic acid based dihydropyrano[3,2-*b*]pyran and new kojic acid-triazole hybrid based dihydropyrano[3,2-*b*]pyran derivatives

After the synthesis and fully characterization of Fe_3_O_4_@CQD@CuI, Beginning to investigate its catalytic activity, it was used as a MNP catalyst for the synthesis of kojic acid based dihydropyrano[3,2-b]pyran derivatives in a multi component reaction via a condensation reaction of suitable starting materials. In the following, the new triazole compounds were synthesized via the click reaction using kojic acid based dihydropyrano[3,2-b]pyran derivatives having an acetylene group in the presence of MNP catalysts Fe_3_O_4_@CQD@CuI.

In order to optimize the reaction condition, the three-component reaction were performed between kojic acid (1 mmol, 0.142 g), malononitrile (1.1 mmol, 0.072 g) and benzaldehyde (1 mmol, 0.106 g) to synthesis kojic acid based dihydropyrano[3,2-*b*]pyran derivatives under various conditions including different temperatures, reflux and ultrasonic in water, acetonitrile, ethanol, ethyl acetate and *n*-hexane (5 mL) as solvents in the presence a catalytic amount of Fe_3_O_4_@CQD@CuI. Based on the results shown in Table 1, a mixture of water and ethanol (1:2) as solvent and ultrasonic condition at 50 °C was the best condition of reaction for the synthesis of kojic acid based dihydropyrano[3,2-b]pyrans derivatives (Table 1, entry 9). Any change did not appearance in efficiency by increasing the amount of catalyst and the temperature (Table 1, entry 10 and 11, respectively). At the ultrasonic condition, by decreasing the temperature, a decrease in the reaction efficiency was observed (Table 1, entry 8) whereas by decreasing the amount of catalyst, the reaction efficiency was decreased (Table 1, entry 12). The product was obtained with lower efficiency under longer time when the reaction carried out in non-ultrasonic conditions (Reflux). According to the obtained data, the ultrasonic waves reduce the time and increase the efficiency compared to other conditions in the synthesis of dihydropyrano[3,2-b]pyrans compounds.

**Table 1:** Effect of different amounts of catalyst, temperature, ultrasonic and solvent (5 mL) in the synthesis of dihydropyrano[3,2-*b*]pyran.


Entry	Solvent	Temp. (°C)	Catalyst (mg)	Time (min)	Yield %
1	EtOH	Reflux	6	60	70
2	H_2_O	Reflux	6	60	43
3	*n*-Hexane	Reflux	6	60	Trace
4	Ethyl acetate	Reflux	6	60	24
5	CH_3_CN	Reflux	6	60	18
6	EtOH, H_2_O (1:1)	Reflux	6	5	42
7	EtOH, H_2_O (2:1)	Reflux	6	5	80
8	EtOH, H_2_O (2:1)	r.t. US	6	5	60
**9**	**EtOH, H**_**2**_**O (2:1)**	**50, US**	**6**	**5**	**90**
10	EtOH, H_2_O (2:1)	70, US	6	5	90
11	EtOH, H_2_O (2:1)	50, US	10	5	90
12	EtOH, H_2_O (2:1)	50, US	3	5	86

Significant values are in bold.

**Figure 10:**
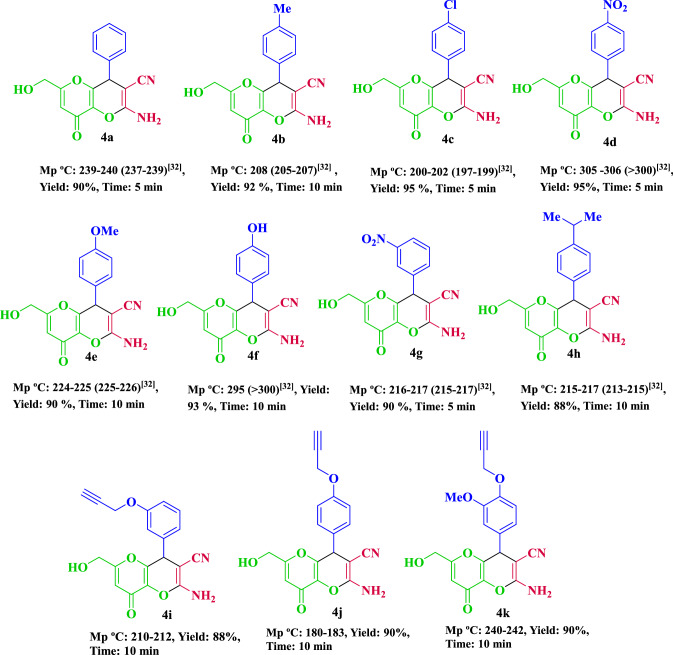
Synthesis of dihydropyrano[3,2-*b*]pyrans using Fe_3_O_4_@CQD@CuI.

After determining the best reaction conditions for synthesis of dihydropyrano[3,2-*b*]pyrans, a wide range of aromatic aldehydes having the electron-donating and electron-withdrawing groups were synthesized (Fig. 10). As specified in Table 2, the aldehydes with electron-withdrawing groups compared to electron-donating groups resulted in higher efficiencies in this reaction.

**Table 2:** Effect of different amounts of catalysts, temperature and solvent (5 mL) in the triazole-dihydropyrano[3,2-*b*]pyran.


Entry	Solvent	Conditions	Catalyst (mg)	Time (h)	Yield (%)
1	H_2_O	r.t.	10	4	10
2	t-BuOH	r.t.	10	6	Trace
3	DMF	r.t.	10	6	25
4	MeOH	r.t.	10	4	65
5	t-BuOH: H_2_O (2:1)	r.t.	10	6	45
6	H_2_O	r.t., US	10	2	80
7	MeOH	r.t., US	10	2.5	70
8	t-BuOH	r.t., US	10	3	35
9	DMF	r.t., US	10	1.5	50
**10**	**H**_**2**_**O**	**60, US**	**10**	**1**	**93**
11	H_2_O	80, US	10	1	93
12	H_2_O	60, US	5	1.5	75
13	H_2_O	60, US	15	1.5	93

Significant values are in bold.

**Figure 11:**
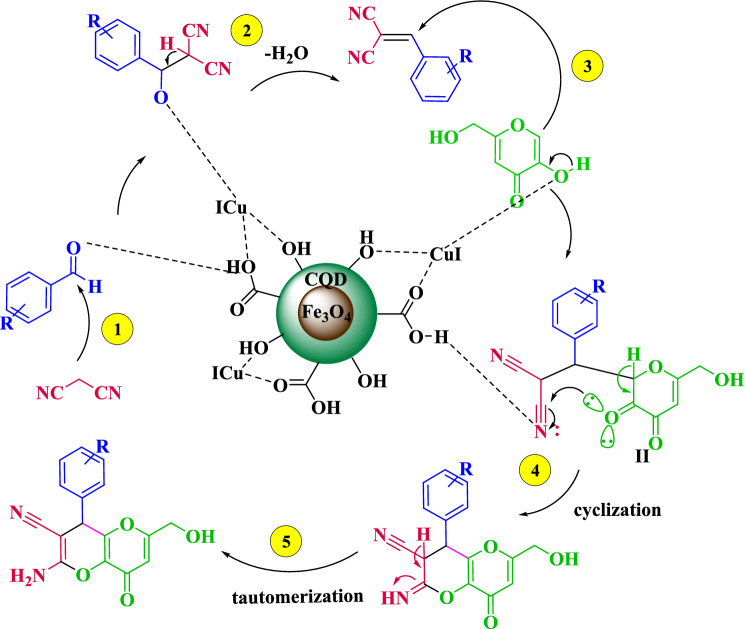
Proposed mechanism for the synthesis kojic acid based dihydropyrano-pyran using Fe_3_O_4_@CQD@CuI.

This observation can be excused on the basis of the acceptable mechanism suggested for the synthesis of kojic acid based dihydropyrano[3,2-*b*]pyrans using Fe_3_O_4_@CQD@CuI catalyst as shown in Fig. 11. According to the reaction pathway, aldehyde is initially activated by the acidic and hydroxyl sites of the catalyst, then reacts with malononitrile to afford intermediate (I) by removing one water molecule. Then, intermediate (I) as Michael acceptor reacts with 2-hydroxynaphtalen-1,4-dione, 5-hydroxy-2-(hydroxymethyl)-4*H*-pyran-4-one to form intermediate (II). Finally, intermediate (II) to give the desired corresponding dihydropyrano[3,2-*b*]pyrans will be will be undergone the intramolecular cyclization and tautomerization.

**Figure 12:**
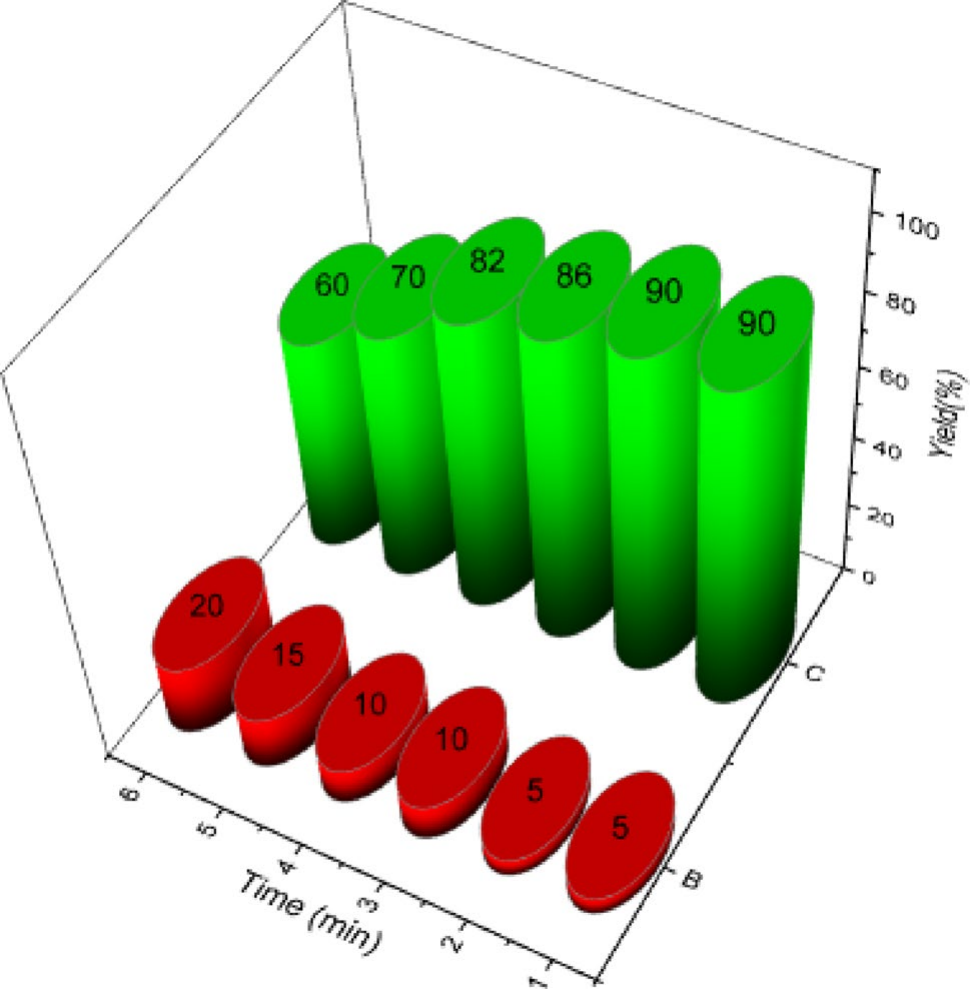
Recyclability of Fe_3_O_4_@CQD@CuI for the synthesis dihydropyrano-pyran compounds.

The recovery and reusing capability of the catalyst in a model reaction in the synthesis of kojic acid based dihydropyrano-pyran derivatives was investigated. Kojic acid (1 mmol, 0.142 g), malononitrile (1.1 mmol, 0.072 g) and benzaldehyde (1 mmol, 0.106 g) were used for this purpose. The results showed that the MNP catalyst of Fe_3_O_4_@CQD@CuI could be recovered and reused up to 5 times without any noticeable loss of catalytic activity (Fig. 12).

After the high efficiency revelation of this catalyst in the synthesis of kojic acid based dihydropyrano[3,2-*b*]pyrans different derivatives, a new class of kojic acid-triazole hybrids were investigated using acetylene of dihydropyrano[3,2-*b*]pyrans derivatives. In order to synthesize triazole derivatives, the reaction between 2-amino-6-(hydroxymethyl)-8-oxo-4-(4-(prop-2-yn-1-yloxy)phenyl)-4,8-dihydropyrano[3,2-b]pyran-3-carbonitrile (1 mmol, 0.350) with benzyl chloride derivatives (1.1 mmol) and sodium azide (1.5 mmol, 0.0975 g) under various conditions including different solvents (water, dimethylformamide (DMF), methanol and tert-Butyl alcohol), temperatures and ultrasonic in the presence of a catalytic amount of Fe_3_O_4_@CQD@CuI were tested which its results shown in Table 2. The results displayed that water as solvent and ultrasonic condition at 60 °C was the best reaction condition of choices for the production of kojic acid-triazole based dihydropyrano[3,2-*b*]pyran in click reaction (Table 2, entry 10). The product was obtained in stirring condition in water with lower efficiency than ultrasonic condition (Table 2, entry 1). Lowering the temperature and the catalyst values led to low efficiency while no increasing efficiency was observed with increasing them (Table 2, entries 11–13).

**Figure 13:**
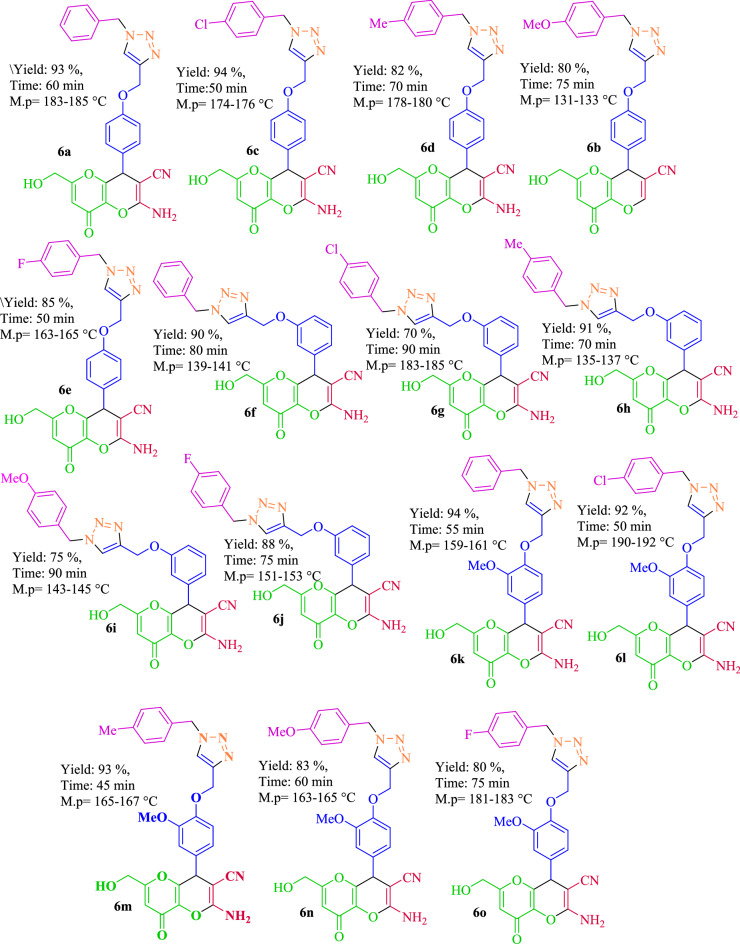
Synthesis of triazole-dihydropyrano[3,2-*b*]pyran using Fe_3_O_4_@CQD@CuI.

After determining the optimal condition, it was used to evaluate the efficiency of the catalyst in the synthesis of new triazole compounds using benzyl halide derivatives in reaction with dihydropyrano[3,2-*b*]pyrans derivatives containing acetylene group. The results revealed that the products had high efficiency and low reaction time (Fig. 13).

**Figure 14:**
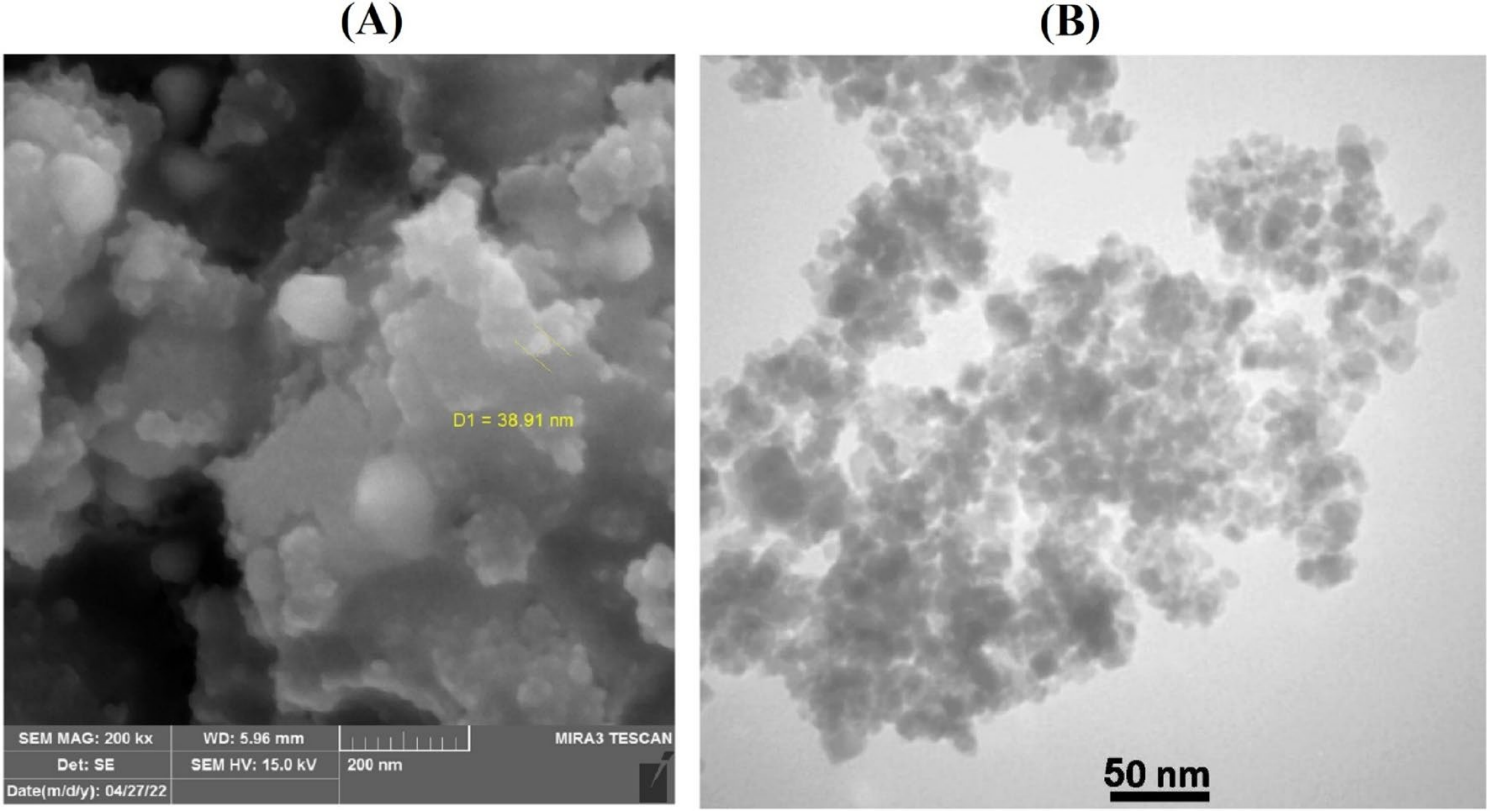
(**A**) SEM and (**B**) TEM images of recovered Fe_3_O_4_@CQD@CuI.

To evaluate the performance of Fe_3_O_4_@CQD@CuI as catalyst for the synthesis of kojic acid-triazole based dihydropyrano-pyran derivatives, the various homogeneous and heterogeneous catalysts containing copper were used for the click reaction between 2-amino-6-(hydroxymethyl)-8-oxo-4-(4-(prop-2-yn-1-yloxy)phenyl)-4,8-dihydropyrano[3,2-b]pyran-3-carbonitrile (1 mmol, 0.350 g), sodium azide (1.5 mmol, 0.0975 g) and benzyl chloride derivatives (1.1 mmol, 0.138 g) under ultrasonic condition in the water as a solvent at 60 °C temperature (Table 3). The results in Table 3 predicate that Fe_3_O_4_@CQD@CuI is the best catalyst for the synthesis of kojic acid-triazole based dihydropyrano-pyran derivatives. Furthermore, the spent catalyst was characterized after the 5th catalytic cycle using SEM and TEM analyses. The morphology and particle size of the Fe_3_O_4_@CQD@CuI after the 5th catalytic cycle was not changed based on SEM and TEM images prior and after using in the reaction (Fig. 14).

**Table 3:** Evaluation of various catalyst for the synthesis of triazole-dihydropyrano-pyran in click reaction with Fe_3_O_4_@CQD@CuI in water under ultrasonic conditions.

Entry	Catalyst	Amount of catalyst (mol%)	Yield (%)
1	CuI	10	55
2	CuCl	10	60
3	Cu(OAc)_2_/ascorbic acid	10	20
4	CuSO_4_/ascorbic acid	10	55
5	CuO	10	–
6	CQD@CuI	10 mg (not recyclable)	93
7	Fe_3_O_4_@CQD@CuI	10 mg (recyclable)	93

## Conclusion

In this research, we have designed and synthesized the NMP Fe_3_O_4_@CQD@CuI having carboxylic acid groups and copper iodide salt as an acid high efficiency catalyst for the synthesis of kojic acid based dihydropyrano-pyran and kojic acid–triazole based triazol-dihydropyrano-pyran compounds that favorably combines the properties Brønsted and Lewis acid and advantages of nanomagnetics catalyst in a three-component and the click reactions. The considerable advantages of this method are easily catalyst removal from the reaction medium using an external magnetic field, its reusing capability and high efficiency in lower time reaction.

## Supplementary Information

Supplementary Information.
