# Supplementary material for: Ultrasound-assisted synthesis of kojic acid-1,2,3-triazole based dihydropyrano[3,2-b]pyran derivatives using Fe3O4@CQD@CuI as a novel nanomagnetic catalyst
Source: Sci Rep. 2022 Nov 19;12:19917. doi: 10.1038/s41598-022-24089-6 (PMC9675794; doi:10.1038/s41598-022-24089-6)

**Ultrasound-assisted synthesis of kojic acid-1,2,3-triazole based dihydropyrano[3,2-*b*]pyran derivatives using Fe_3_O_4_@CQD@CuI as a novel nanomagnetic catalyst**

Zahra Najafi^a^*, Soheila Esmaeili^b^, Behnam Khaleseh^a^, Saeed Babaee^b^, Mehdi Khoshneviszadeh^c^, Gholamabbas Chehardoli^a^, Tahmineh Akbarzadeh^d^

*^b^ Department of Medicinal Chemistry, School of Pharmacy, Hamadan University of Medical Sciences, Hamadan, Iran*

*^a^ Department of Organic Chemistry, Faculty of Chemistry, Bu-Ali Sina University, Hamedan, Iran*

*^c^ Department of Medicinal Chemistry, School of Pharmacy, Shiraz University of Medical Sciences, Shiraz, Iran*

*^d^ Department of Medicinal Chemistry, Faculty of Pharmacy, Tehran University of Medical Sciences, Tehran, Iran*

*najafi.zch@gmail.com or z.najafi@umsha.ac.ir*

**2-Amino-6-(hydroxymethyl)-8-oxo-4-(4-(prop-2-yn-1-yloxy)phenyl)-4,8-dihydropyrano[3,2-b]pyran-3-carbonitrile (4i)**

Cream solid; m.p= 180-183 °C, FT-IR (KBr): υ (cm^-1^)= 3408, 3278, 3194, 2192, 1737, 1639, 1214. ^1^H NMR (300 MHz, DMSO-*d*_6_) *δ_ppm_*: 7.16 (d, *J* = 6.8 Hz, 2H), 7.14 (s, 2H), 6.93 (d, *J* = 8.7 Hz, 2H), 6.26 (s, 1H), 5.64 (t, *J* = 6.0 Hz, 1H), 4.73 (d, *J* = 2.3 Hz, 2H), 4.68 (s, 1H), 4.19 – 4.01 (m, 2H), 3.50 (t, *J* = 2.3 Hz, 1H). ^13^C NMR (76 MHz, DMSO-*d*_6_) *δ_ppm_*: 170.1, 168.7, 159.6, 157.3, 149.7, 136.7, 134.1, 129.4, 119.8, 115.6, 111.8, 79.7, 78.8, 59.6, 56.4, 55.9, 36.3. Anal. calcd. for C_19_H_14_N_2_O_5_: C, 65.14; H, 4.03; N, 8.00; Found: C: 65.23; H: 4.03; N: 8.86.

**2-Amino-6-(hydroxymethyl)-8-oxo-4-(3-(prop-2-yn-1-yloxy)phenyl)-4,8-dihydropyrano[3,2-*b*]pyran-3-carbonitrile (4j)**

Cream solid; m.p = 210- 212 °C, FT-IR (KBr): υ (cm^-1^)= 3423, 3371, 3280, 3185, 2190, 1642, 1596, 1212. ^1^H NMR (300 MHz, DMSO-*d*_6_) *δ_ppm_*: 7.36 (t, *J* = 8.0 Hz, 1H), 7.28 (s, 2H), 6.98 (dd, *J* = 7.9, 2.6 Hz, 1H), 6.94 – 6.90 (m, 2H), 6.37 (s, 1H), 5.73 (s, 1H), 4.82 (d, *J* = 2.4 Hz, 2H), 4.81 – 4.79 (m, 1H), 4.29 – 4.13 (m, 2H), 3.57 (t, *J* = 2.3 Hz, 1H). ^13^C NMR (76 MHz, DMSO-*d*_6_) *δ_ppm_*: 170.1, 168.7, 159.8, 158.0, 149.3, 142.9, 136.9, 130.6, 121.0, 119.7, 115.1, 114.2, 111.9, 79.6, 78.8, 59.6, 56.0, 55.9, 40.7. Anal. calcd. for C_19_H_14_N_2_O_5_: C, 65.14; H, 4.03; N, 8.00; Found: C: 65.13; H: 4.08; N: 7.88.

**2-Amino-6-(hydroxymethyl)-4-(3-methoxy-4-(prop-2-yn-1-yloxy)phenyl)-8-oxo-4,8-dihydropyrano[3,2-*b*]pyran-3-carbonitrile (4k)**

Brown solid; m.p = 240-242 °C, FT-IR (KBr): υ (cm^-1^)= 3360, 3194, 2197, 1650,1517, 1217. ^1^H NMR (300 MHz, DMSO-*d*_6_) *δ_ppm_*: 7.25 (s, 2H), 7.06 (d, *J* = 8.3 Hz, 1H), 6.93 (s, 1H), 6.81 (d, *J* = 8.3 Hz, 1H), 6.36 (s, 1H), 5.73 (t, *J* = 6.0 Hz, 1H), 4.80 (s, 2H), 4.77 (s, 1H), 4.31 – 4.14 (m, 2H), 3.79 (s, 3H), 3.58 (s, 1H). ^13^C NMR (76 MHz, DMSO-*d*_6_) *δ_ppm_*: 170.1, 168.6, 159.7, 149.7, 149.5, 146.6, 136.7, 134.7, 120.0, 119.9, 114.5, 112.1, 111.8, 79.8, 78.8, 59.6, 56.4, 56.1, 56.0, 40.3. Anal. calcd. for C_20_H_16_N_2_O_6_: C, 63.16; H, 4.24; N, 7.37; Found: C: 63.21; H: 4.25; N: 7.30.

**3-Amino-4-(4-((1-benzyl-1*H*-1,2,3-triazol-4-yl)methoxy)phenyl)-6-(hydroxymethyl)-8-oxo-4,8-dihydropyrano[3,2-*b*]pyran-2-carbonitrile (6a)**

Yellow solid; m.p= 183-185 °C, FT-IR (KBr): υ (cm^-1^)= 3425, 2924, 2193, 1641, 1509, 1410. ^1^H NMR (400 MHz, DMSO-*d*_6_) *δ_ppm_*: δ 8.32 (s, 1H), 7.44 – 7.33 (m, 7H), 7.27 (s, 2H), 7.04 (d, *J* = 7.8 Hz, 1H), 6.94 (s, 1H), 6.89 (d, *J* = 7.8 Hz, 1H), 6.36 (s, 1H), 5.63 (s, 2H), 5.16 (s, 2H), 4.79 (s, 1H), 4.27 – 4.13 (m, 2H). ^13^C NMR (100 MHz, DMSO-*d*_6_) *δ_ppm_*: 162.6, 157.7, 150.9, 144.4, 131.4, 131.0, 127.7, 127.2, 125.2, 125.0, 120.7, 119.4, 116.0, 115.8, 115.7, 105.7, 59.7, 56.9, 55.1, 39.8. Anal. calcd. for C_26_H_21_N_5_O_5_: C, 64.59; H, 4.38; N, 14.49; Found: C: 64.51; H: 4.42; N: 14.52.

**2-Amino-4-(4-((1-(4-chlorobnzyl)-1*H*-1,2,3-triazol-4-yl)methoxy)phenyl)-6-(hydroxymethyl)-8-oxo-4,8-dihydropyrano[3,2-*b*]pyran-3-carbonitrile (6b)**

Yellow solid; m.p= 174-176 °C, FT-IR (KBr): υ (cm^-1^)= 3229, 2193, 1644, 1509, 1409. ^1^H NMR (400 MHz, DMSO-*d*_6_) *δ_ppm_*: δ 8.29 (s, 1H), 7.44 (d, *J* = 8.4 Hz, 2H), 7.34 (d, *J* = 8.4 Hz, 2H), 7.20 (s, 2H), 7.19 (d, *J* = 7.2 Hz, 2H), 7.03 (d, *J* = 7.2 Hz, 2H), 6.31 (s, 1H), 5.61 (s, 2H), 5.12 (s, 2H), 4.72 (s, 1H), 4.24 – 4.08 (m, 2H). ^13^C NMR (100 MHz, DMSO-*d*_6_) *δ_ppm_*: 170.1, 168.8, 159.7, 158.2, 149.8, 143.4, 138.1, 136.7, 133.7, 133.5, 129.8, 129.4, 128.6, 125.1, 119.9, 115.5, 111.9, 61.6, 56.4, 53.2, 40.7. Anal. calcd. for C_26_H_20_ClN_5_O_5_: C, 60.30; H, 3.89; N, 13.52; Found: C: 60.19; H: 3.80; N: 13.24.

**2-Amino-6-(hydroxymethyl)-4-(4-((1-(4-methylbenzyl)-1*H*-1,2,3-triazol-4-yl)methoxy)phenyl)-8-oxo-4,8-dihydropyrano[3,2-*b*]pyran-3-carbonitrile (6c)**

Brown solid; m.p= 178-180 °C, FT-IR (KBr): υ (cm^-1^)= 3318, 2920, 2193, 1645, 1509. ^1^H NMR (400 MHz, DMSO-*d*_6_) *δ_ppm_*: δ 8.26 (s, 1H), 7.28 – 7.14 (m, 8H), 7.03 – 7.05 (m, 2H), 6.33 (s, 1H), 5.56 (s, 2H), 5.12 (s, 2H), 4.74 (s, 1H), 4.24 – 4.10 (m, 2H), 2.28 (s, 3H). ^13^C NMR (100 MHz, DMSO-*d*_6_) *δ_ppm_*: 169.6, 168.2, 159.1, 157.6, 149.2, 142.8, 137.5, 136.1, 133.2, 132.9, 131.6, 129.2, 128.8, 128.0, 124.5, 119.3, 115.0, 111.3, 61.1, 59.1, 55.9, 52.6, 38.0, 20.7. Anal. calcd. for C_27_H_23_N_5_O_5_: C, 65.18; H, 4.66; N, 14.08; Found: C: 65.19; H: 4.70; N: 14.04.

**2-Amino-6-(hydroxymethyl)-4-(4-((1-(4-methoxybenzyl)-1*H*-1,2,3-triazol-4-yl)methoxy)phenyl)-8-oxo-4,8-dihydropyrano[3,2-*b*]pyran-3-carbonitrile (6d)**

Brown solid; m.p= 131-133 °C, FT-IR (KBr): υ (cm^-1^)= 3429, 2926, 2197, 1640, 1511, 1385. ^1^H NMR (400 MHz, DMSO-*d*_6_) *δ_ppm_*: δ 8.25 (s, 1H), 7.31 (d, *J* = 8.7 Hz, 2H), 7.20 (d, *J* = 8.7 Hz, 3H), 7.04 (d, *J* = 8.7 Hz, 2H), 6.93 (d, *J* = 8.7 Hz, 2H), 6.33 (s, 1H), 5.53 (s, 2H), 5.11 (s, 2H), 4.74 (s, 1H), 4.10-415 (m, 2H), 3.74 (s, 3H). ^13^C NMR (100 MHz, DMSO-*d*_6_) *δ_ppm_*: 169.6, 168.2, 159.1, 157.6, 149.2, 136.1, 133.9, 133.2, 129.6, 128.8, 127.9, 124.4, 119.3, 114.9, 114.3, 114.1, 113.4, 111.3, 61.1, 59.0, 55.1, 45.5, 41.3. Anal. calcd. for C_27_H_23_N_5_O_6_: C, 63.15; H, 4.51; N, 13.64; Found: C: 63.19; H: 4.45; N: 13.58.

**2-Amino-4-(4-((1-(4-fluorobenzyl)-1*H*-1,2,3-triazol-4-yl)methoxy)phenyl)-6-(hydroxymethyl)-8-oxo-4,8-dihydropyrano[3,2-*b*]pyran-3-carbonitrile (6e)**

Brown solid; m.p= 163-165 °C, FT-IR (KBr): υ (cm^-1^)= 3416, 2207, 1605, 1510, 1228. ^1^H NMR (400 MHz, DMSO-*d*_6_) *δ_ppm_*: 8.30 (s, 1H), 7.42 (d, *J* = 5.4 Hz, 1H), 7.40 (d, *J* = 5.4 Hz, 2H), 7.22 – 7.19 (m, 5H), 7.04 (d, *J* = 8.7 Hz, 2H), 6.33 (s, 1H), 5.61 (s, 2H), 5.13 (s, 2H), 4.74 (s, 1H), 4.17 – 4.11 (m, 2H). ^13^C NMR (100 MHz, DMSO-*d*_6_) *δ_ppm_*: 170.1, 168.7, 159.8, 149.6, 147.6, 143.4, 136.7, 135.5, 134.2, 132.8, 130.9, 130.8, 125.3, 124.7, 120.1, 116.7, 116.4, 116.2, 116.0, 114.1, 111.8, 62.1, 59.6, 56.0, 46.2. Anal. calcd. for C_26_H_20_FN_5_O_5_: C, 62.27; H, 4.02; N, 13.97; Found: C: 62.22; H: 4.10; N: 13.98.

**2-Amino-4-(3-((1-benzyl-1*H*-1,2,3-triazol-4-yl)methoxy)phenyl)-6-(hydroxymethyl)-8-oxo-4,8-dihydropyrano[3,2-*b*]pyran-3-carbonitrile (6f)**

Yellow solid; m.p= 139-141 °C, FT-IR (KBr): υ (cm^-1^)= 3320, 2194, 1643, 1639, 1097. ^1^H NMR (400 MHz, DMSO-*d*_6_) *δ_ppm_*: 8.32 (s, 1H), 7.44 – 7.33 (m, 7H), 7.27 (s, 2H), 7.04 (d, *J* = 8.6 Hz, 1H), 6.94 (s, 1H), 6.89 (d, *J* = 7.8 Hz, 1H), 6.36 (s, 1H), 5.63 (s, 2H), 5.16 (s, 2H), 4.79 (s, 1H), 4.27 – 4.13 (m, 2H). ^13^C NMR (101 MHz, DMSO-*d*_6_) *δ_ppm_*: 163.6, 162.2, 157.7, 150.8, 144.1, 135.0, 131.4, 131.3, 131.2, 130.1, 128.5, 127.7, 127.1, 124.3, 120.6, 119.5, 117.4, 116.1, 115.9, 105.7, 61.6, 58.0, 55.1, 39.9. Anal. calcd. for C_26_H_21_N_5_O_5_: C, 64.59; H, 4.38; N, 14.49; Found: C: 64.42; H: 4.40; N: 14.55.

**2-Amino-4-(3-((1-(4-chlorobenzyl)-1*H*-1,2,3-triazol-4-yl)methoxy)phenyl)-6-(hydroxymethyl)-8-oxo-4,8-dihydropyrano[3,2-*b*]pyran-3-carbonitrile (6g)**

Brown solid; m.p= 183-185 °C, FT-IR (KBr): υ (cm^-1^)= 3430, 2925, 2193, 1641, 1601, 1508, 1408, 1256. ^1^H NMR (400 MHz, DMSO-*d*_6_) *δ_ppm_*: 8.32 (s, 1H), 7.46 (d, *J* = 8.5 Hz, 2H), 7.36 (d, *J* = 8.5 Hz, 2H), 7.32 (d, *J* = 7.9 Hz, 1H), 7.25 (s, 2H), 7.02 (d, *J* = 7.9, Hz, 1H), 6.92 – 6.90 (m, 1H), 6.87 (d, *J* = 7.9 Hz, 1H), 6.34 (s, 1H), 5.63 (s, 2H), 5.14 (s, 2H), 4.77 (s, 1H), 4.24 – 4.12 (m, 2H). ^13^C NMR (100 MHz, DMSO-*d*_6_) *δ_ppm_*: 162.9, 162.1, 142.3, 134.9, 131.8, 131.5, 129.9, 129.6, 129.4, 128.8, 128.7, 128.7, 125.0, 122.0, 115.2, 114.7, 98.3, 61.4, 56.1, 52.0, 40.1. Anal. calcd. for C_26_H_20_ClN_5_O_5_: C, 60.30; H, 3.89; Cl, 6.84; N, 13.52; Found: C: 60.30; H: 3.40; N: 13.56.

**2-Amino-6-(hydroxymethyl)-4-(3-((1-(4-methylbenzyl)-1*H*-1,2,3-triazol-4-yl)methoxy)phenyl)-8-oxo-4,8-dihydropyrano[3,2-*b*]pyran-3-carbonitrile (6h)**

Brown solid; m.p= 135-137 °C, FT-IR (KBr): υ (cm^-1^)= 3313, 2927, 2193, 1643, 1442, 1261, 1021. ^1^H NMR (400 MHz, DMSO-*d*_6_) *δ_ppm_*: 8.26 (s, 1H), 7.33 (t, *J* = 7.9 Hz, 1H), 7.28 – 7.21 (m, 4H), 7.18 (d, *J* = 7.9 Hz, 3H), 7.02 (dd, *J* = 8.2, 2.6 Hz, 1H), 6.91 (t, *J* = 2.0 Hz, 1H), 6.89 – 6.84 (m, 1H), 6.34 (s, 1H), 5.55 (s, 2H), 5.13 (s, 2H), 4.76 (s, 1H), 4.25 – 4.10 (m, 2H), 2.28 (s, 3H). ^13^C NMR (100 MHz, DMSO-*d*_6_) *δ_ppm_*: 169.5, 168.2, 166.9, 162.3, 159.2, 158.3, 148.8, 142.8, 142.4, 137.5, 136.4, 132.9, 131.6, 130.1, 129.3, 128.6, 128.0, 124.5, 120.1, 119.2, 114.5, 113.6, 111.4, 61.1, 59.1, 52.6, 40.2, 20.7. Anal. calcd. for C_27_H_23_N_5_O_5_: C, 65.18; H, 4.66; N, 14.08; Found: C: 65.21; H: 4.70; N: 14.08.

**2-Amino-6-(hydroxymethyl)-4-(3-((1-(4-methoxybenzyl)-1*H*-1,2,3-triazol-4-yl)methoxy)phenyl)-8-oxo-4,8-dihydropyrano[3,2-*b*]pyran-3-carbonitrile (6i)**

Brown solid; m.p= 143-145 °C, FT-IR (KBr): υ (cm^-1^)= 3425, 2928, 2193, 1642, 1513, 1252, 1139. ^1^H NMR (400 MHz, DMSO-*d*_6_) *δ_ppm_*: 8.23 (s, 1H), 7.32 (d, *J* = 8.6 Hz, 2H), 7.22 (d, *J* = 4.0 Hz, 3H), 7.14 (d, *J* = 8.3 Hz, 1H), 6.93 (d, *J* = 8.6 Hz, 2H), 6.87 (d, *J* = 1.7 Hz, 1H), 6.77 (dd, *J* = 8.3, 1.7 Hz, 1H), 6.33 (s, 1H), 5.53 (s, 2H), 5.08 (s, 2H), 4.74 (s, 1H), 4.12-4.26 (m, 2H), 3.74 (s, 3H). ^13^C NMR (100 MHz, DMSO-*d*_6_) *δ_ppm_*: 169.6, 168.1, 159.2, 159.1, 149.1, 149.0, 147.1, 142.8, 136.1, 133.6, 129.6, 127.9, 124.4, 119.6, 119.3, 115.7, 115.5, 114.1, 113.6, 111.5, 111.3, 61.6, 59.1, 55.1, 52.3, 39.8. Anal. calcd. for C_27_H_23_N_5_O_6_: C, 63.15; H, 4.51; N, 13.64; Found: C: 63.15; H: 4.49; N: 13.55.

**2-Amino-4-(3-((1-(4-fluorobenzyl)-1*H*-1,2,3-triazol-4-yl)methoxy)phenyl)-6-(hydroxymethyl)-8-oxo-4,8-dihydropyrano[3,2-*b*]pyran-3-carbonitrile (6j)**

Yellow solid; m.p= 151-153 °C, FT-IR (KBr): υ (cm^-1^)= 3387, 2938, 2192, 1641, 1511, 1420, 1223. ^1^H NMR (400 MHz, DMSO-*d*_6_) *δ_ppm_*: 8.30 (s, 1H), 7.62 – 7.56 (m, 1H), 7.42 (dd, *J* = 8.3, 5.8 Hz, 2H), 7.24 – 7.19 (m, 3H), 7.15 (d, *J* = 8.5 Hz, 1H), 6.89 (d, *J* = 1.9 Hz, 1H), 6.81 – 6.75 (m, 1H), 6.34 (s, 1H), 5.62 (s, 2H), 5.10 (s, 2H), 4.75 (s, 1H), 4.13-4.26 (m, 2H). ^13^C NMR (100 MHz, DMSO-*d*_6_) *δ_ppm_*: 169.6, 168.1, 159.2, 149.1, 147.0, 142.9, 136.1, 135.0, 133.6, 132.2, 130.4, 130.3, 124.7, 119.6, 115.9, 115.7, 115.5, 113.6, 111.3, 61.6, 59.1, 55.4, 45.6. Anal. calcd. for C_26_H_20_FN_5_O_5_: C, 62.27; H, 4.02; N, 13.97; Found: C: 62.14; H: 3.99; N: 13.98.

**2-Amino-4-(4-((1-benzyl-1*H*-1,2,3-triazol-4-yl)methoxy)-3-methoxyphenyl)-6-(hydroxymethyl)-8-oxo-4,8-dihydropyrano[3,2-*b*]pyran-3-carbonitrile (6k)**

Yellow solid; m.p= 159-161 °C, FT-IR (KBr): υ (cm^-1^)= 3400, 2925, 2193, 1644, 1509, 1409, 1210. ^1^H NMR (400 MHz, DMSO-*d*_6_) *δ_ppm_*: 8.28 (s, 1H), 7.41 – 7.36 (m, 5H), 7.21 (s, 2H), 7.14 (d, *J* = 8.5 Hz, 1H), 6.88 (d, *J* = 2.1 Hz, 1H), 6.77 (dd, *J* = 8.5, 2.1 Hz, 1H), 6.33 (s, 1H), 5.60 (s, 2H), 5.11 (s, 2H), 4.74 (s, 1H), 4.13-415 (m, 2H), 3.73 (s, 3H). ^13^C NMR (100 MHz, DMSO-*d*_6_) *δ_ppm_*: 160.5, 159.0, 151.0, 148.6, 146.3, 138.3, 137.7, 137.5, 133.2, 131.1, 130.7, 128.1, 127.8, 127.0, 126.7, 121.9, 121.7, 119.0, 115.3, 113.7, 112.0, 111.7, 109.7, 61.7, 57.7, 55.4, 52.0, 36.1. Anal. calcd. for C_27_H_23_N_5_O_6_: C, 63.15; H, 4.51; N, 13.64; Found: C: 63.16; H: 4.46; N: 13.66.

**2-Amino-4-(4-((1-(4-chlorobenzyl)-1*H*-1,2,3-triazol-4-yl)methoxy)-3-methoxyphenyl)-6-(hydroxymethyl)-8-oxo-4,8-dihydropyrano[3,2-*b*]pyran-3-carbonitrile (6l)**

Brown solid; m.p = 190-192 °C, FT-IR (KBr): υ (cm^-1^)= 3430, 2927, 2193, 1642, 1512, 1092. ^1^H NMR (400 MHz, DMSO-*d*_6_) *δ_ppm_*: 8.29 (s, 1H), 7.46 (d, *J* = 8.4 Hz, 2H), 7.37 (d, *J* = 8.4 Hz, 2H), 7.21 (s, 2H), 7.15 (d, *J* = 8.4 Hz, 1H), 6.88 (d, *J* = 2.0 Hz, 1H), 6.78 (dd, *J* = 8.4, 2.0 Hz, 1H), 6.33 (s, 1H), 5.63 (s, 2H), 5.11 (s, 2H), 4.75 (s, 1H), 4.13-4.25 (m, 2H), 3.73 (s, 3H). ^13^C NMR (100 MHz, DMSO) δ 168.5, 167.2, 162.1, 159.6, 158.2, 157.3, 147.8, 141.9, 141.4, 135.4, 131.2, 129.3, 129.3, 129.1, 123.6, 119.2, 118.2, 114.7, 114.5, 113.5, 112.6, 110.4, 60.1, 58.1, 54.5, 51.0, 44.7. Anal. calcd. for C_27_H_22_ClN_5_O_6_: C, 59.18; H, 4.05; N, 12.78; Found: C: 59.20; H: 4.01; N: 12.70.

**2-Amino-6-(hydroxymethyl)-4-(3-methoxy-4-((1-(4-methylbenzyl)-1*H*-1,2,3-triazol-4-yl)methoxy)phenyl)-8-oxo-4,8-dihydropyrano[3,2-*b*]pyran-3-carbonitrile (6m)**

Brown solid; m.p= 165-167 °C, FT-IR (KBr): υ (cm^-1^)= 3405, 2189, 1642, 1512, 1420, 1215. ^1^H NMR (400 MHz, DMSO-*d*_6_) *δ_ppm_*: 8.24 (s, 1H), 7.19-7.25 (m, 6H), 7.18 – 7.10 (m, 2H), 6.88 (d, *J* = 2.1 Hz, 1H), 6.77 (dd, *J* = 8.3, 2.2 Hz, 1H), 6.33 (s, 1H), 5.56 (s, 2H), 5.09 (s, 2H), 4.74 (s, 1H), 4.21 – 4.08 (m, 2H), 3.72 (s, 3H), 2.28 (s, 3H). ^13^C NMR (100 MHz, DMSO-*d*_6_) *δ_ppm_*: 169.6, 168.2, 159.2, 149.1, 149.0, 147.0, 142.8, 137.5, 136.1, 133.6, 133.0, 129.3, 128.0, 124.6, 119.6, 119.3, 113.6, 111.5, 111.3, 61.6, 59.1, 55.4, 52.6, 39.8, 20.7. Anal. calcd. for C_28_H_25_N_5_O_6_: C, 63.75; H, 4.78; N, 13.28; Found: C: 63.72; H: 4.78; N: 13.32.

**2-Amino-6-(hydroxymethyl)-4-(3-methoxy-4-((1-(4-methoxybenzyl)-1*H*-1,2,3-triazol-4-yl)methoxy)phenyl)-8-oxo-4,8-dihydropyrano[3,2-*b*]pyran-3-carbonitrile (6n)**

Brown solid; m.p= 163-165 °C, FT-IR (KBr): υ (cm^-1^)= 3430, 2924, 2193, 1641, 1514. ^1^H NMR (400 MHz, DMSO-*d*_6_) *δ_ppm_*: 8.26 (s, 1H), 7.31 (d, *J* = 8.4 Hz, 2H), 7.24 (s, 2H), 7.01 (d, *J* = 8.0, Hz, 1H), 6.93 (d, *J* = 8.4 Hz, 2H), 6.92 – 6.89 (m, 1H), 6.87 (d, *J* = 8.0 Hz, 1H), 6.34 (s, 1H), 5.72 (t, *J* = 5.2 Hz, 1H), 5.52 (s, 2H), 5.12 (s, 2H), 4.76 (s, 1H), 4.18 (qd, *J* = 15.9, 5.9 Hz, 2H), 3.73 (s, 3H), 3.60 (s, 3H). ^13^C NMR (101 MHz, DMSO) δ 169.5, 168.2, 159.2, 159.1, 158.3, 148.8, 142.4, 136.4, 130.1, 129.6, 127.9, 124.3, 120.1, 119.2, 114.5, 114.1, 113.6, 111.4, 69.7, 61.1, 59.1, 55.1, 52.4, 40.2. Anal. calcd. for C_28_H_25_N_5_O_7_: C, 61.87; H, 4.64; N, 12.89; Found: C: 61.85; H: 4.64; N: 12.67.

**2-Amino-4-(4-((1-(4-fluorobenzyl)-1*H*-1,2,3-triazol-4-yl)methoxy)-3-methoxyphenyl)-6-(hydroxymethyl)-8-oxo-4,8-dihydropyrano[3,2-*b*]pyran-3-carbonitrile (6o)**

Brown solid; m.p= 181-183 °C, Mp: FT-IR (KBr): υ (cm^-1^)= 3441, 2193, 1637, 1510, 1223. ^1^H NMR (400 MHz, DMSO-*d*_6_) *δ_ppm_*: 8.26 (s, 1H), 7.44 – 7.40 (m, 2H), 7.35 – 7.31 (m, 2H), 7.14 (s, 2H), 7.11 (d, *J* = 8.4 Hz, 1H), 6.84 (d, *J* = 2.2 Hz, 1H), 6.74 (dd, *J* = 8.4, 2.2 Hz, 1H), 6.29 (s, 1H), 5.59 (s, 2H), 5.07 (s, 2H), 4.71 (s, 1H), 4.09-4.21 (m, 2H), 3.69 (s, 3H). ^13^C NMR (100 MHz, DMSO-*d*_6_) *δ_ppm_*: 169.5, 168.2, 163.1, 160.6, 159.2, 158.3, 148.8, 142.9, 142.4, 136.4, 132.2, 130.3, 130.3, 130.1, 124.6, 120.2, 119.2, 115.7, 115.5, 114.5, 113.6, 111.4, 61.1, 59.1, 55.5, 52.0, 45.7. Anal. calcd. for C_27_H_22_FN_5_O_6_: C, 61.02; H, 4.17; N, 13.18; Found: C: 61.00; H: 4.11; N: 13.15.

# 2-Amino-6-(hydroxymethyl)-8-oxo-4-(4-(prop-2-yn-1-yloxy)phenyl)-4,8-dihydropyrano[3,2-*b*]pyran-3-carbonitrile (4i)


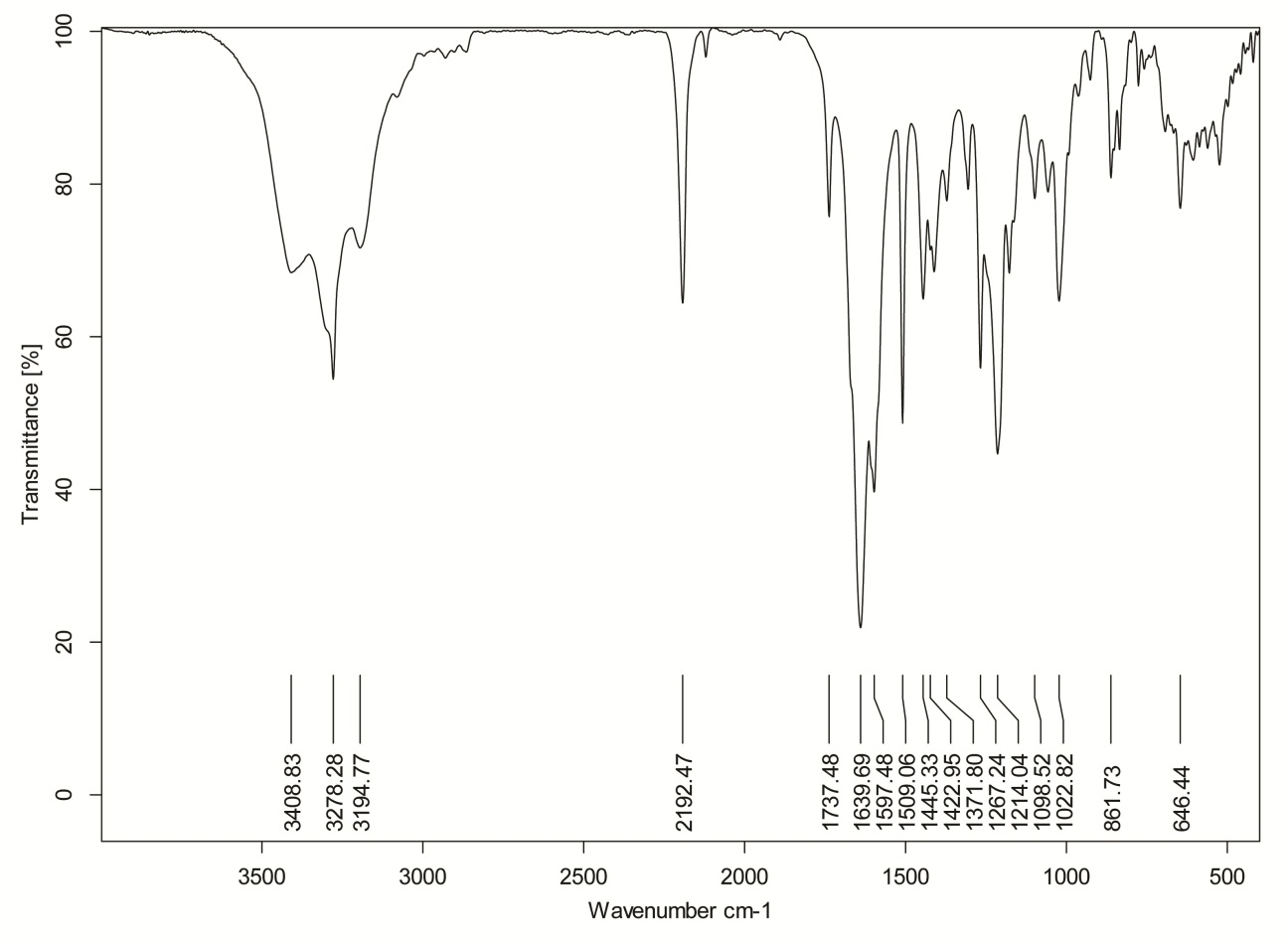


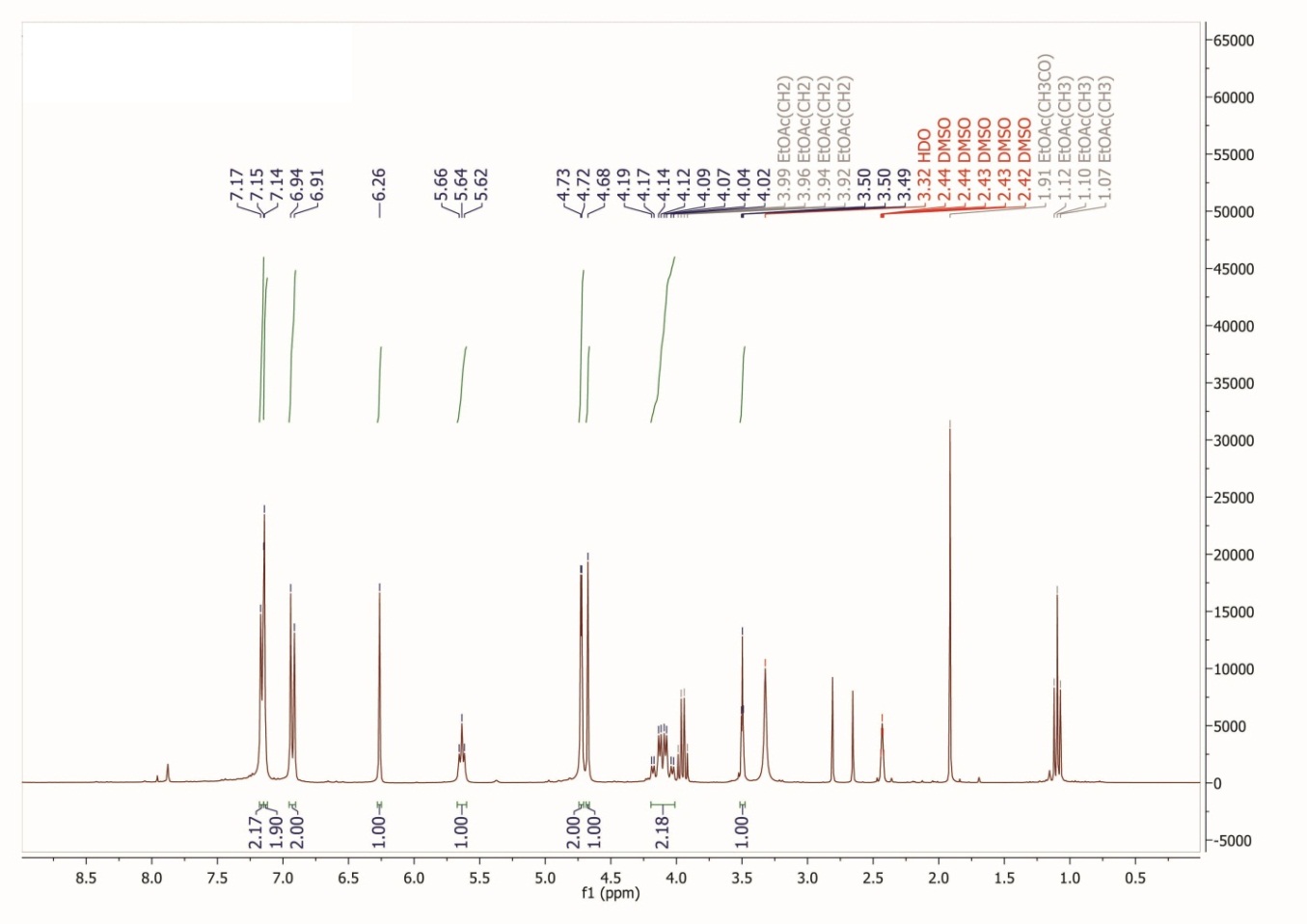

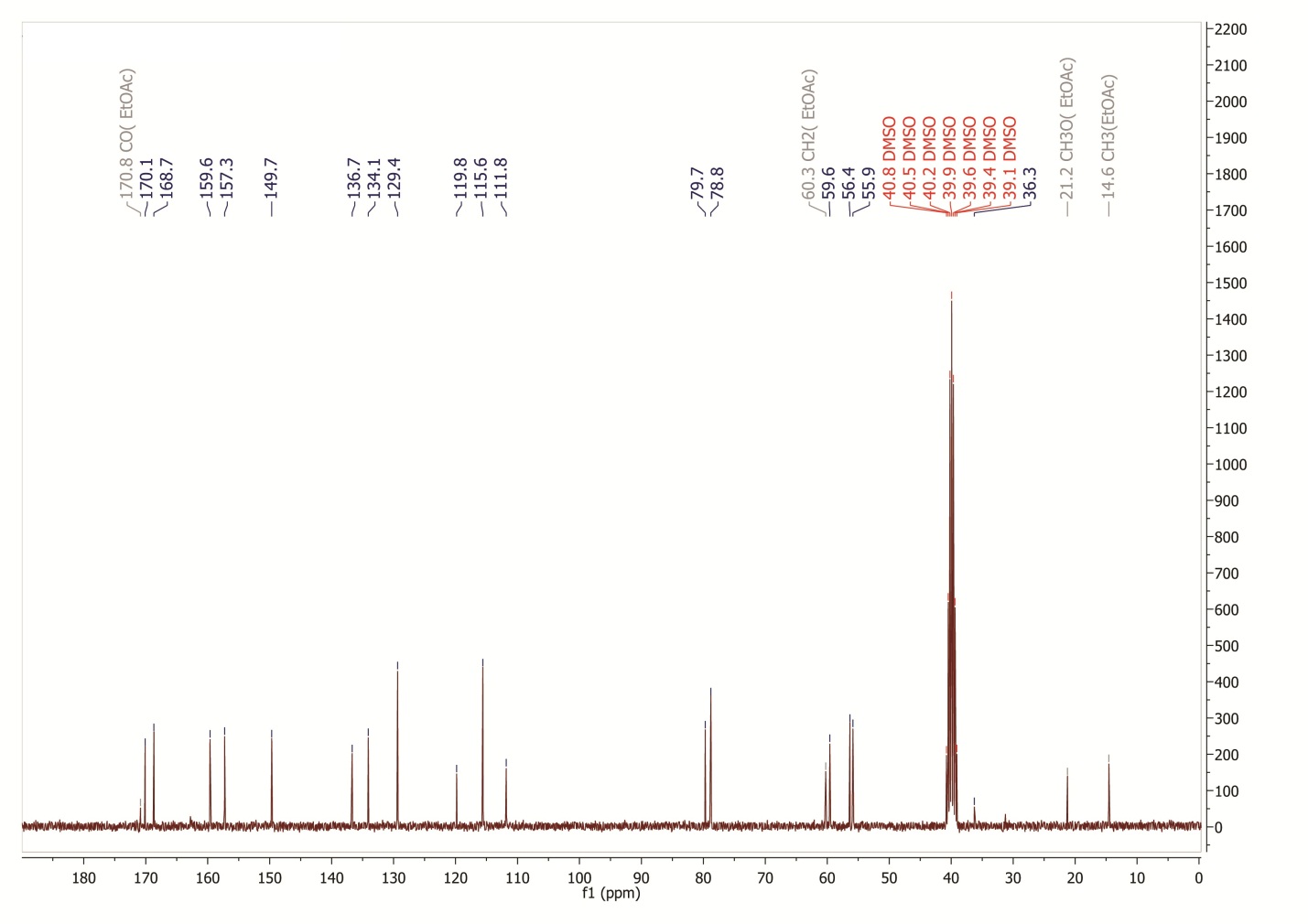

# 2-Amino-6-(hydroxymethyl)-8-oxo-4-(3-(prop-2-yn-1-yloxy)phenyl)-4,8-dihydropyrano[3,2-*b*]pyran-3-carbonitrile (4j)


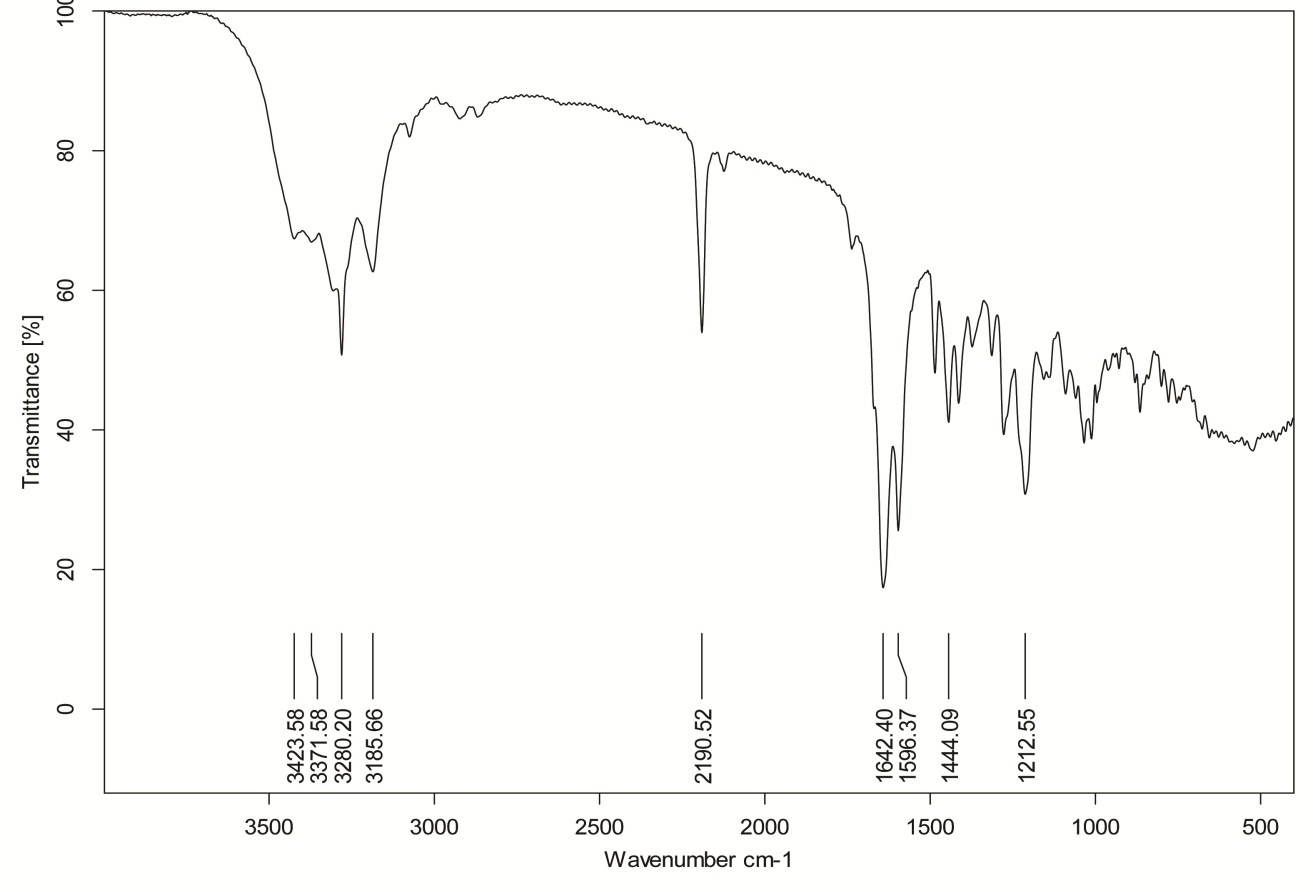


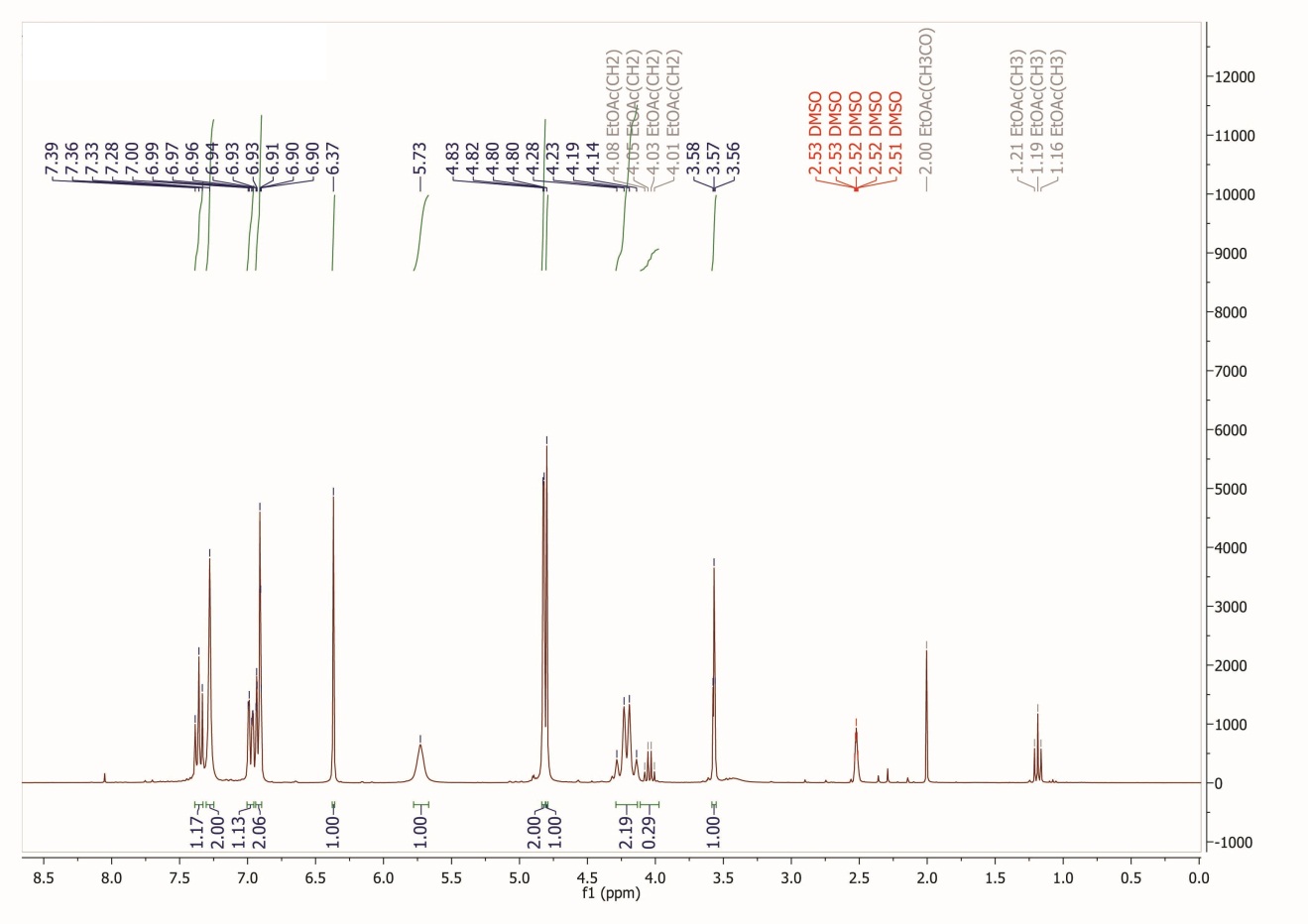

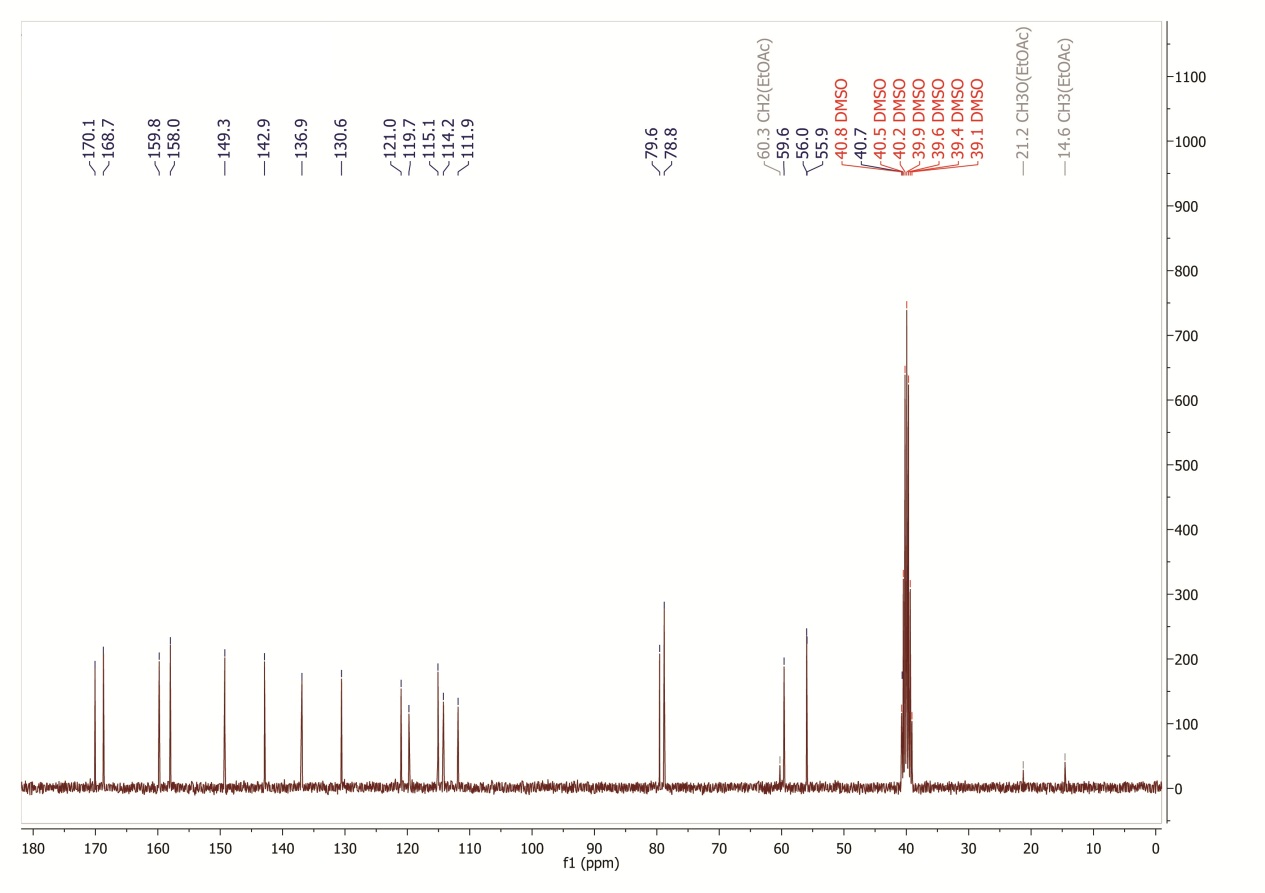

# 2-Amino-6-(hydroxymethyl)-4-(3-methoxy-4-(prop-2-yn-1-yloxy)phenyl)-8-oxo-4,8-dihydropyrano[3,2-*b*]pyran-3-carbonitrile (4k)

**
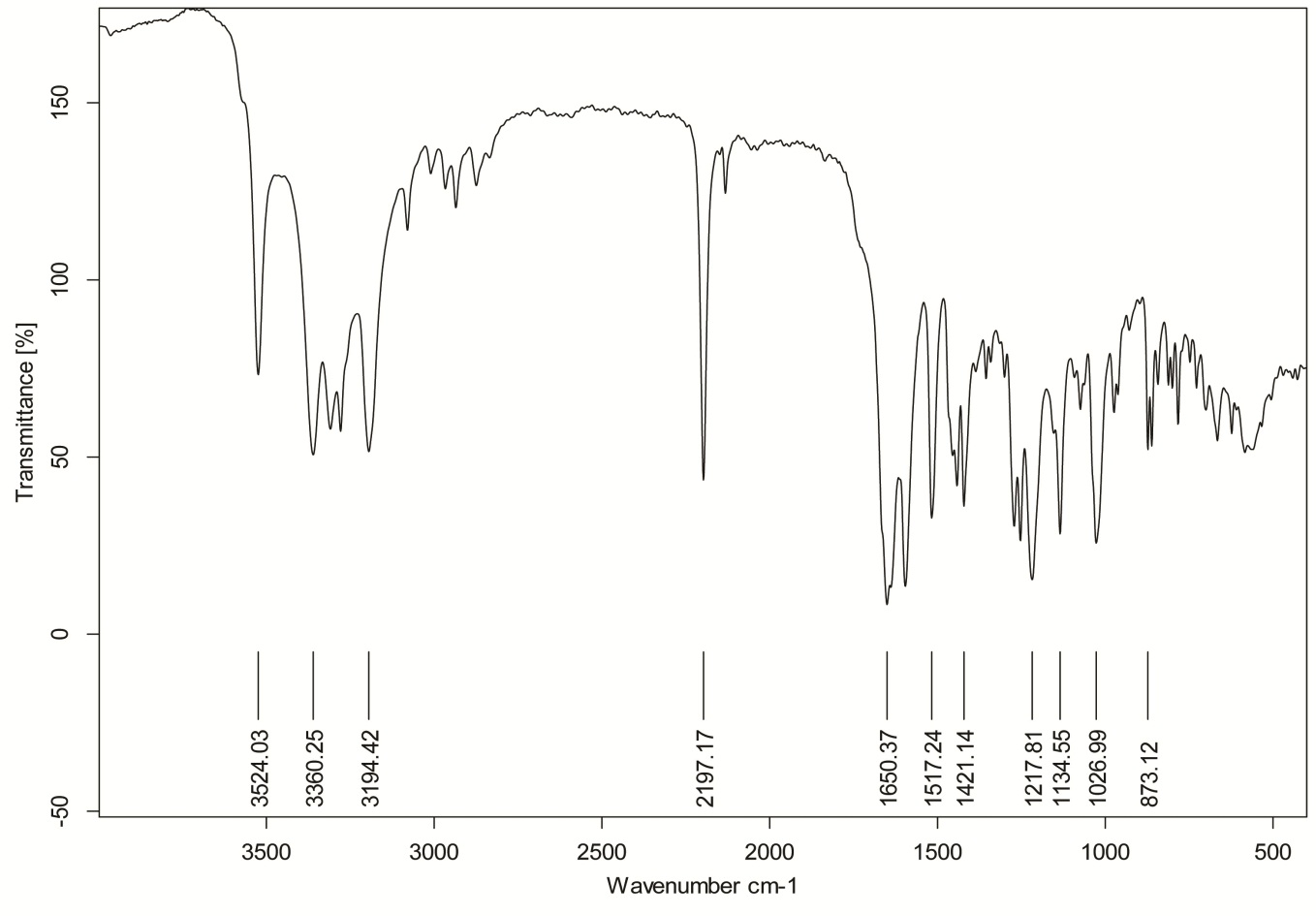
**

**
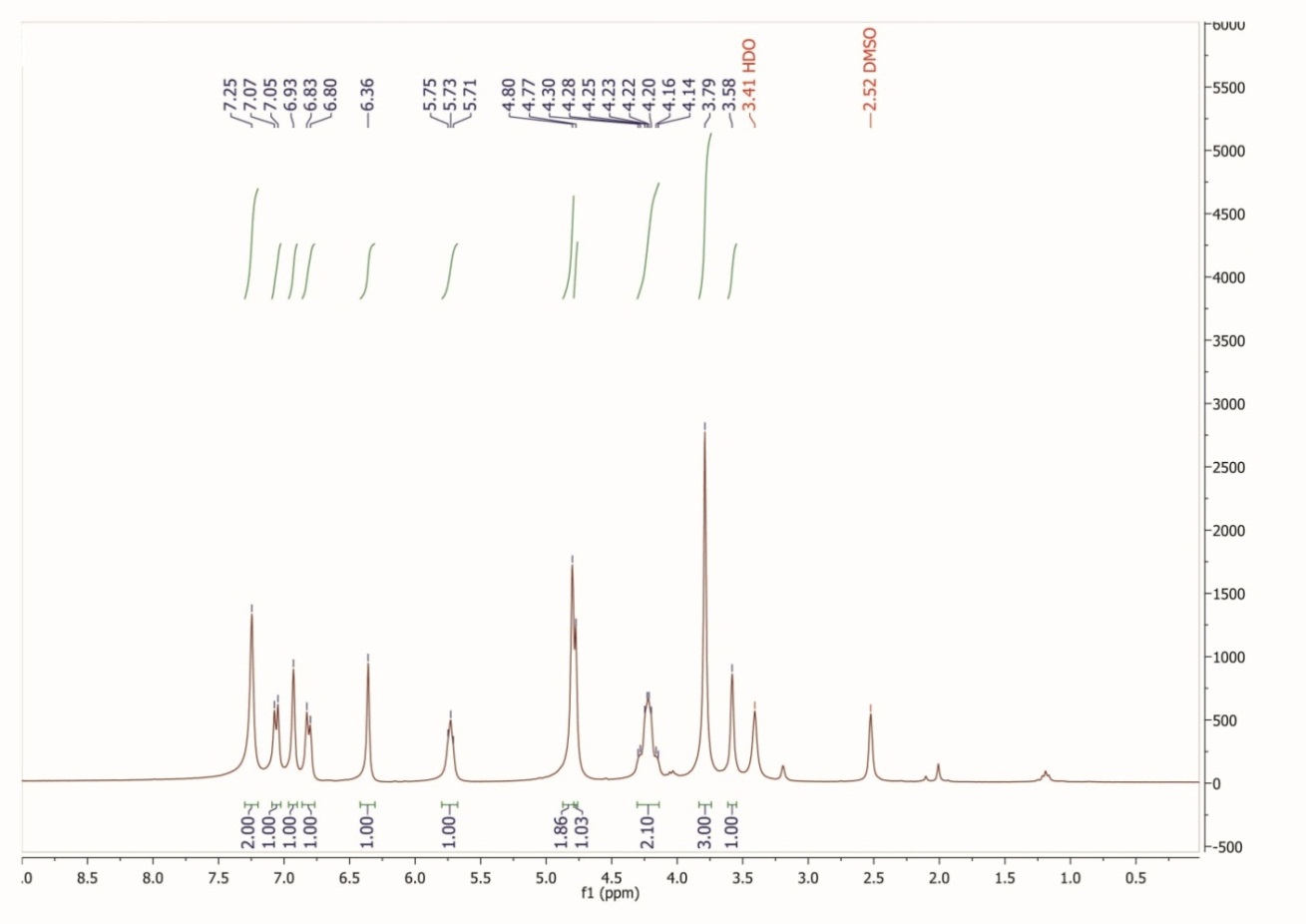

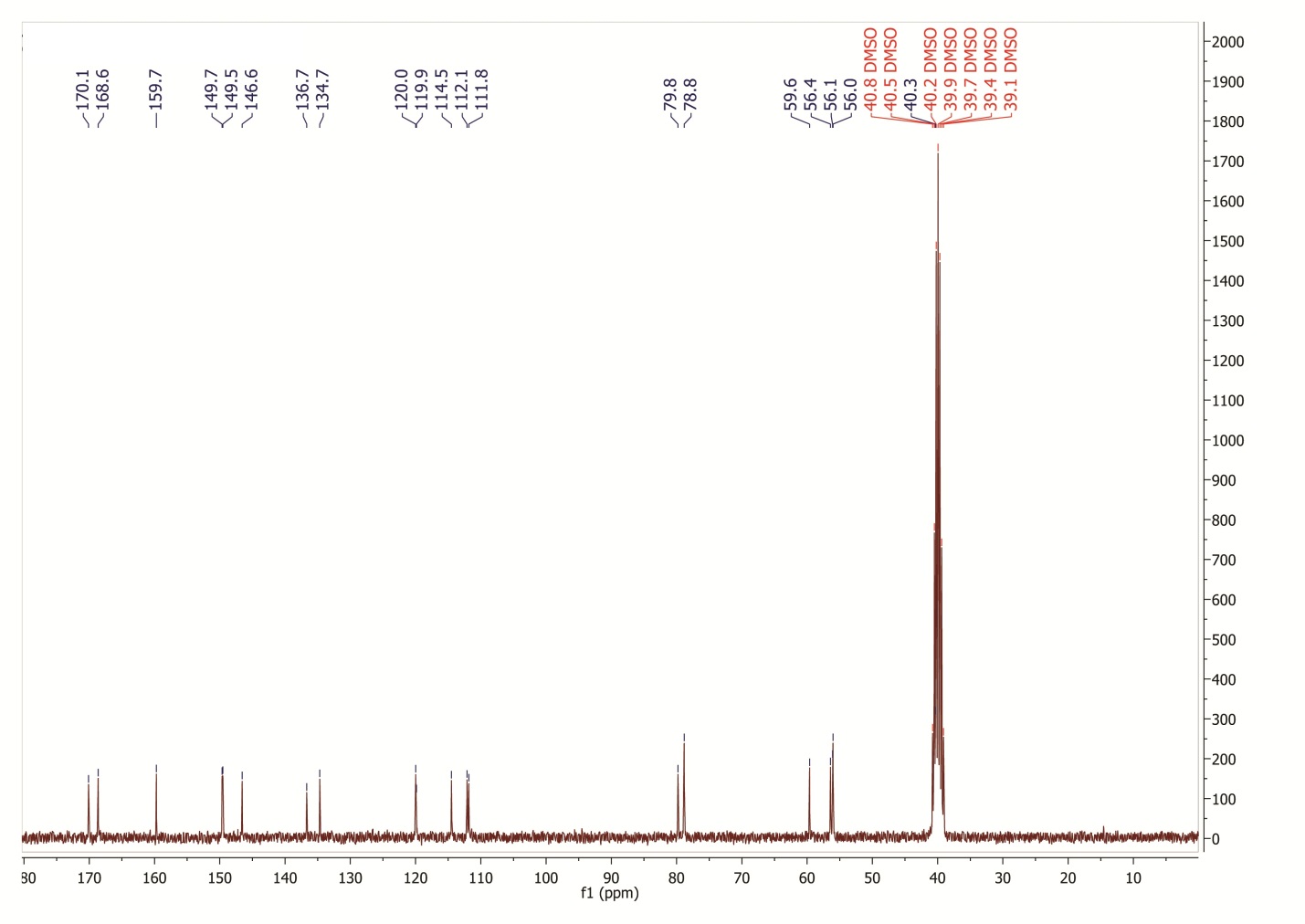
**

# 3-Amino-4-(4-((1-benzyl-1*H*-1,2,3-triazol-4-yl)methoxy)phenyl)-6-(hydroxymethyl)-8-oxo-4,8-dihydropyrano[3,2-*b*]pyran-2-carbonitrile (6a)


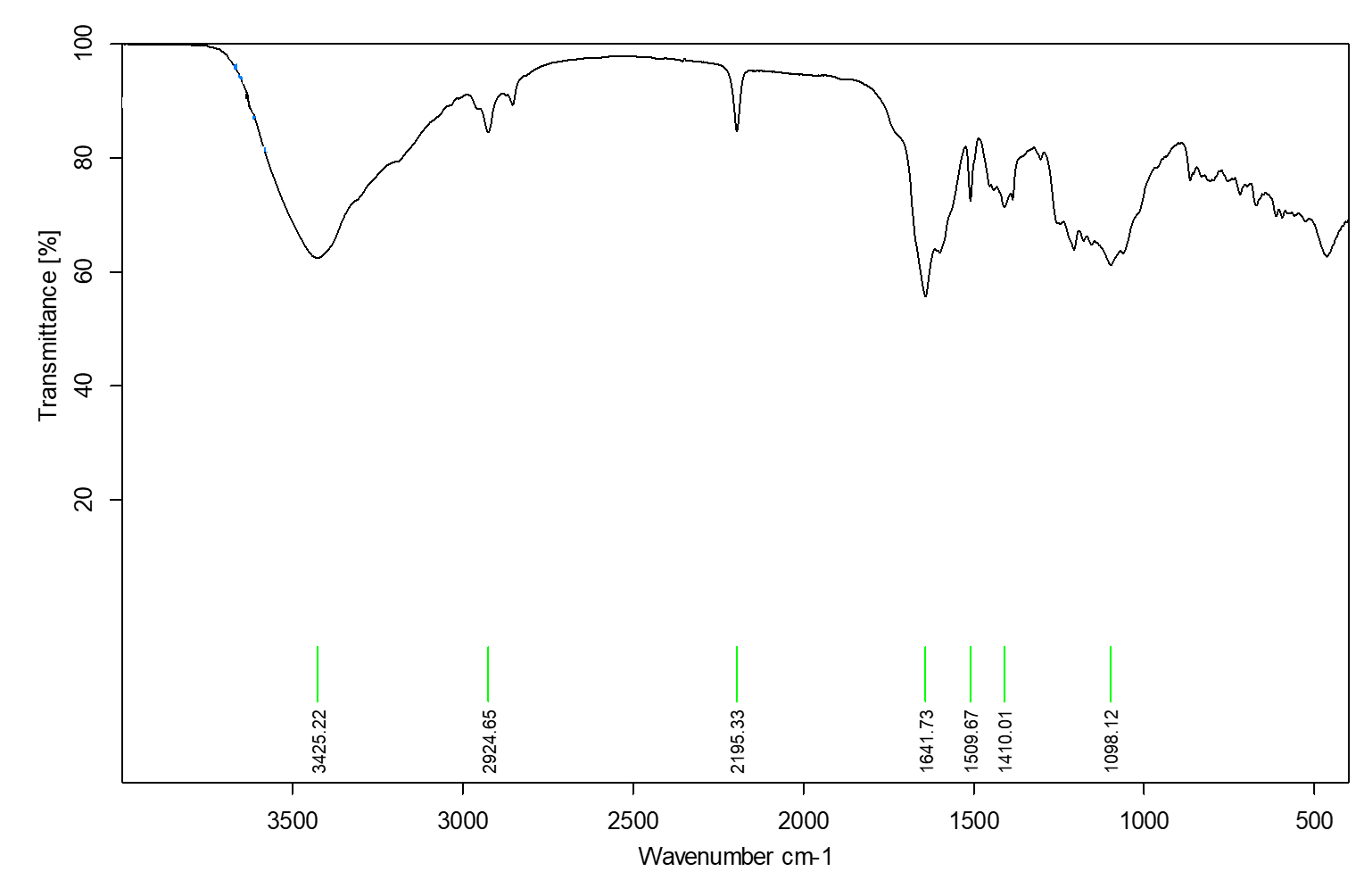


**
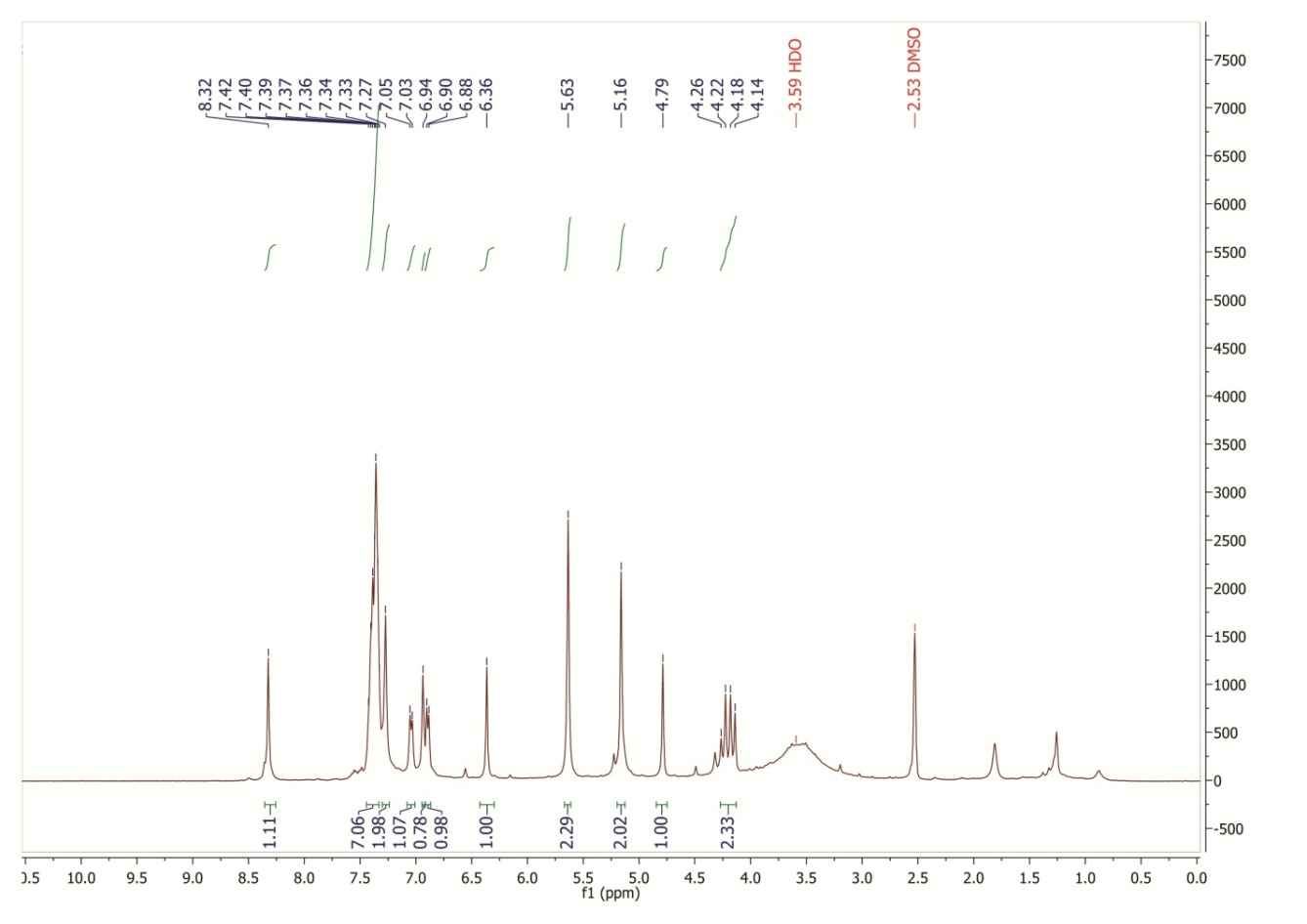

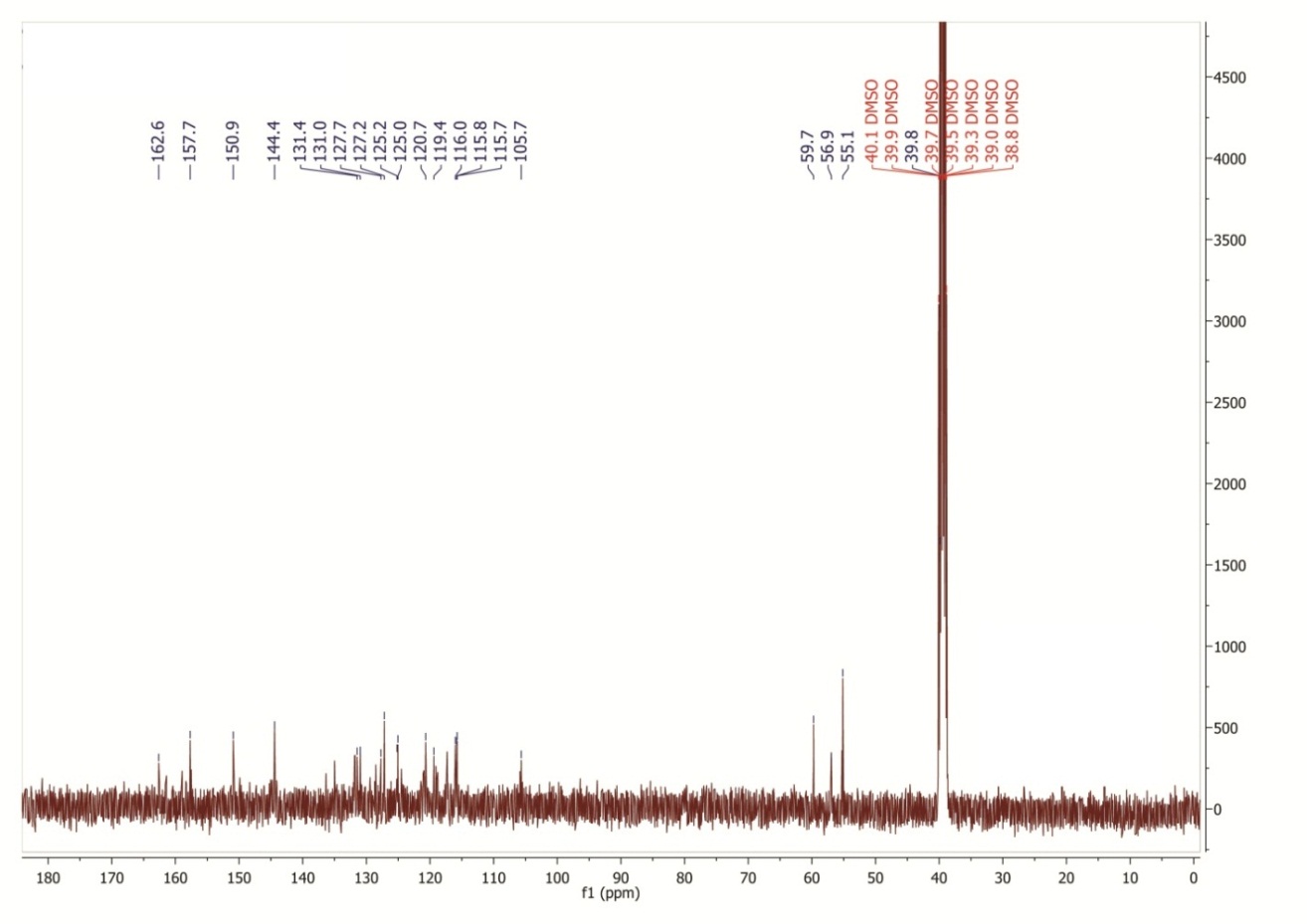
**

-Amino-4-(4-((1-(4-chlorobenzyl)-1*H*-1,2,3-triazol-4-yl)methoxy)phenyl)-6-(hydroxymethyl)-8-oxo-4,8-dihydropyrano[3,2-*b*]pyran-3-carbonitrile (6b)


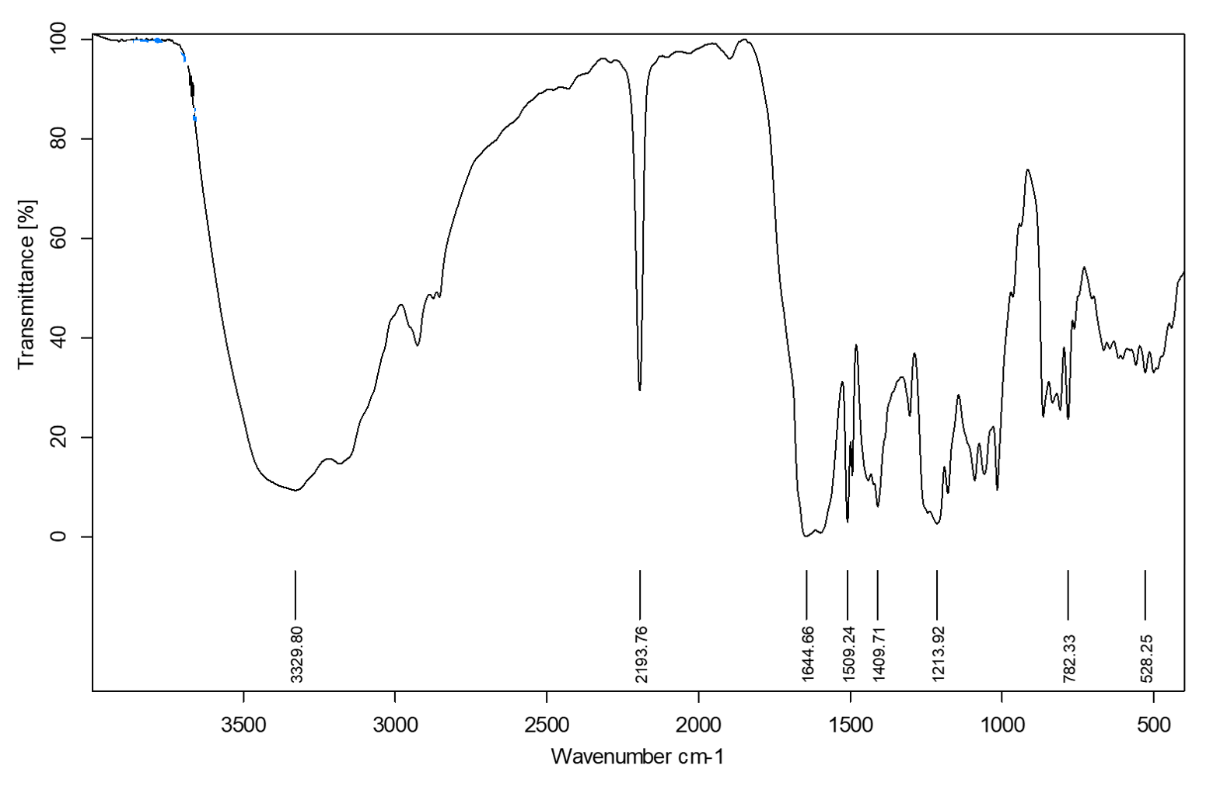


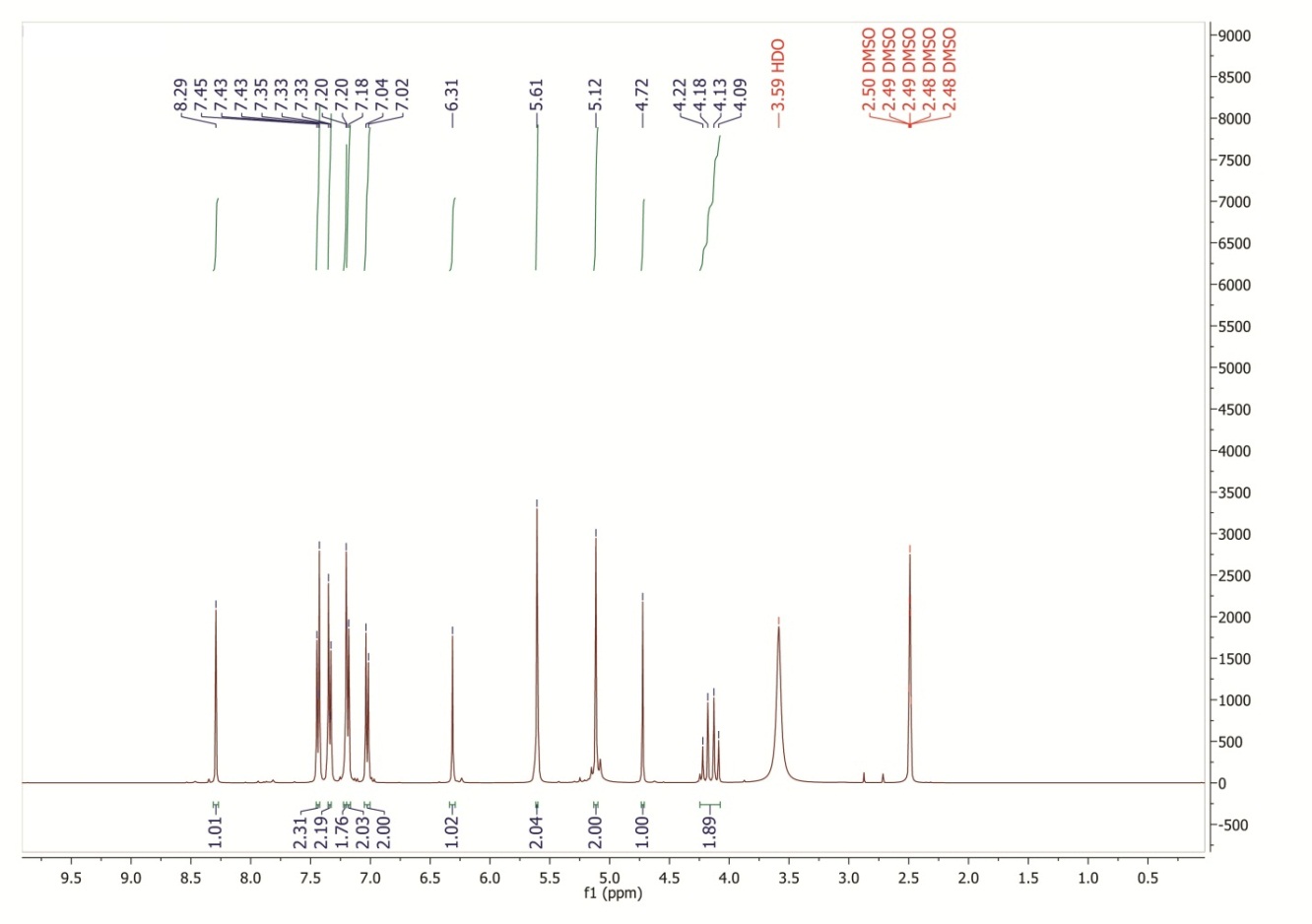


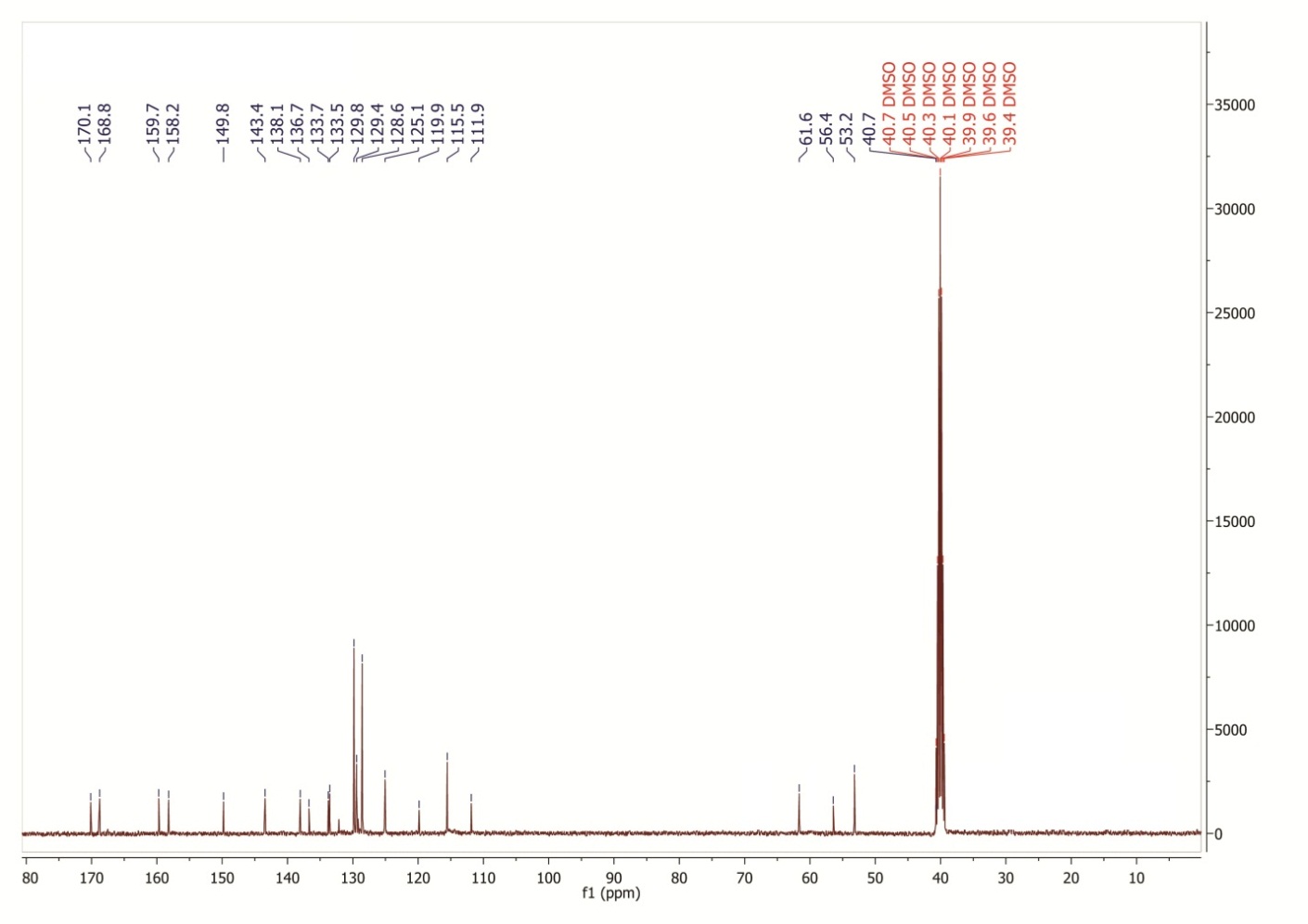

# 2-Amino-6-(hydroxymethyl)-4-(4-((1-(4-methylbenzyl)-1*H*-1,2,3-triazol-4-yl)methoxy)phenyl)-8-oxo-4,8-dihydropyrano[3,2-*b*]pyran-3-carbonitrile (6c)


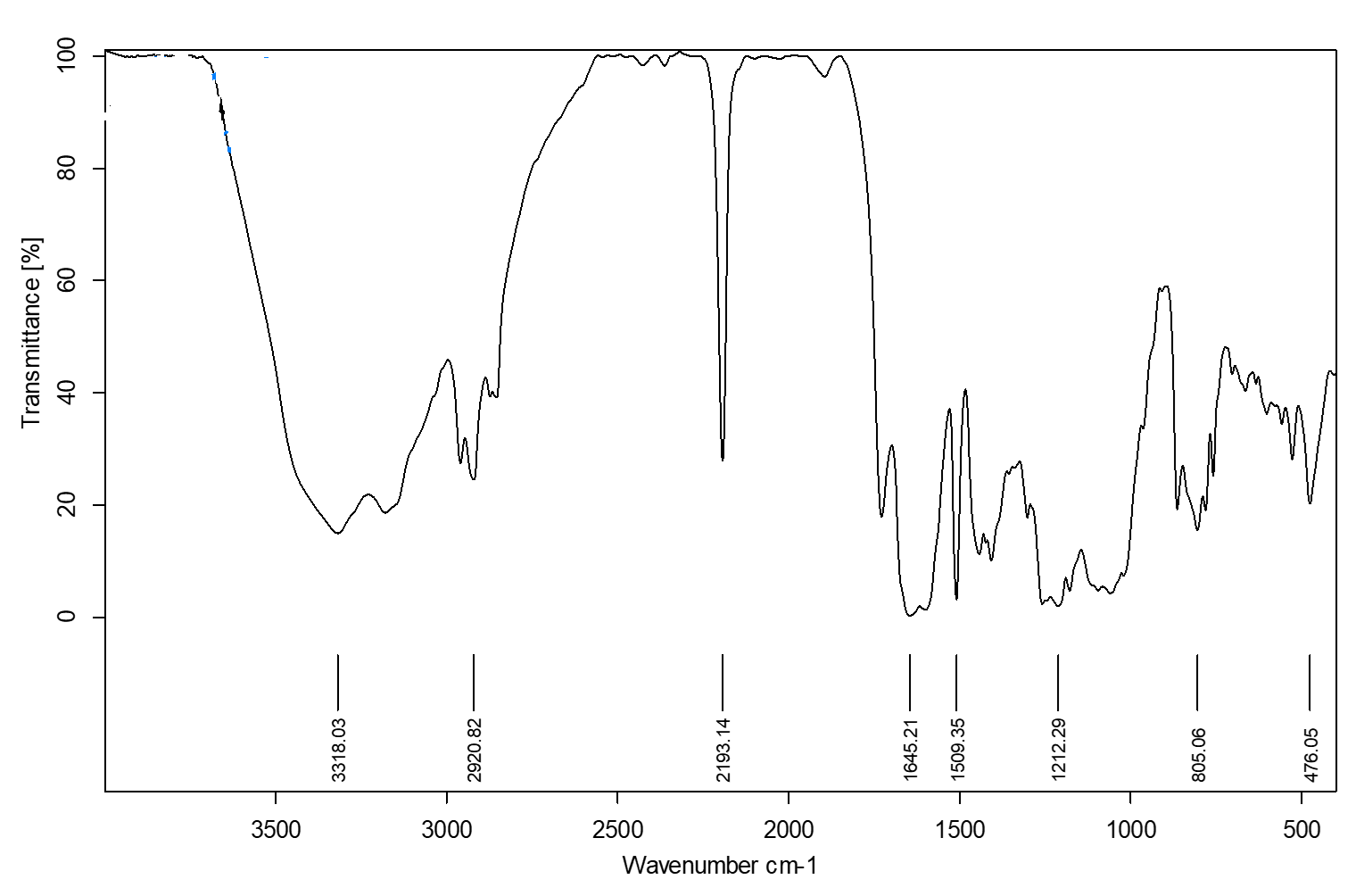


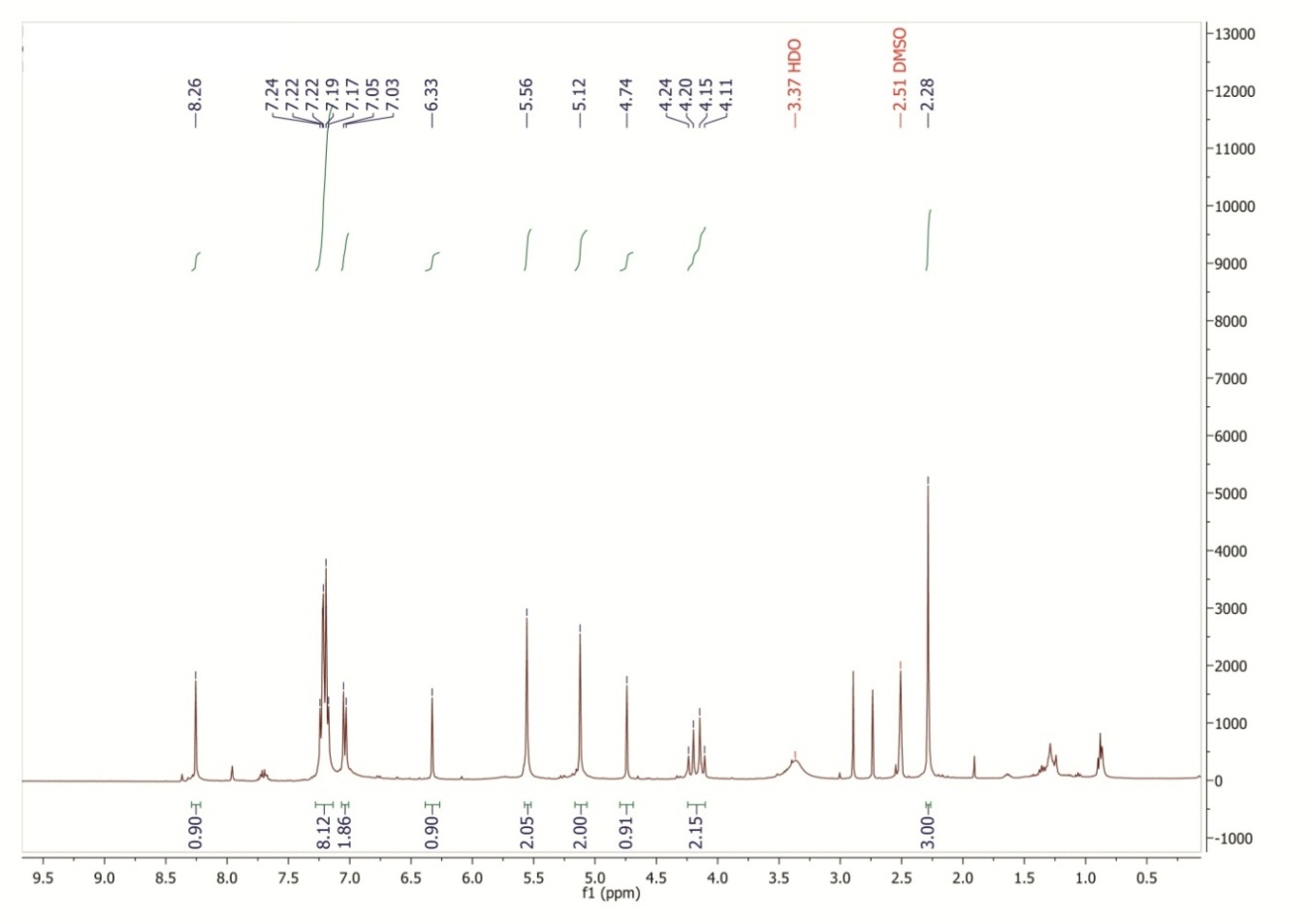

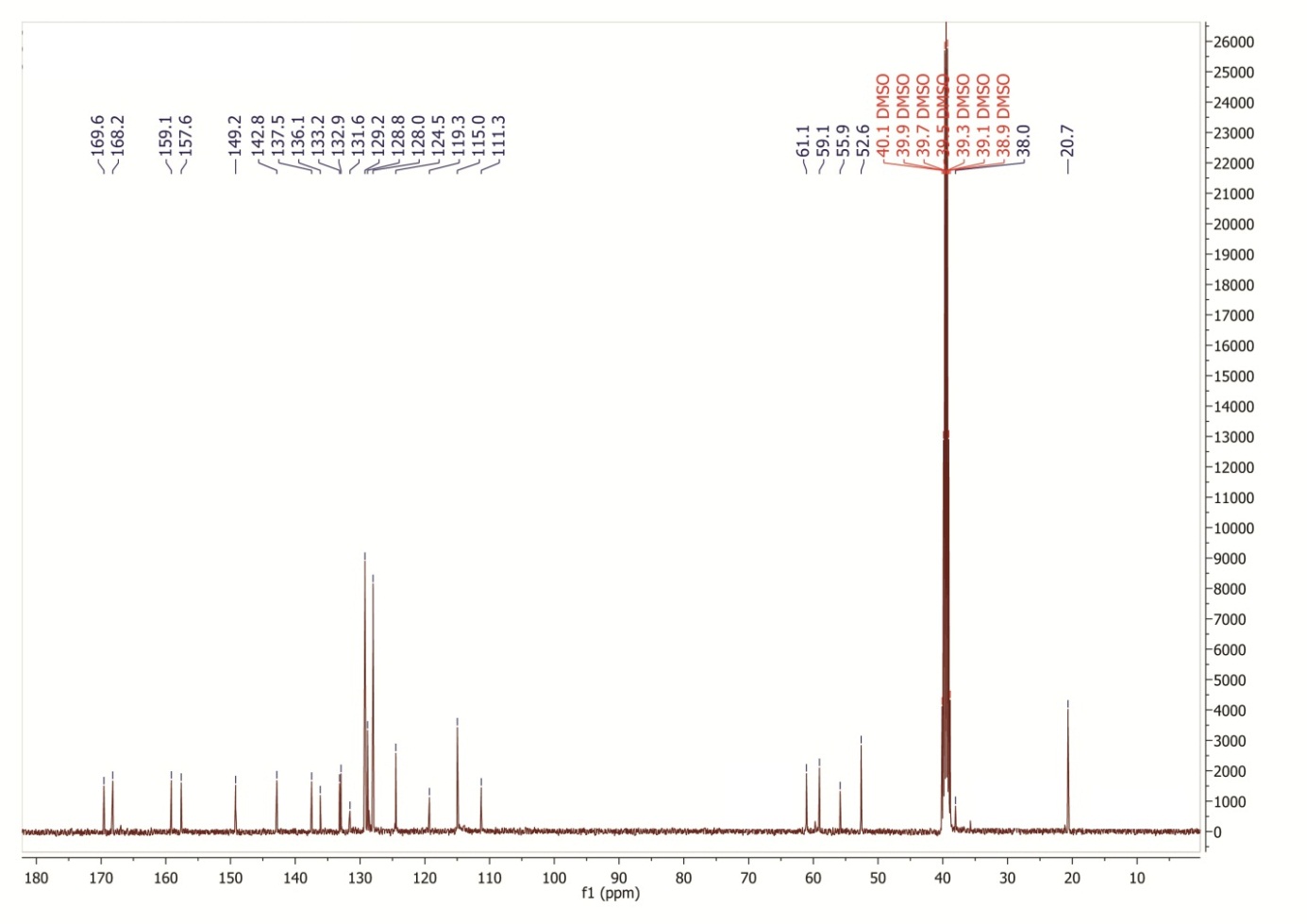

# 2-Amino-6-(hydroxymethyl)-4-(4-((1-(4-methoxybenzyl)-1*H*-1,2,3-triazol-4-yl)methoxy)phenyl)-8-oxo-4,8-dihydropyrano[3,2-*b*]pyran-3-carbonitrile (6d)


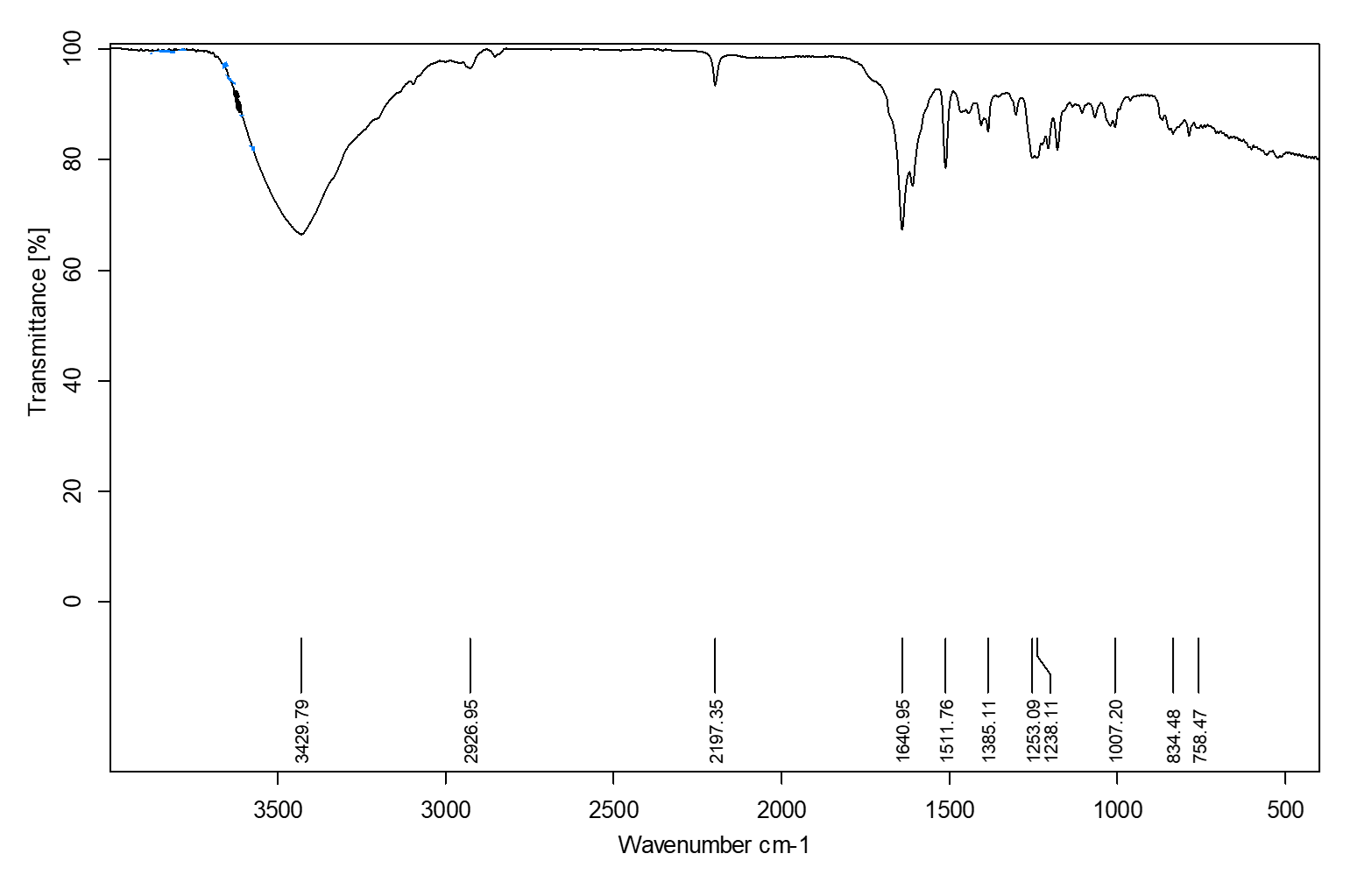


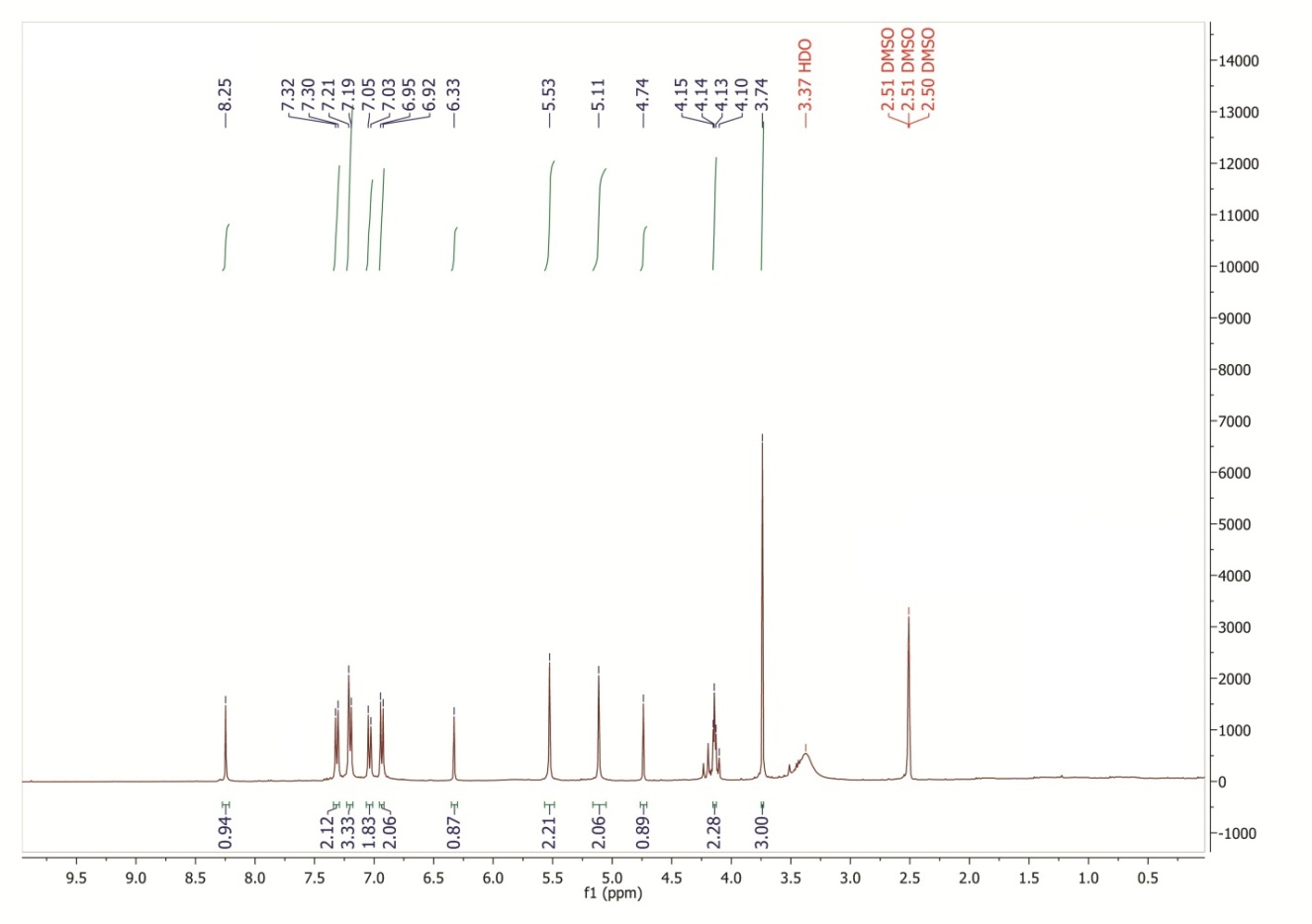


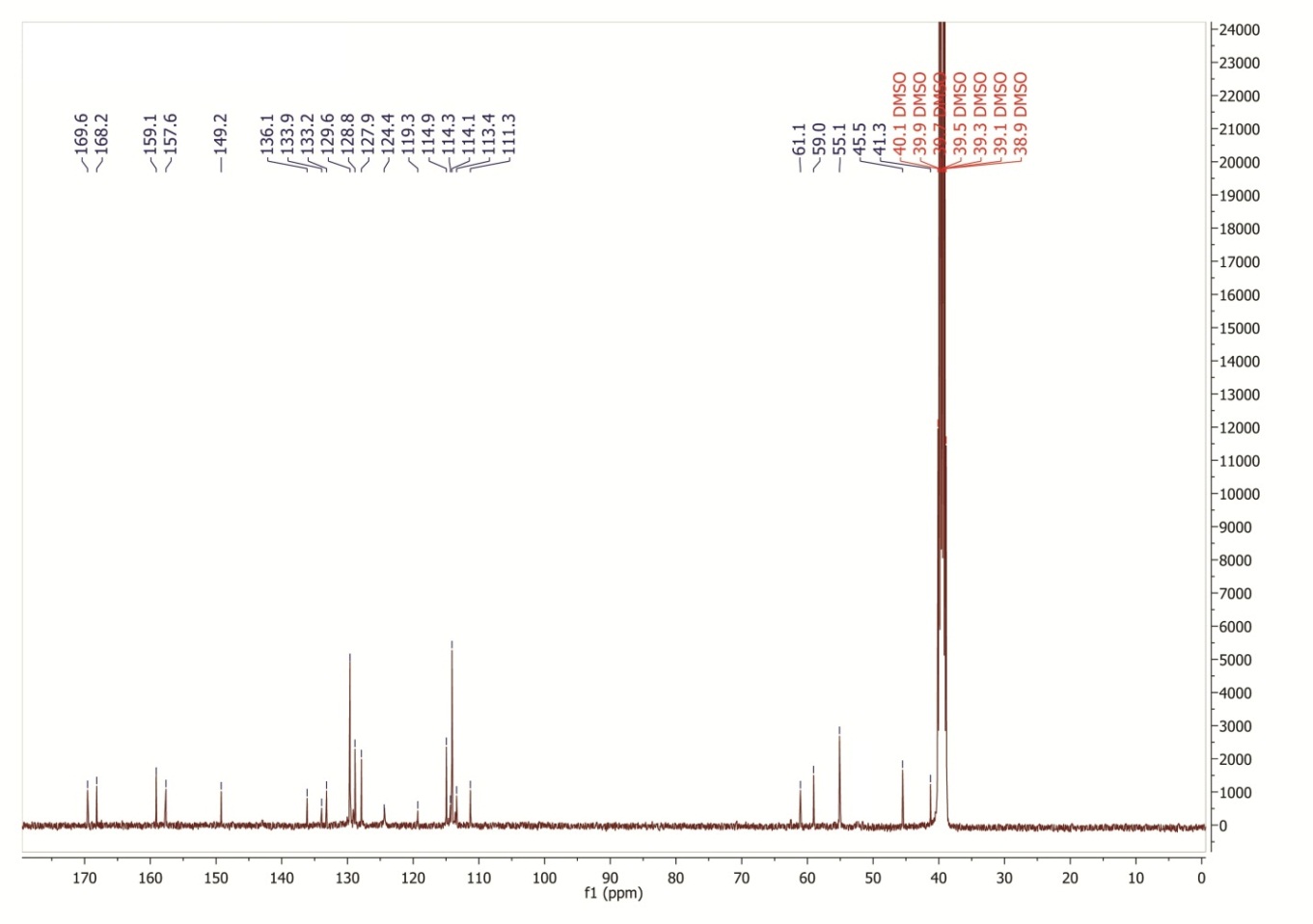

# 2-Amino-4-(4-((1-(4-fluorobenzyl)-1*H*-1,2,3-triazol-4-yl)methoxy)phenyl)-6-(hydroxymethyl)-8-oxo-4,8-dihydropyrano[3,2-*b*]pyran-3-carbonitrile (6e)


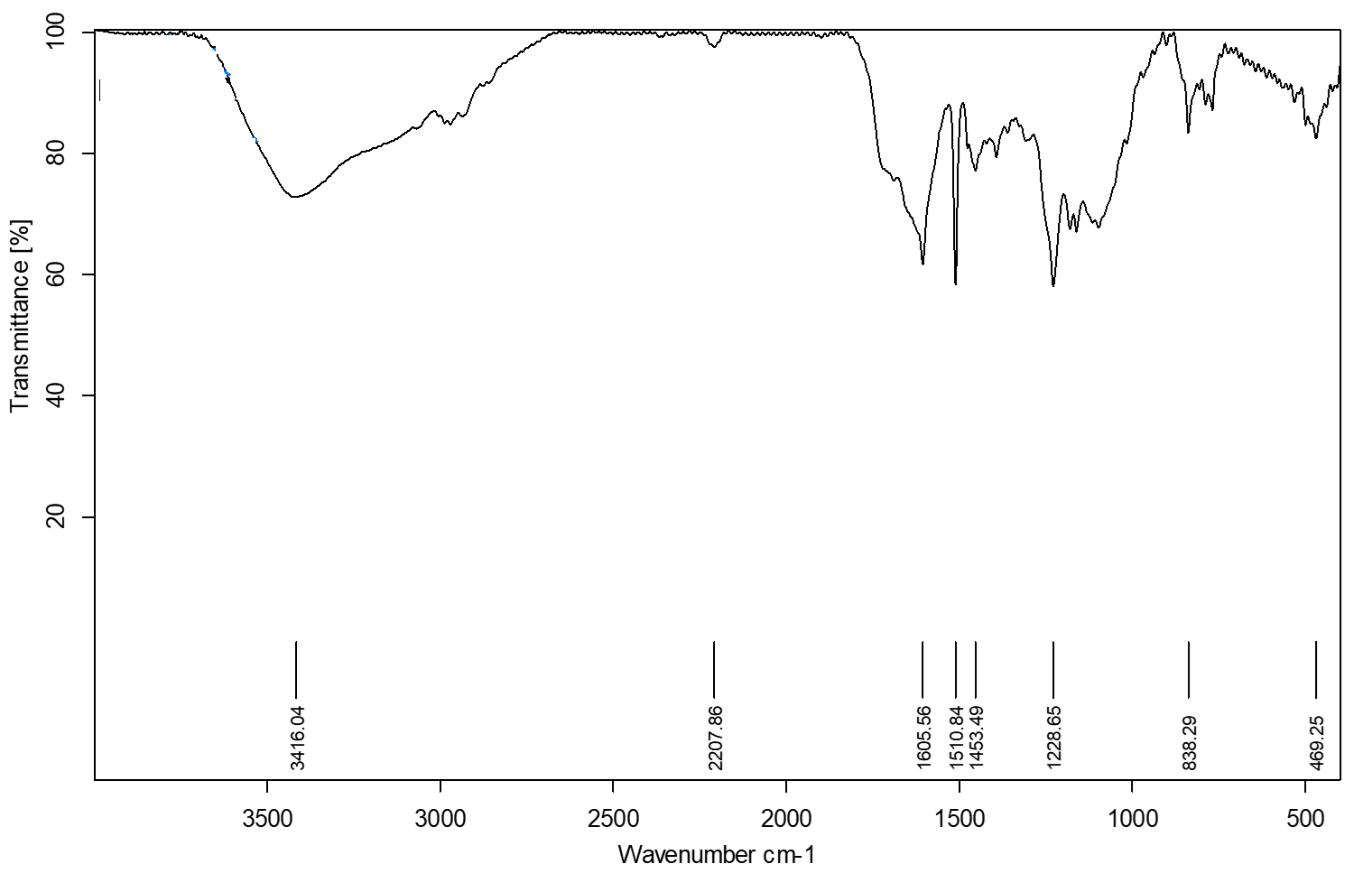


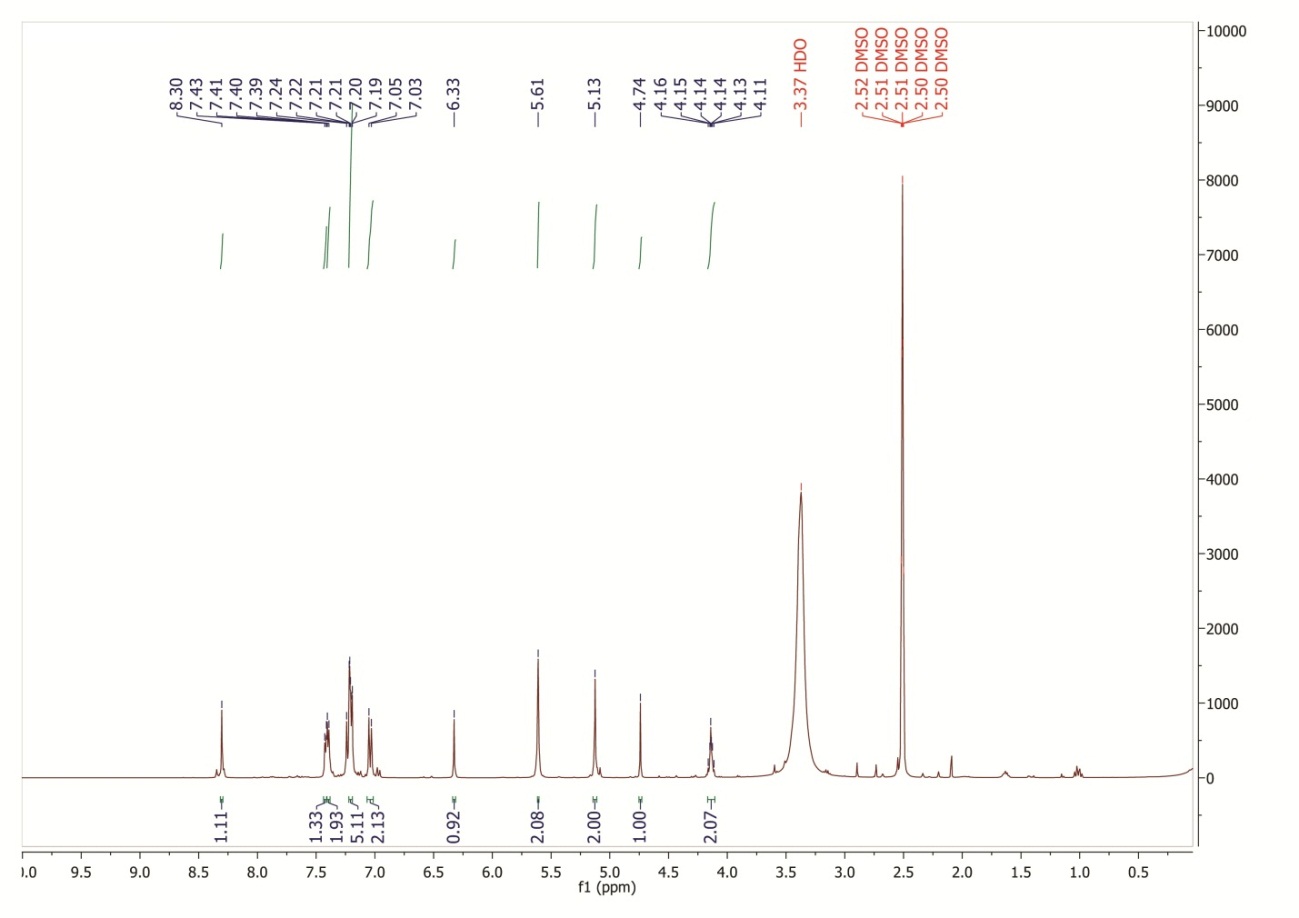


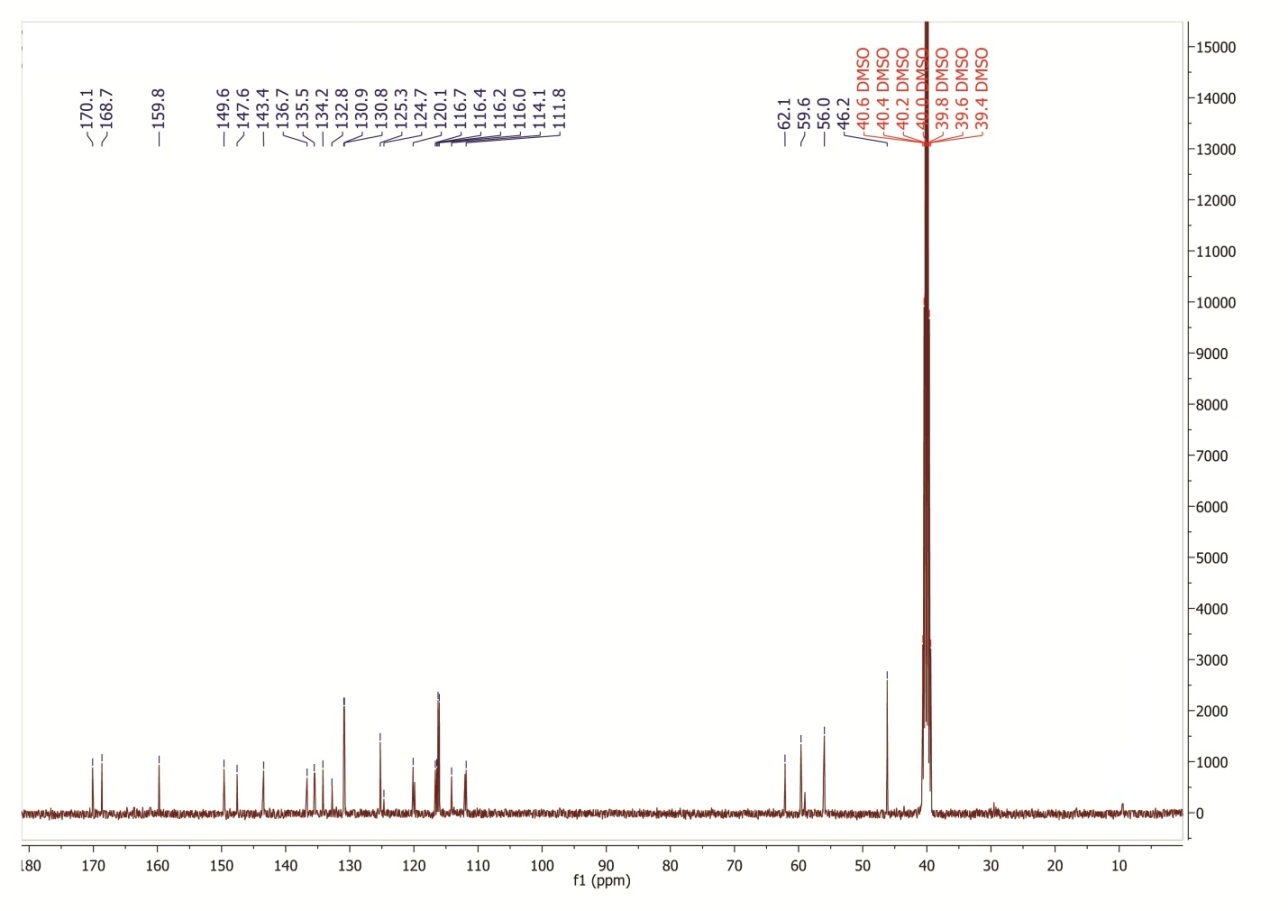

# 2-Amino-4-(3-((1-benzyl-1*H*-1,2,3-triazol-4-yl)methoxy)phenyl)-6-(hydroxymethyl)-8-oxo-4,8-dihydropyrano[3,2-*b*]pyran-3-carbonitrile (6f)


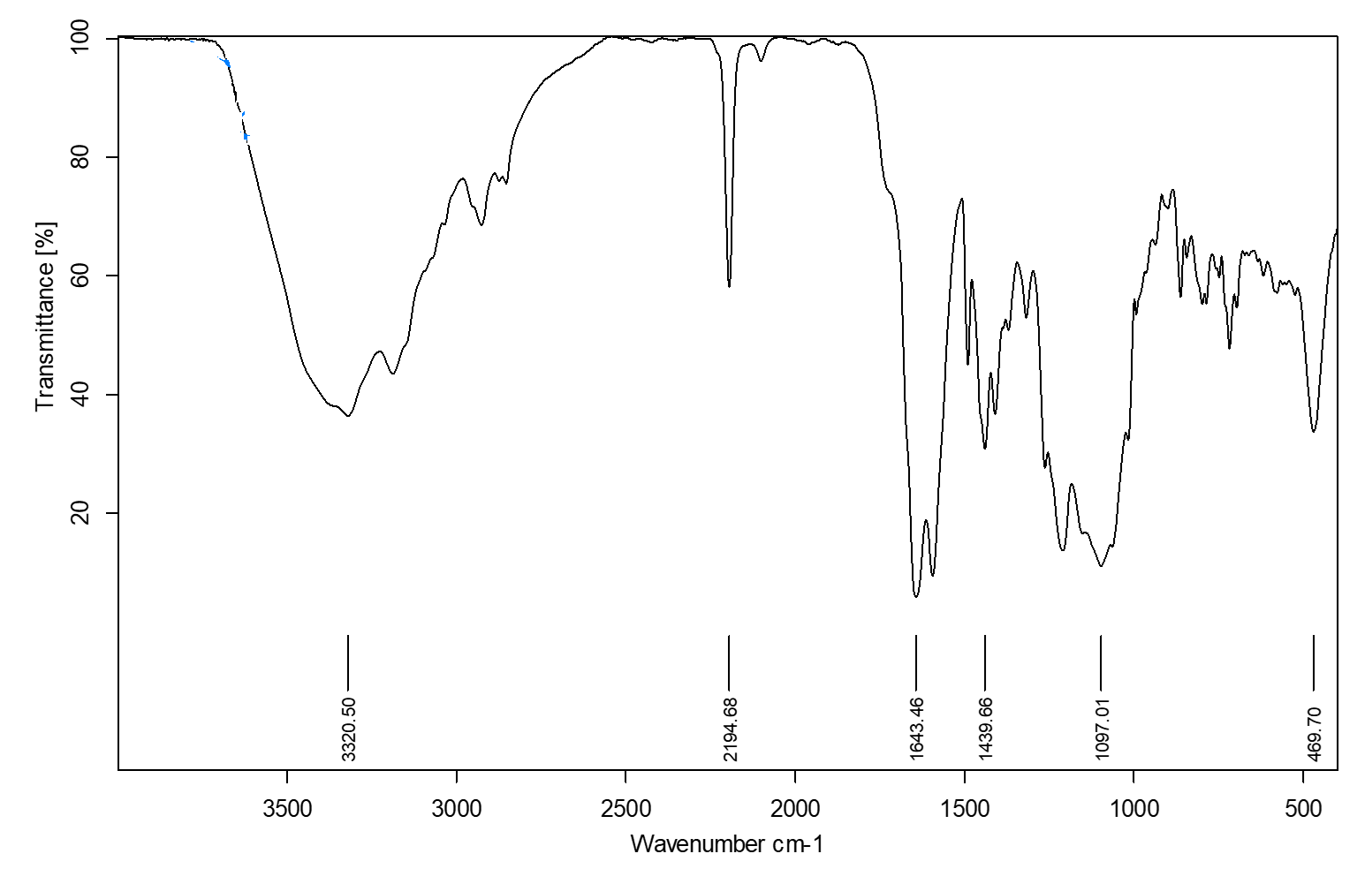


**
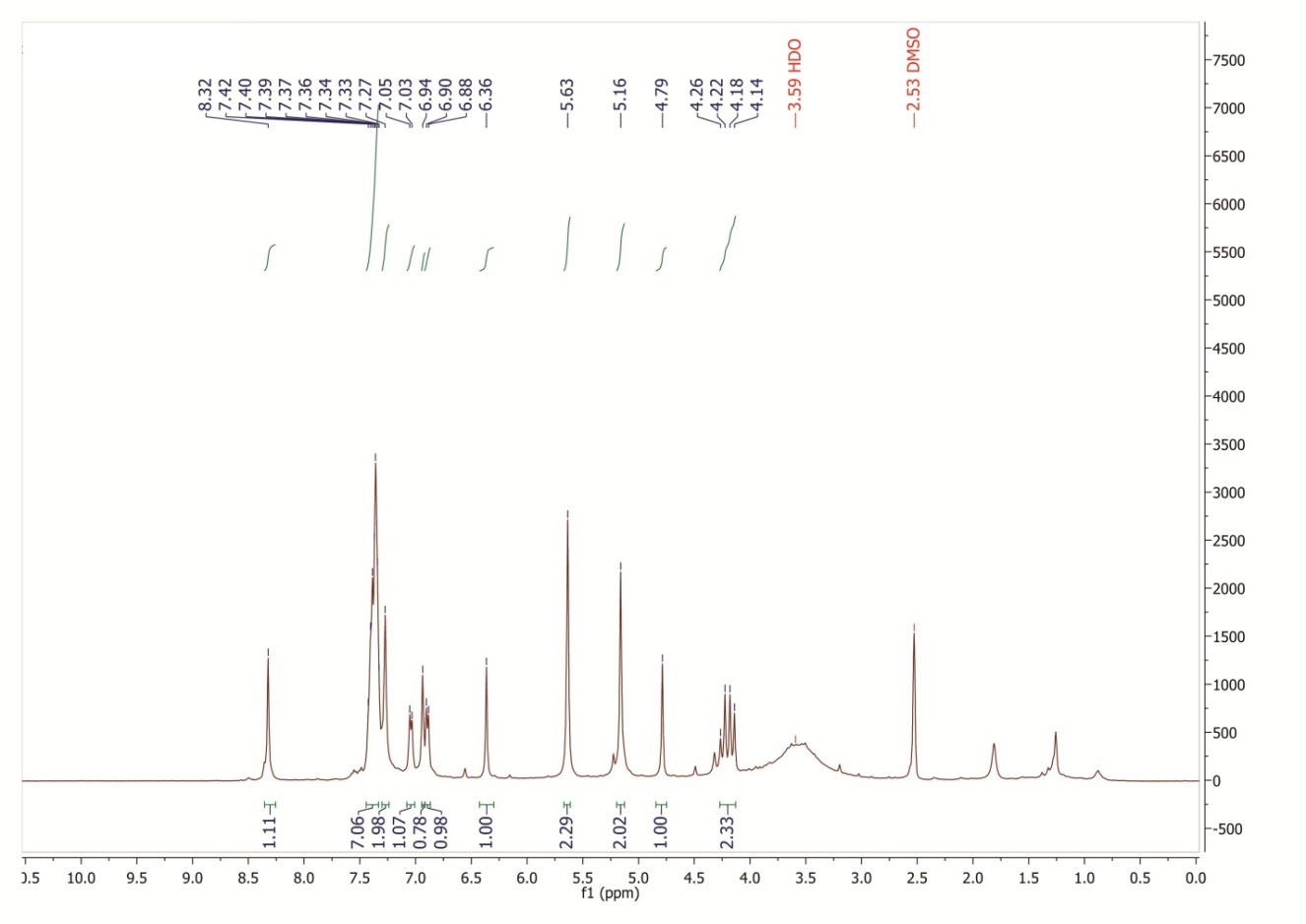

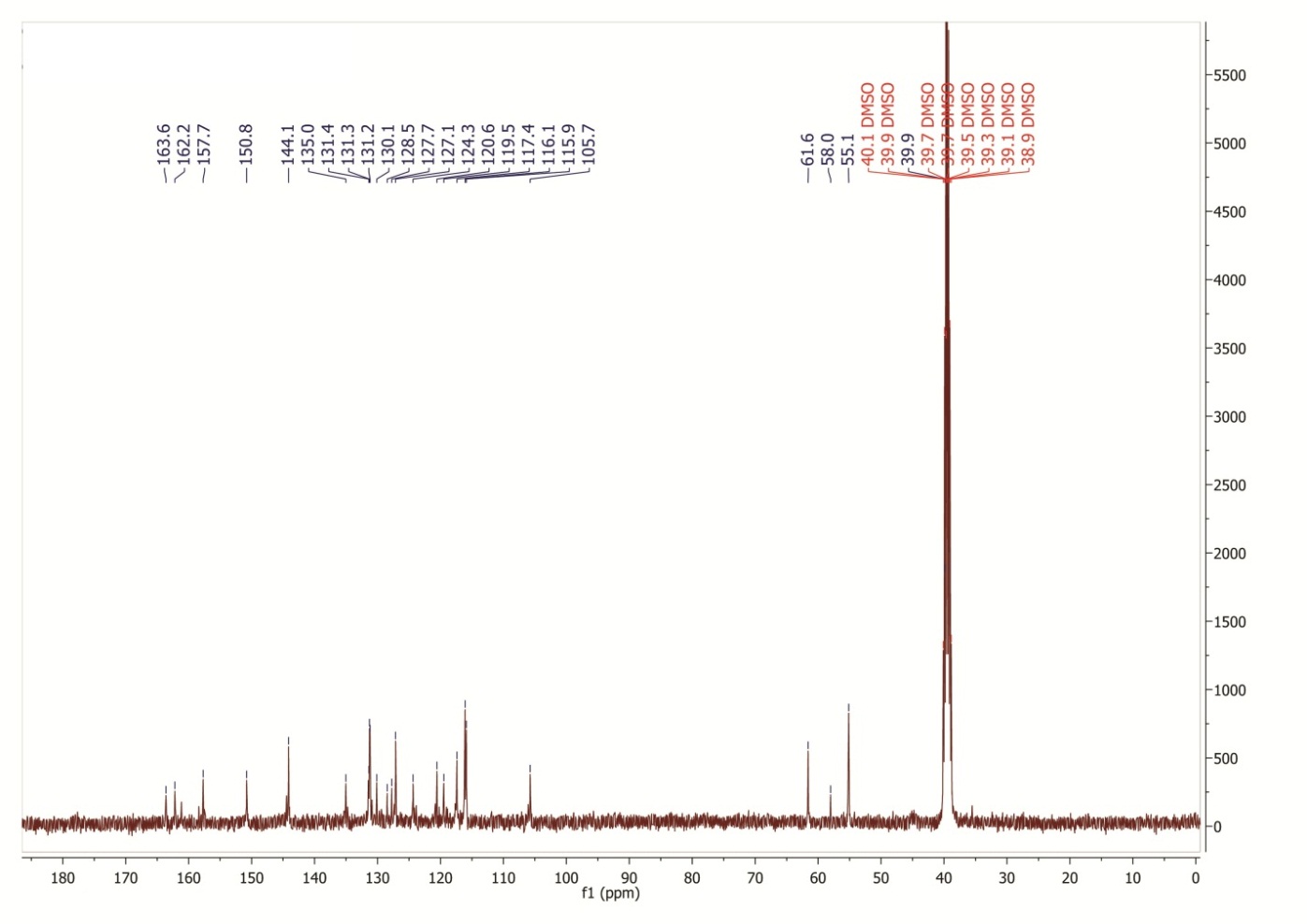
**

# 2-Amino-4-(3-((1-(4-chlorobenzyl)-1*H*-1,2,3-triazol-4-yl)methoxy)phenyl)-6-(hydroxymethyl)-8-oxo-4,8-dihydropyrano[3,2-*b*]pyran-3-carbonitrile (6g)


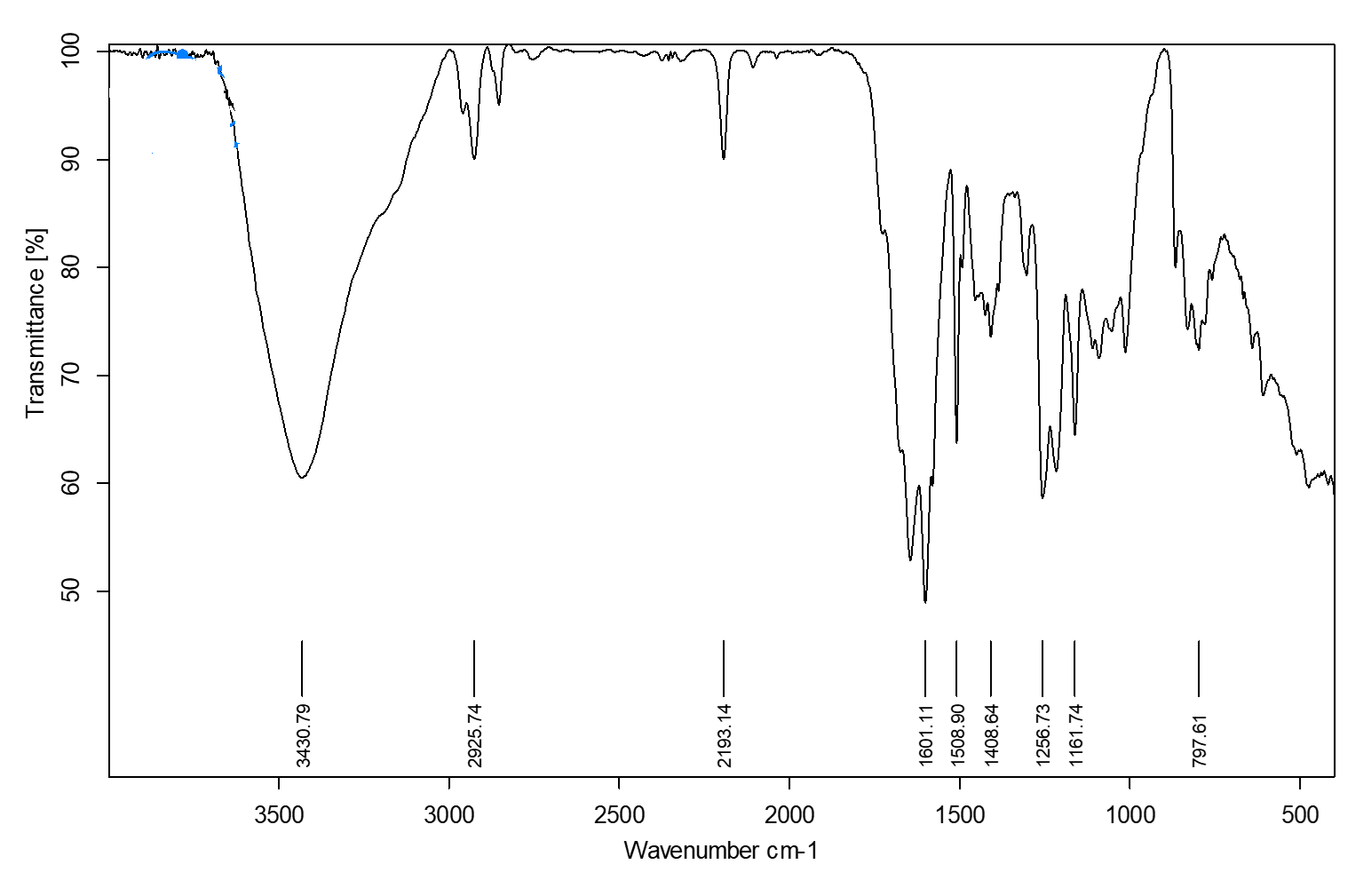


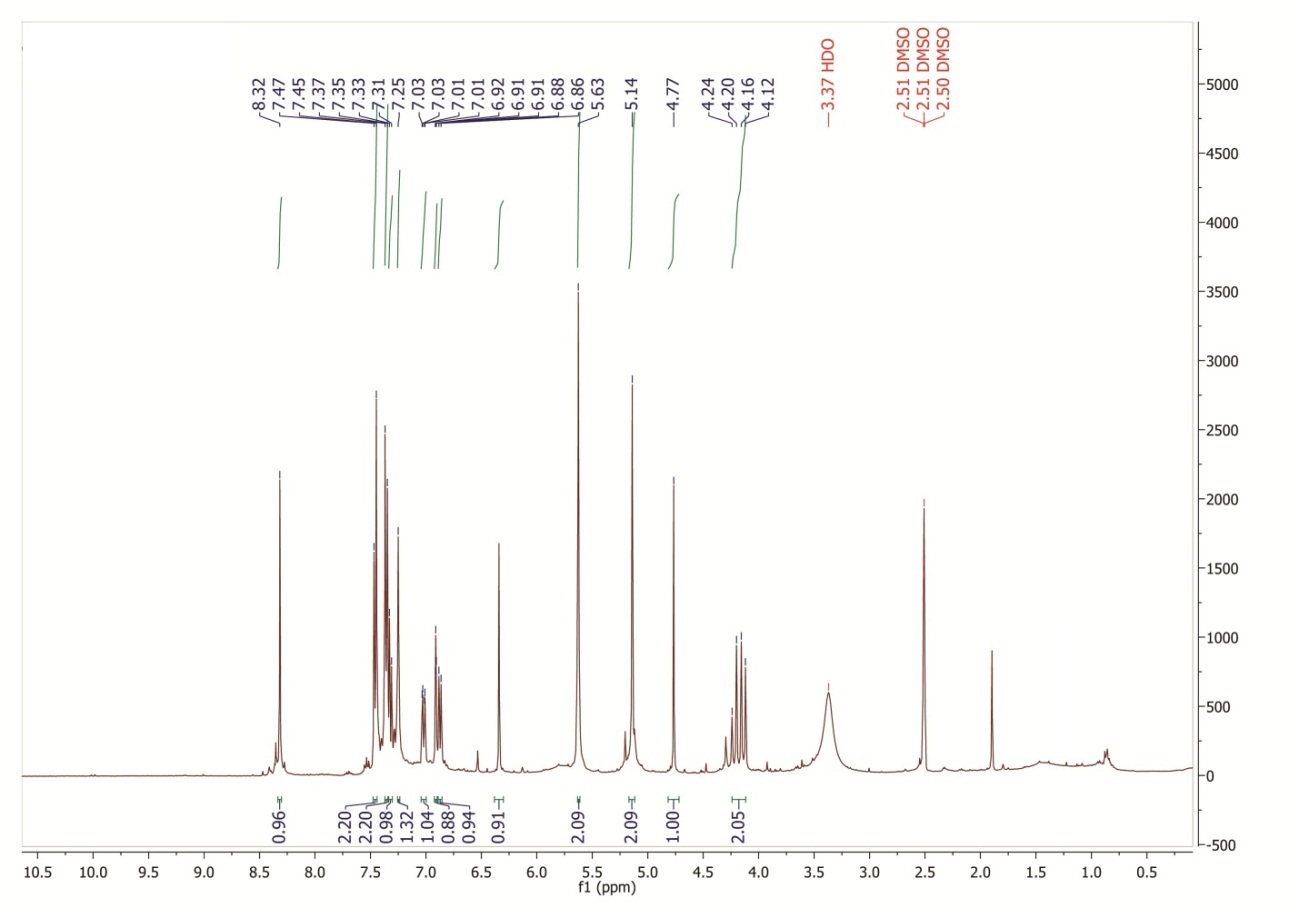

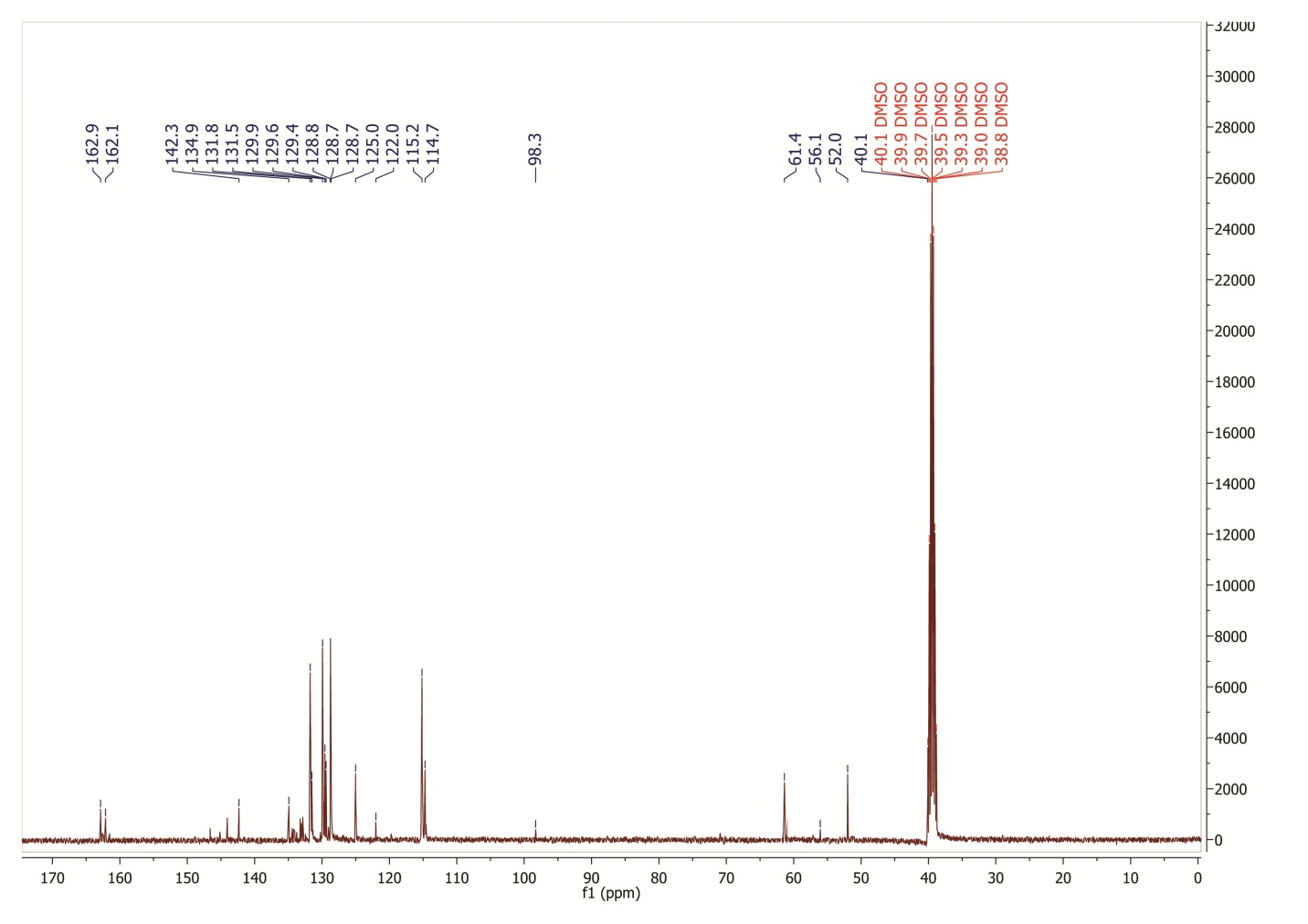

# 2-Amino-6-(hydroxymethyl)-4-(3-((1-(4-methylbenzyl)-1*H*-1,2,3-triazol-4-yl)methoxy)phenyl)-8-oxo-4,8-dihydropyrano[3,2-*b*]pyran-3-carbonitrile (6h)


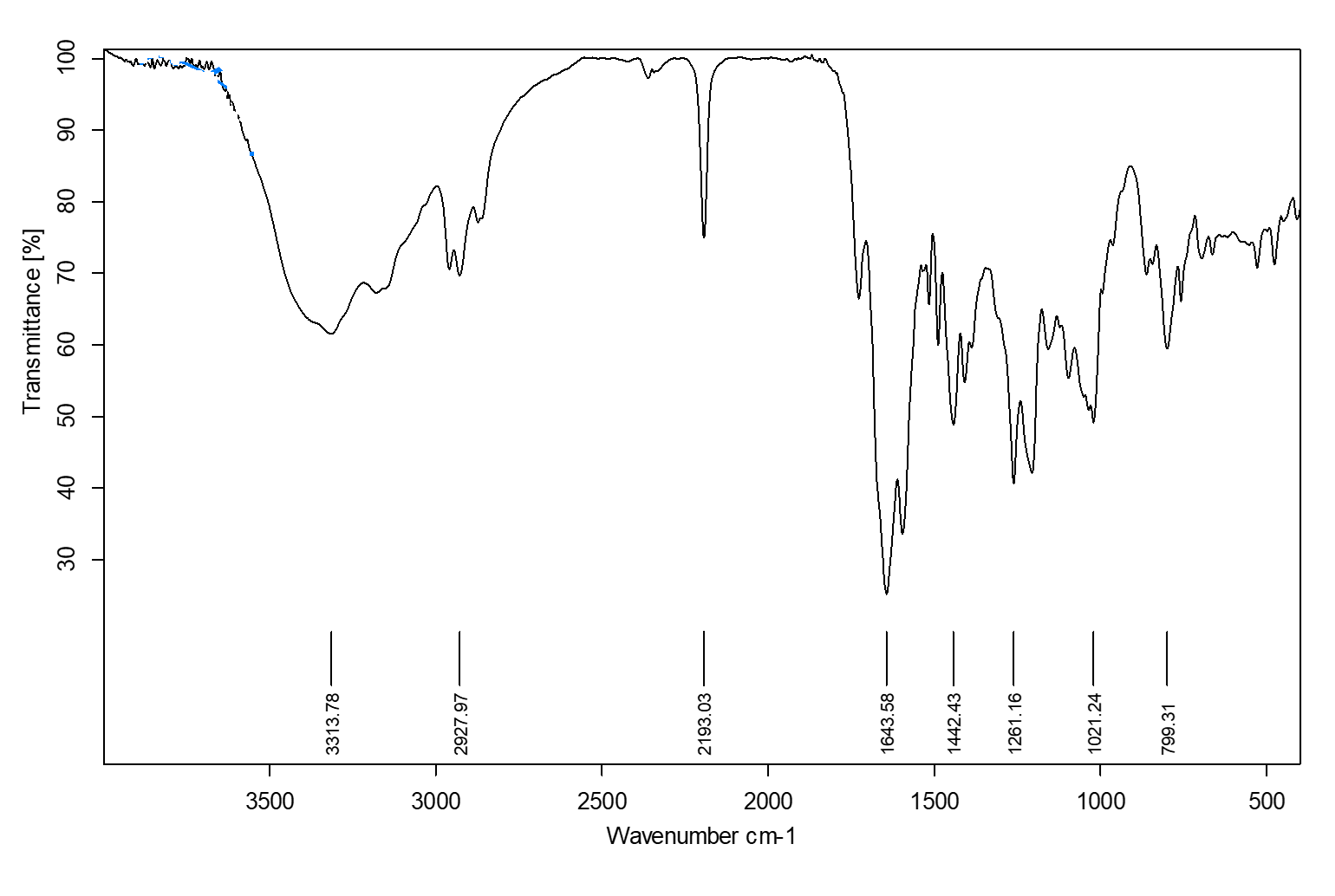


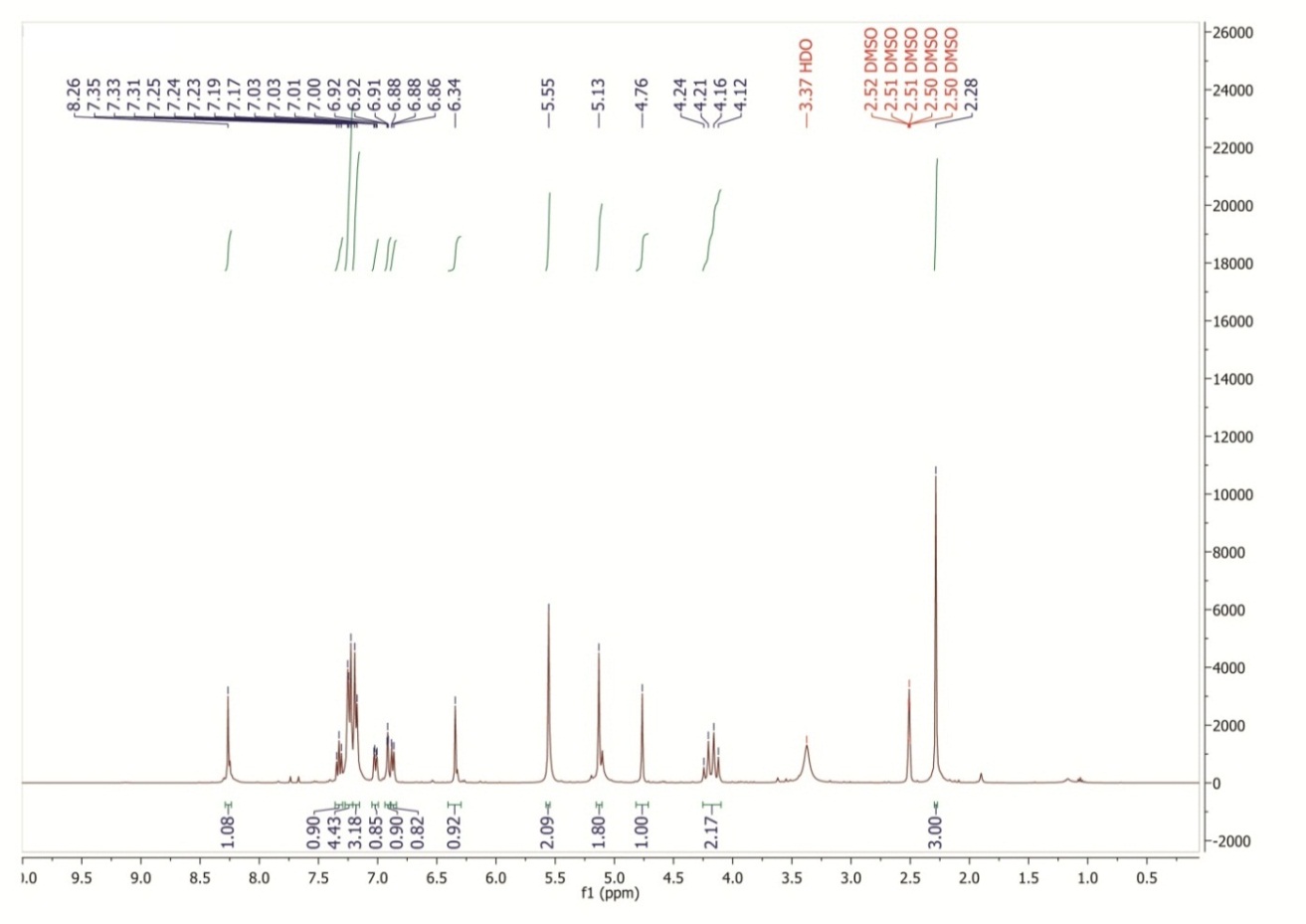


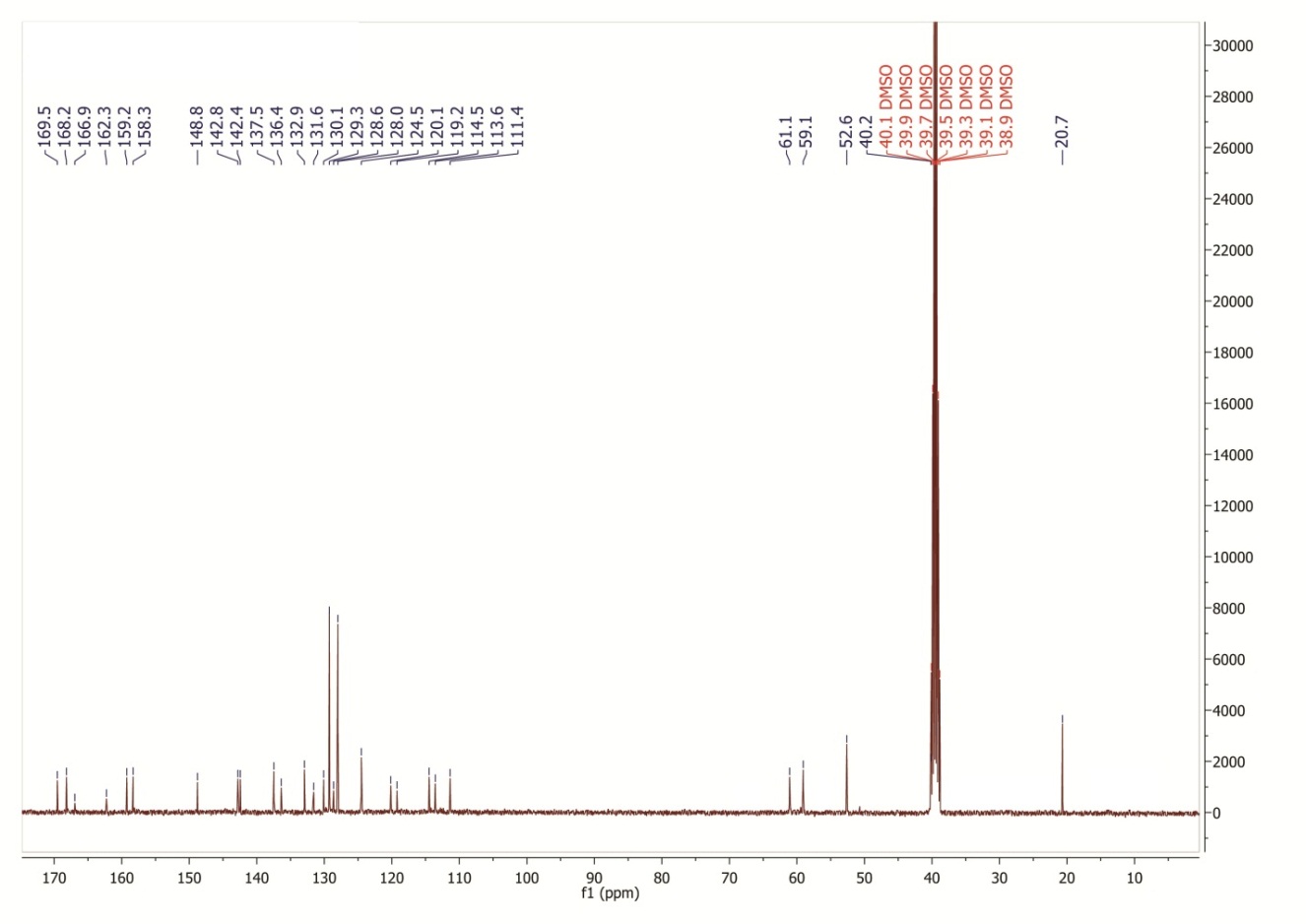

# 2-Amino-6-(hydroxymethyl)-4-(3-((1-(4-methoxybenzyl)-1*H*-1,2,3-triazol-4-yl)methoxy)phenyl)-8-oxo-4,8-dihydropyrano[3,2-*b*]pyran-3-carbonitrile (6i)


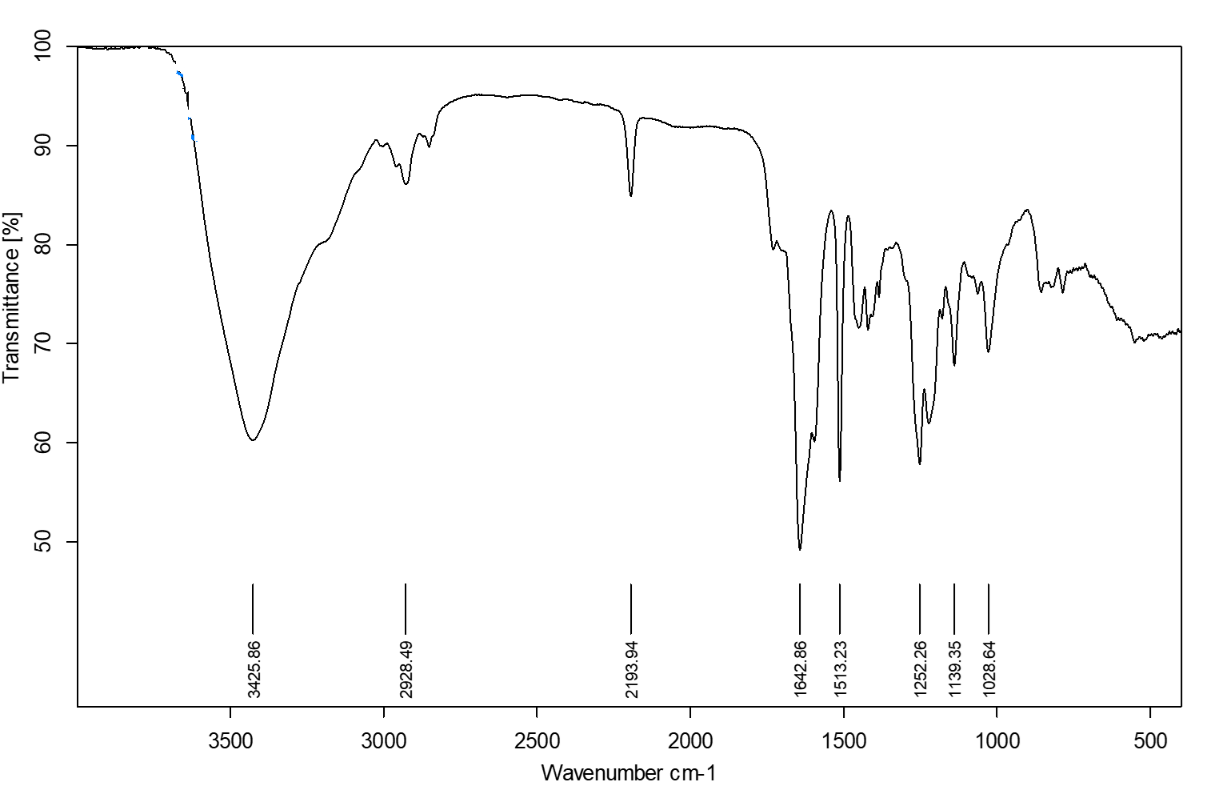


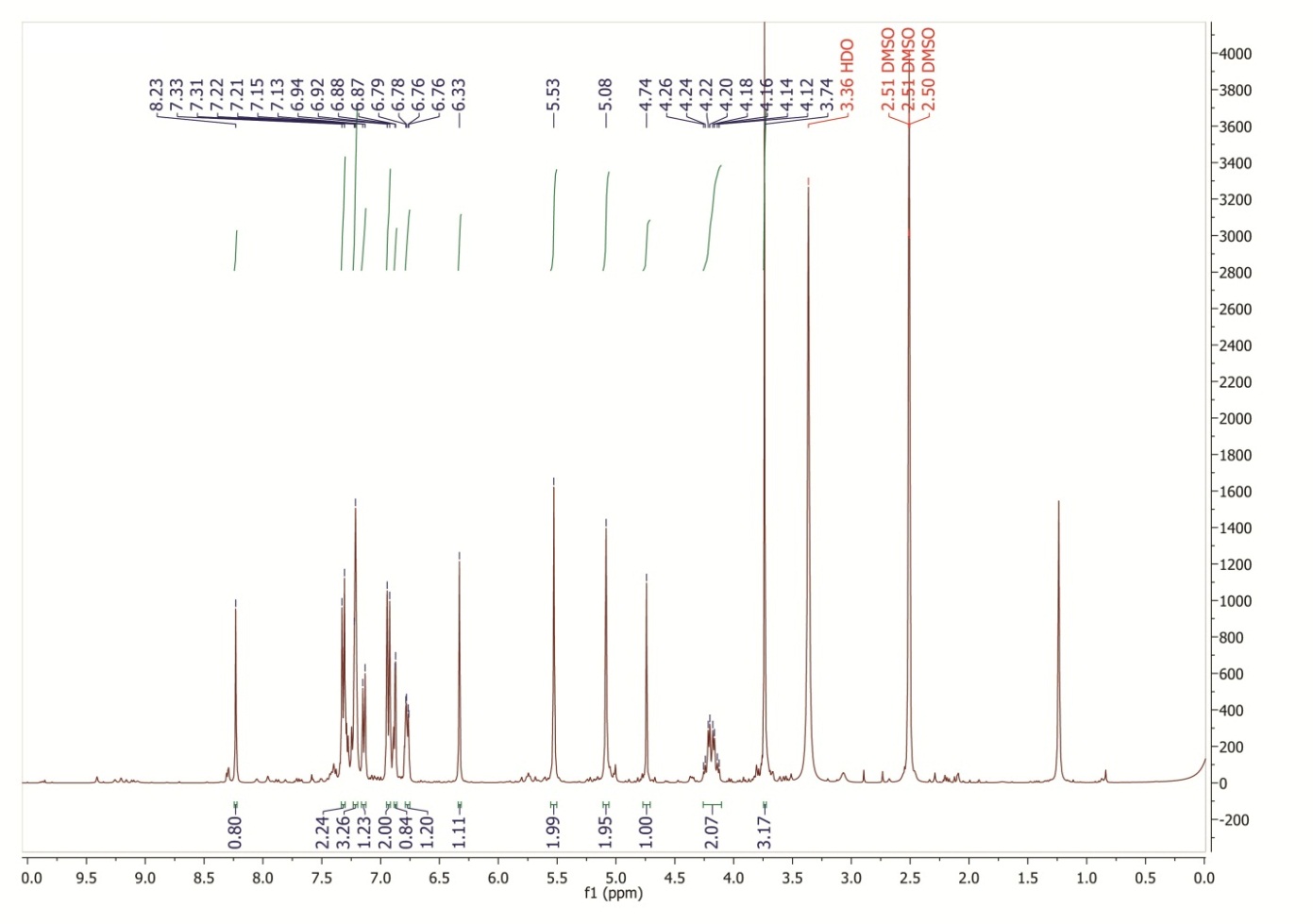

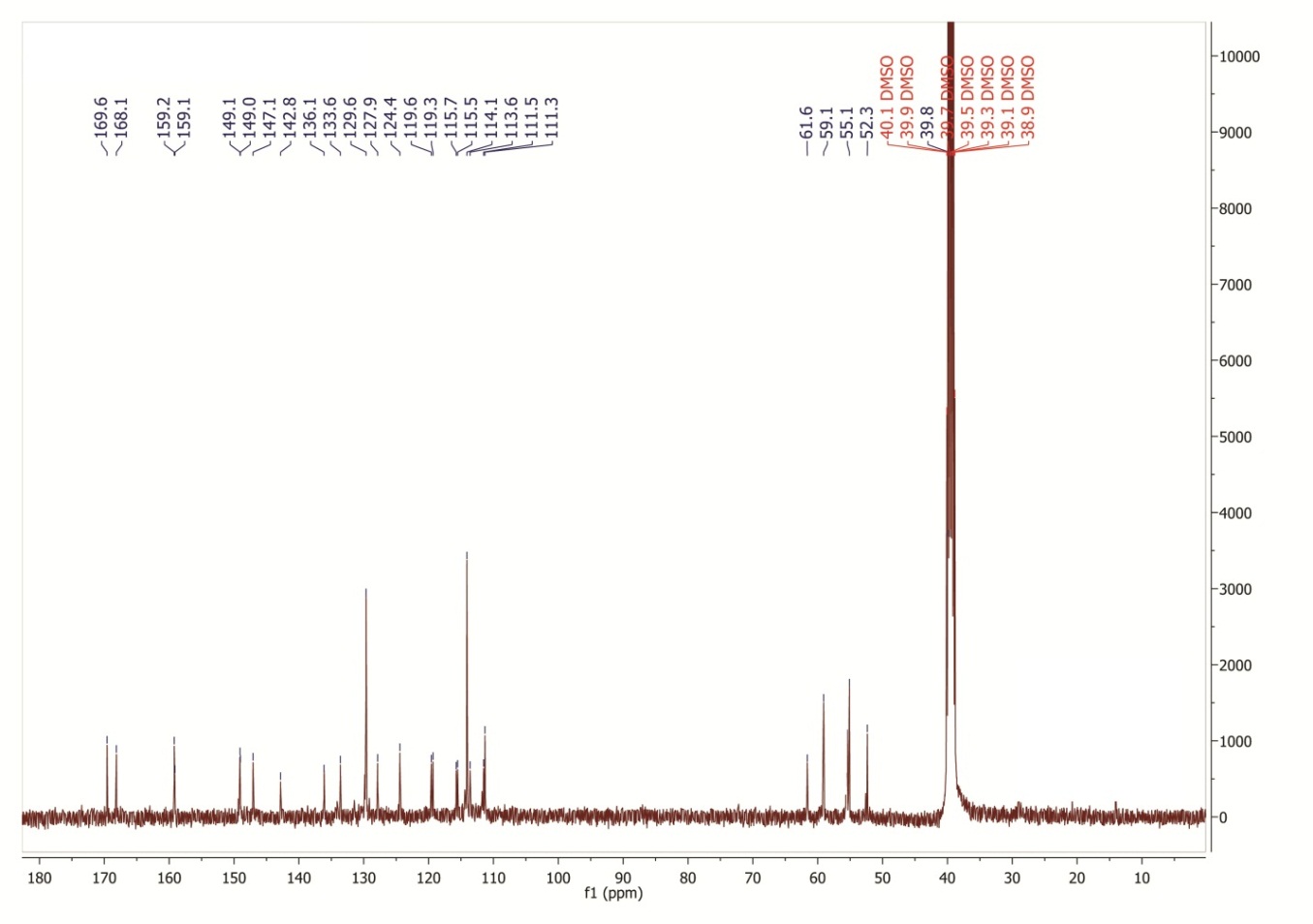

# 2-Amino-4-(3-((1-(4-fluorobenzyl)-1*H*-1,2,3-triazol-4-yl)methoxy)phenyl)-6-(hydroxymethyl)-8-oxo-4,8-dihydropyrano[3,2-*b*]pyran-3-carbonitrile (6j)


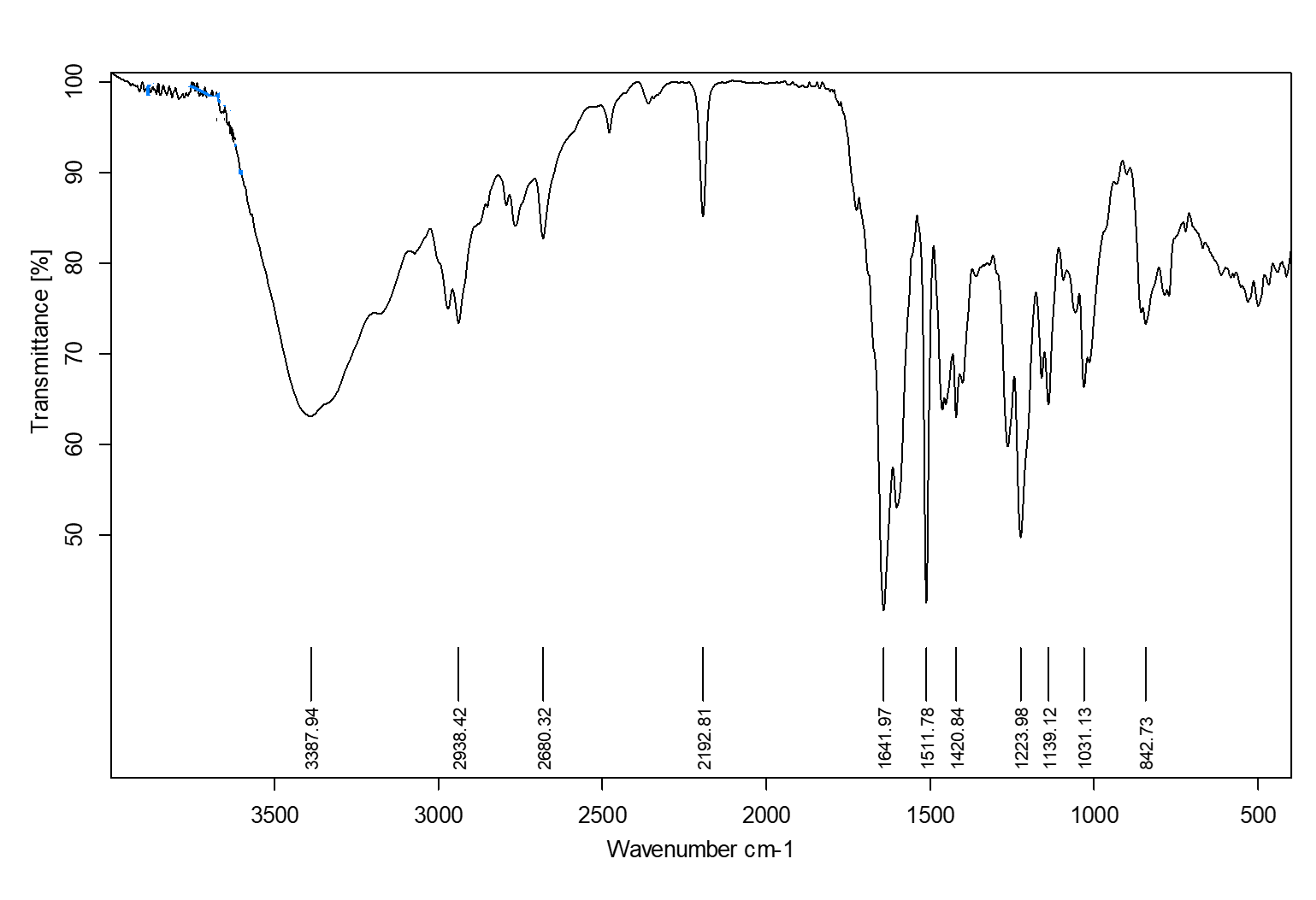


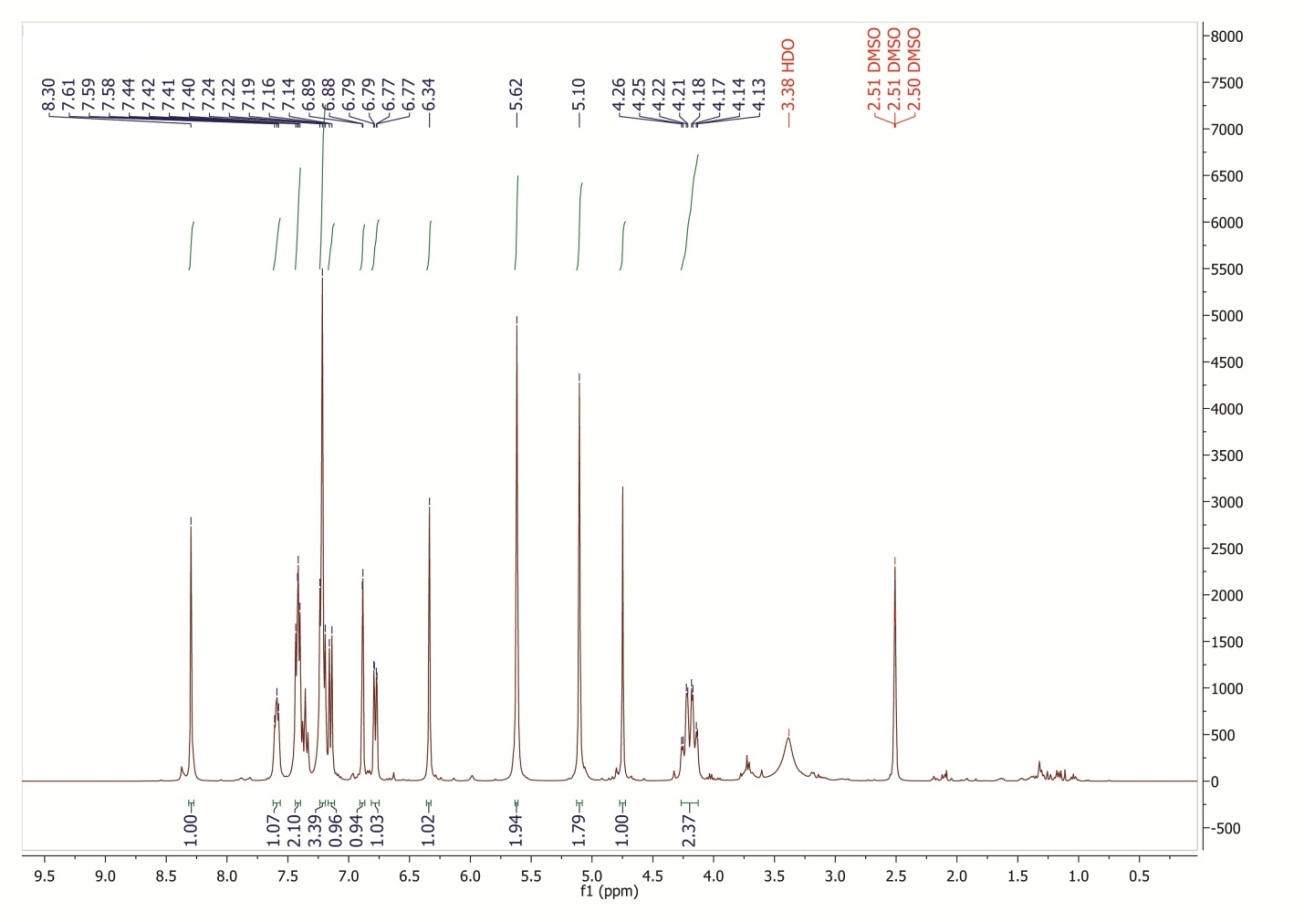


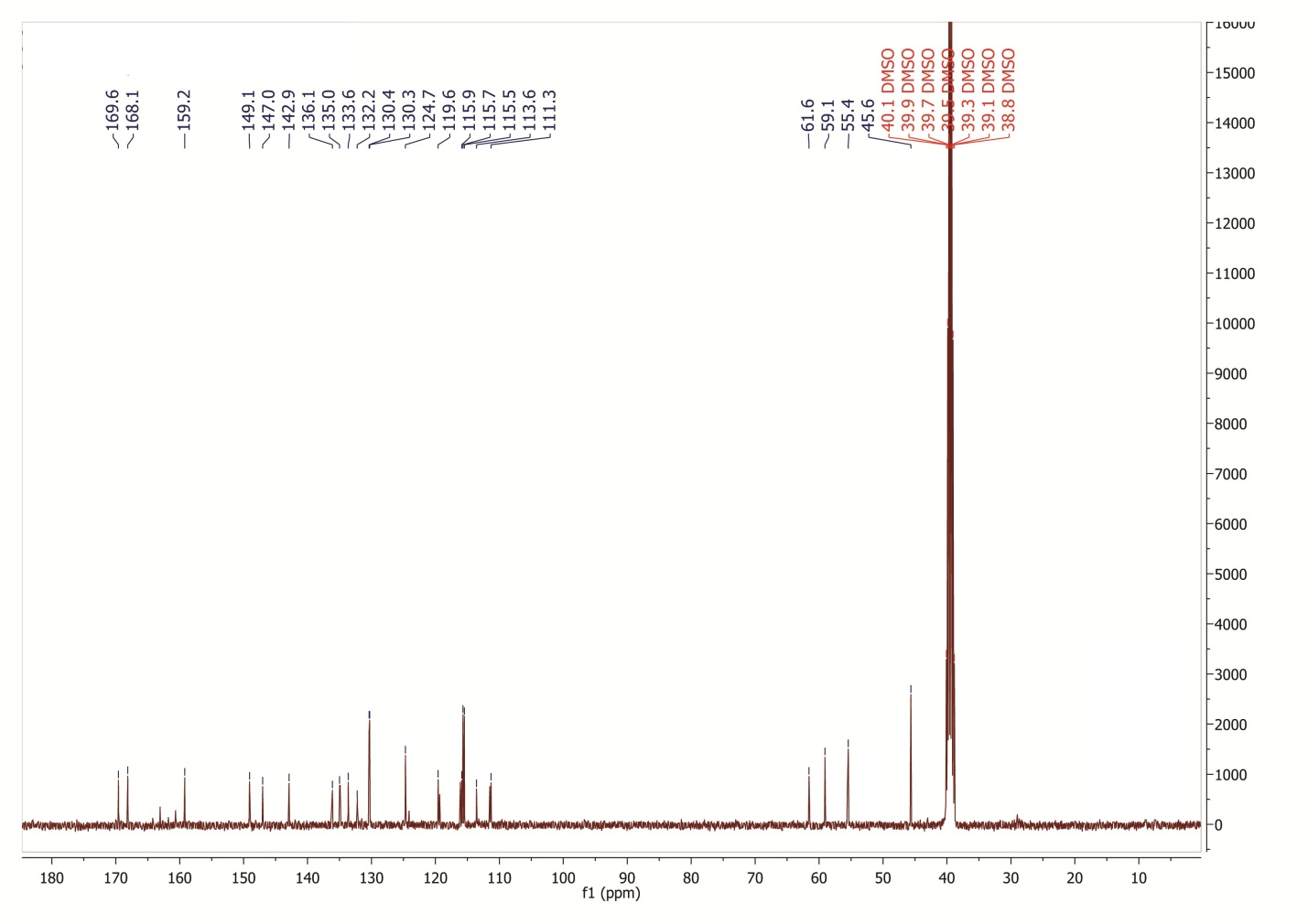

# 2-Amino-4-(4-((1-benzyl-1*H*-1,2,3-triazol-4-yl)methoxy)-3-methoxyphenyl)-6-(hydroxymethyl)-8-oxo-4,8-dihydropyrano[3,2-*b*]pyran-3-carbonitrile (6k)


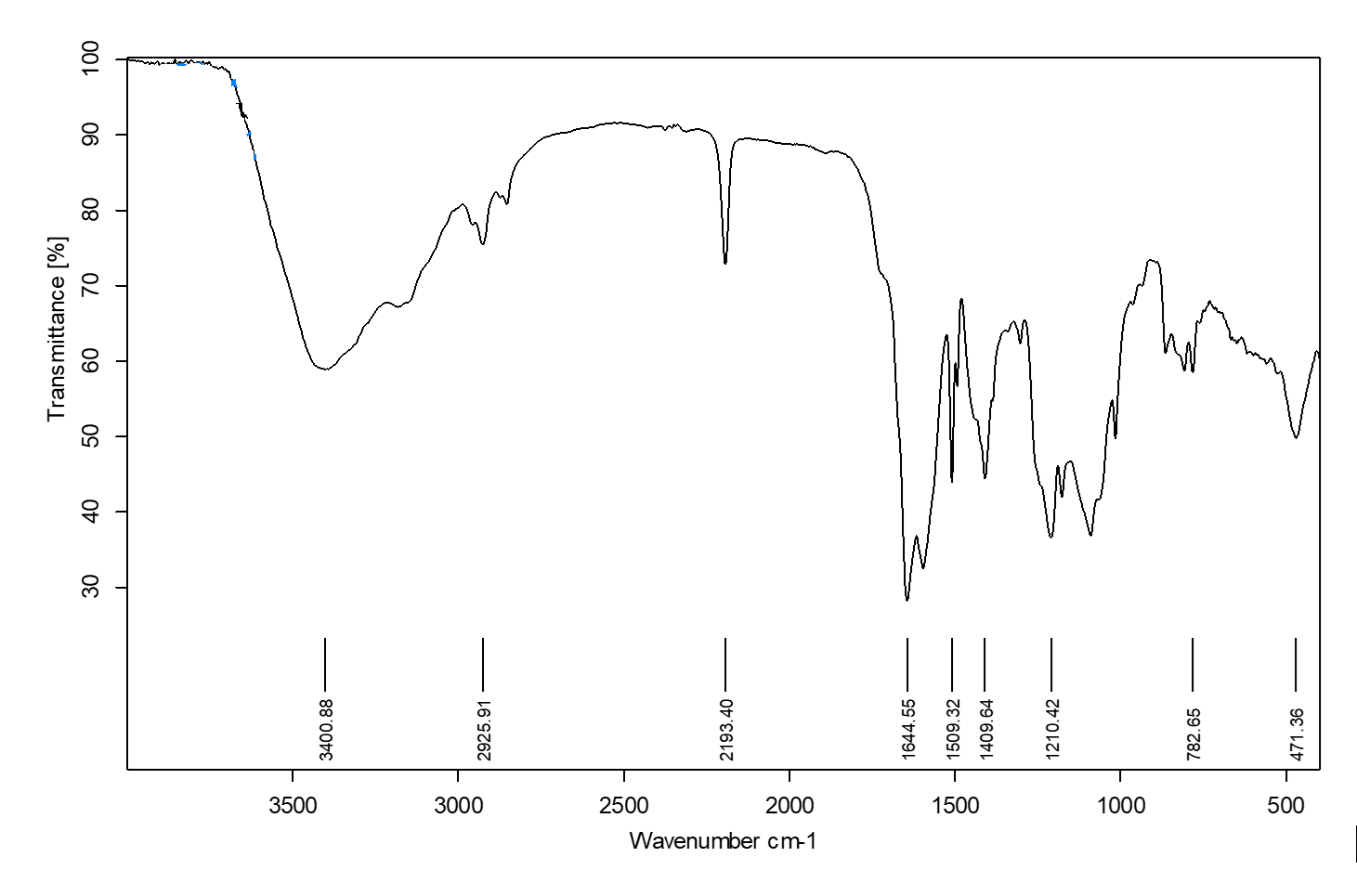


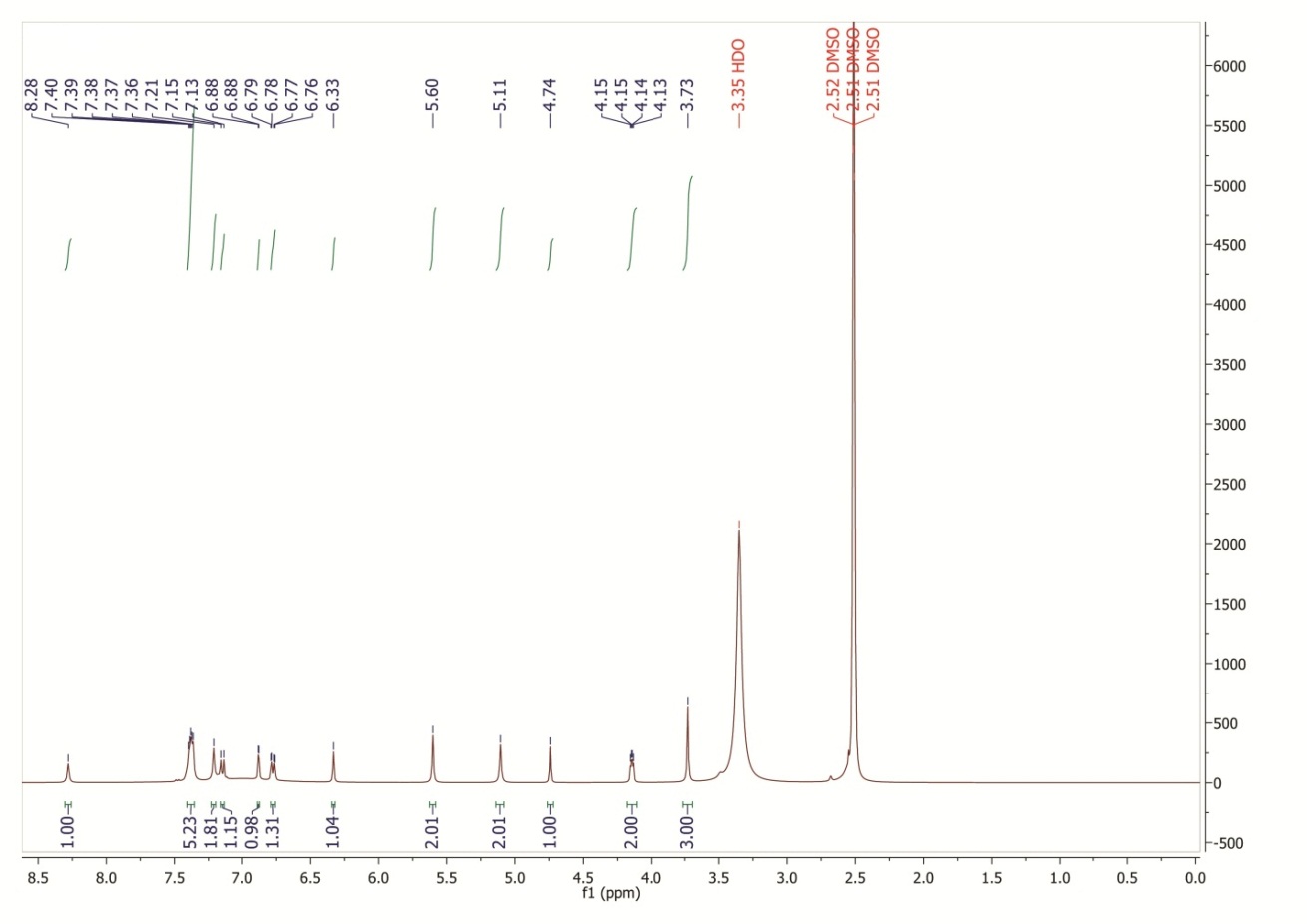

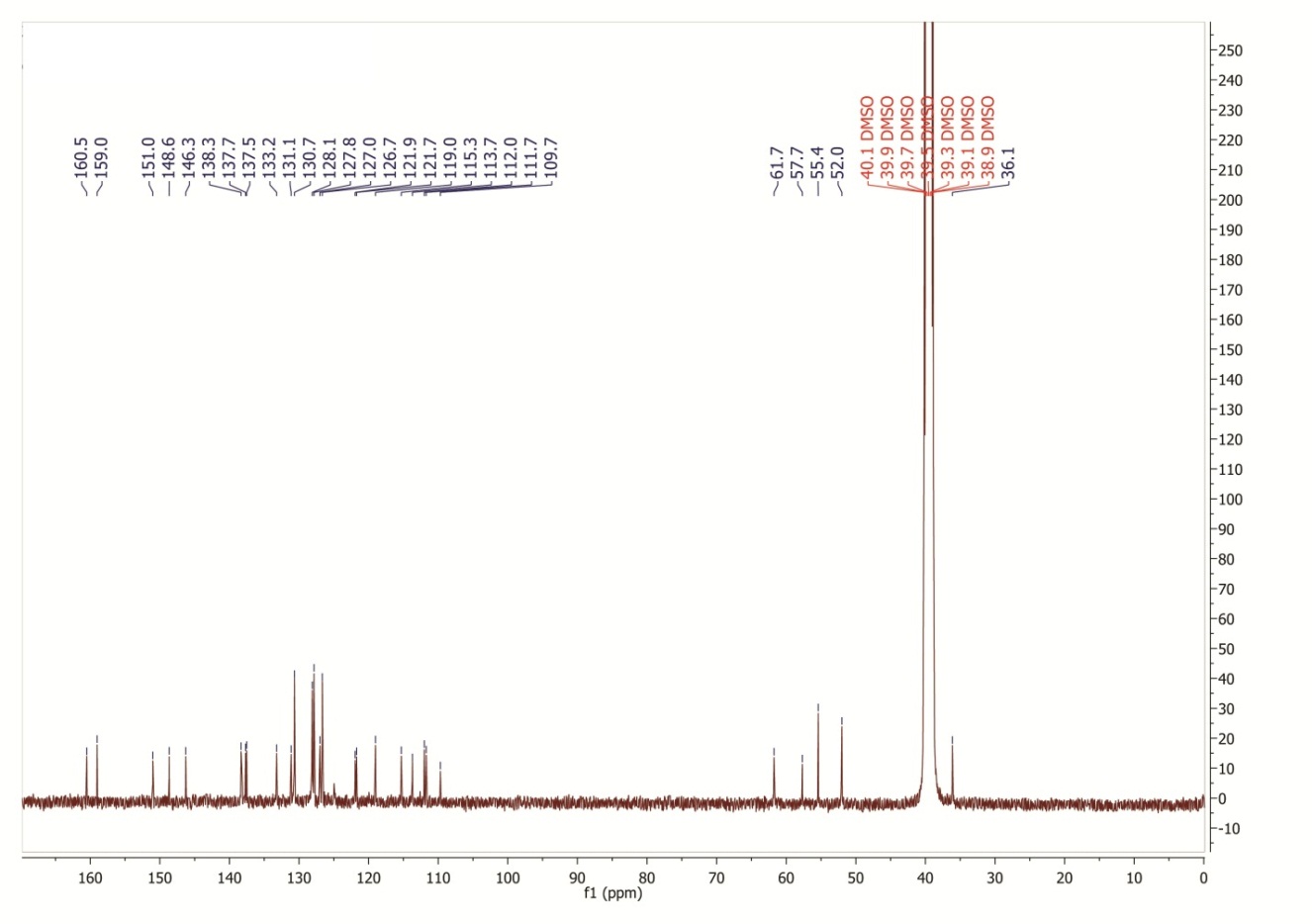

# 2-Amino-4-(4-((1-(4-chlorobenzyl)-1*H*-1,2,3-triazol-4-yl)methoxy)-3-methoxyphenyl)-6-(hydroxymethyl)-8-oxo-4,8-dihydropyrano[3,2-*b*]pyran-3-carbonitrile (6l)


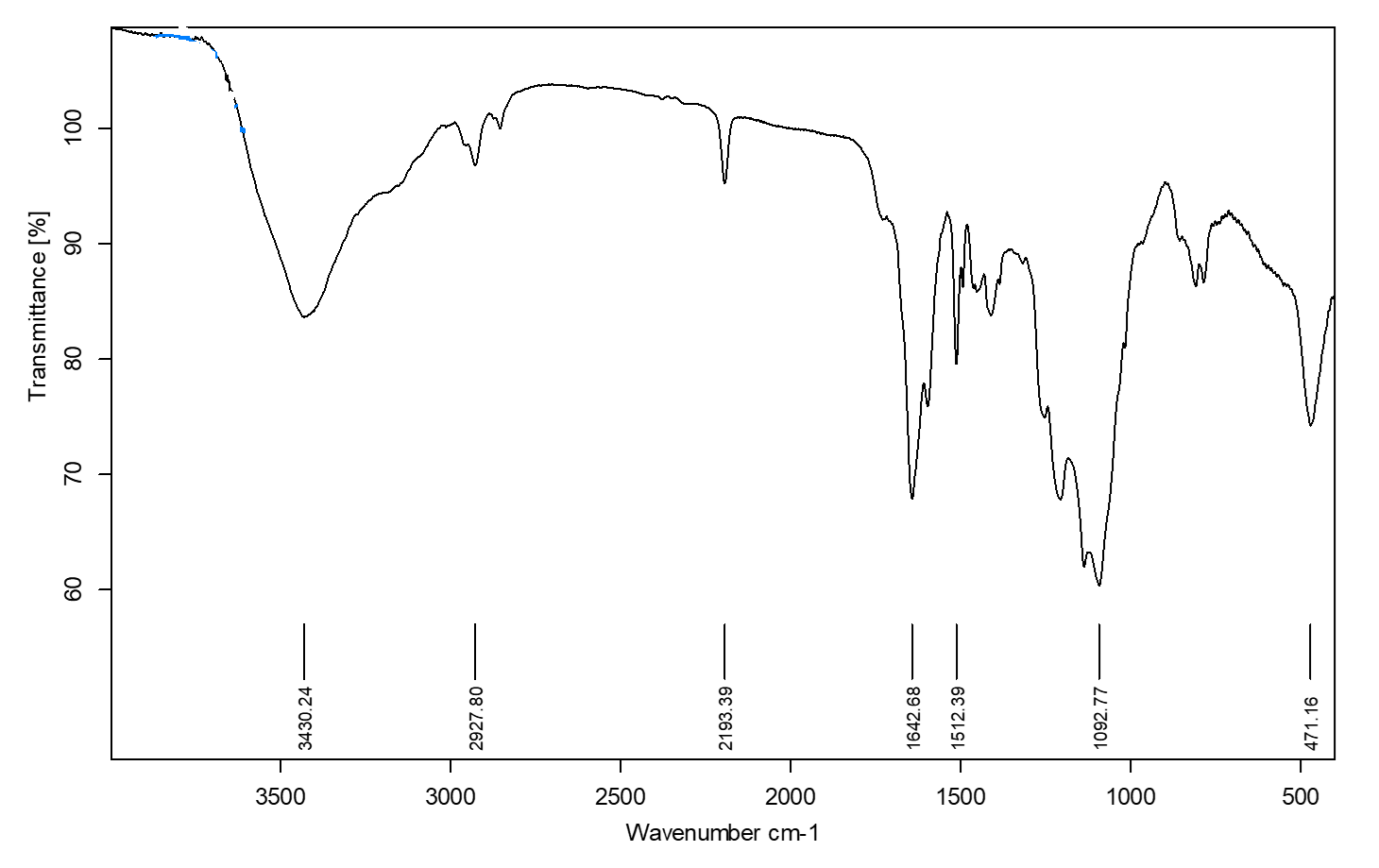


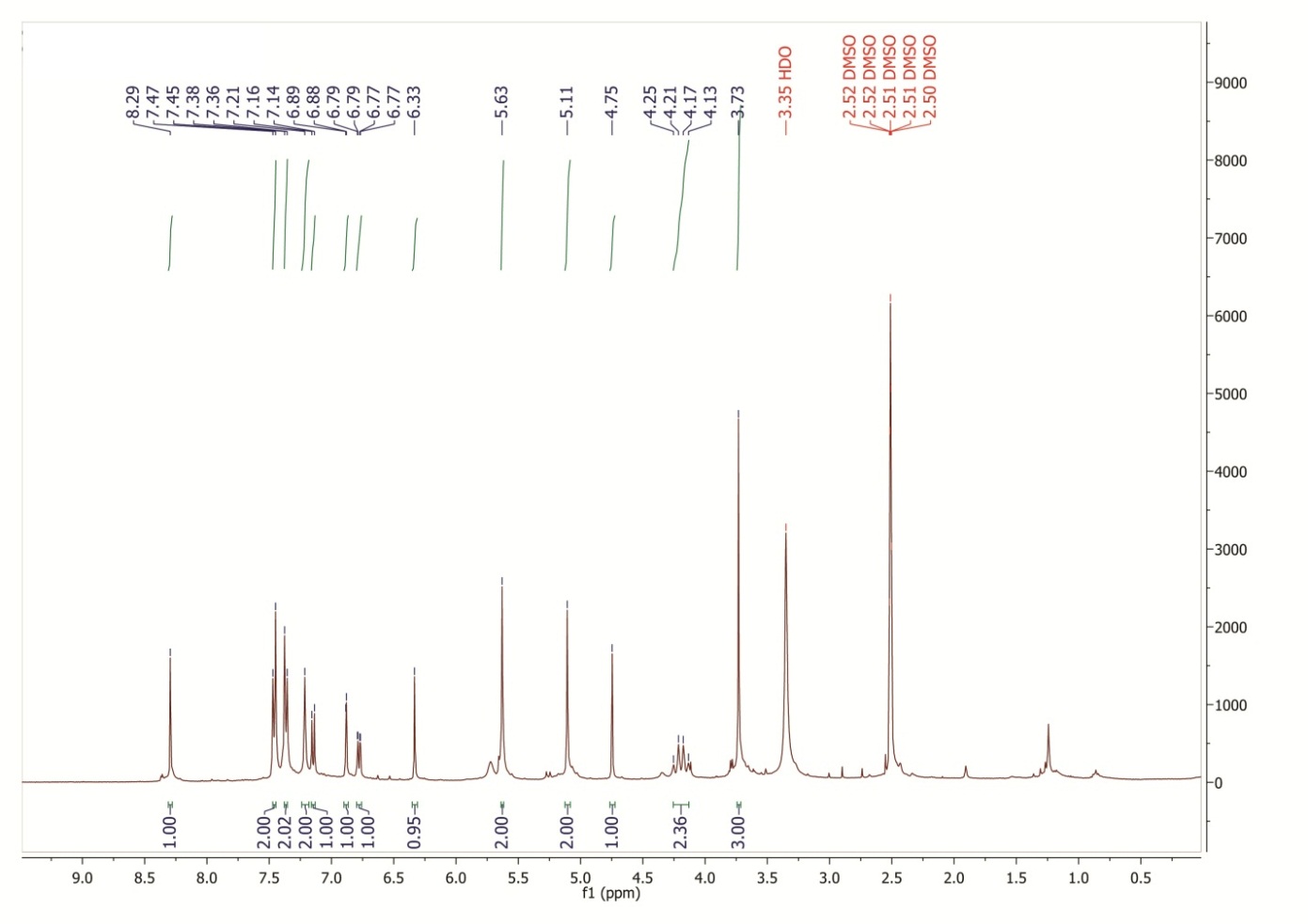


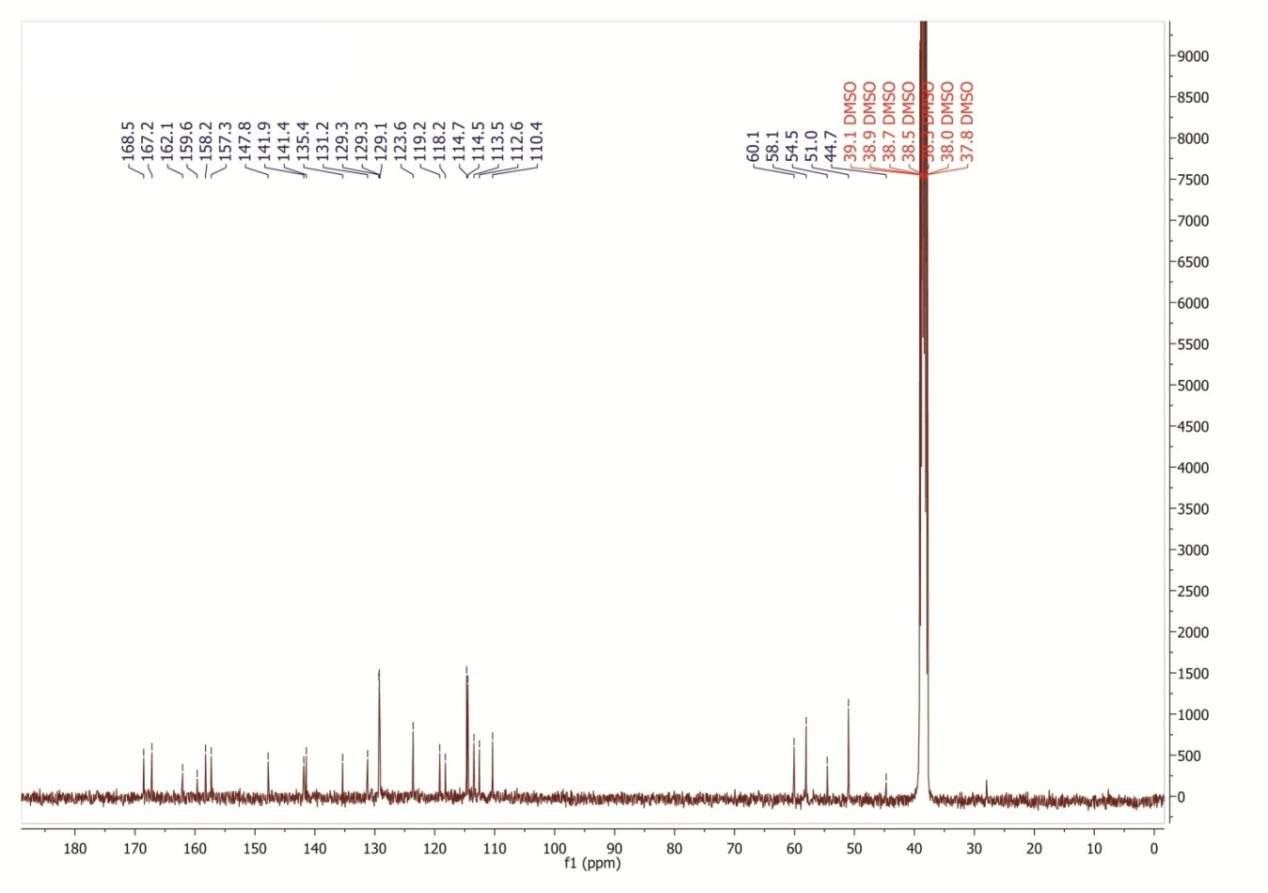

# 2-Amino-6-(hydroxymethyl)-4-(3-methoxy-4-((1-(4-methylbenzyl)-1*H*-1,2,3-triazol-4-yl)methoxy)phenyl)-8-oxo-4,8-dihydropyrano[3,2-*b*]pyran-3-carbonitrile (6m)


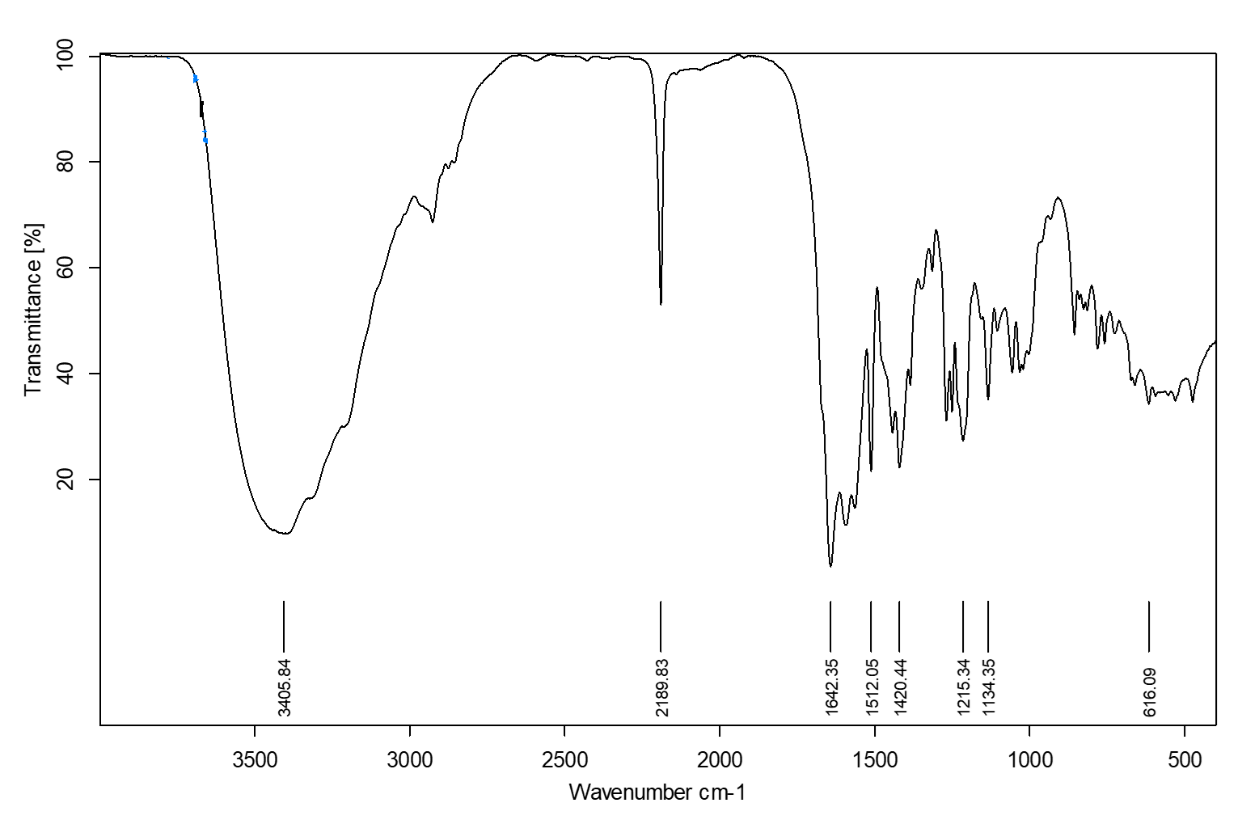


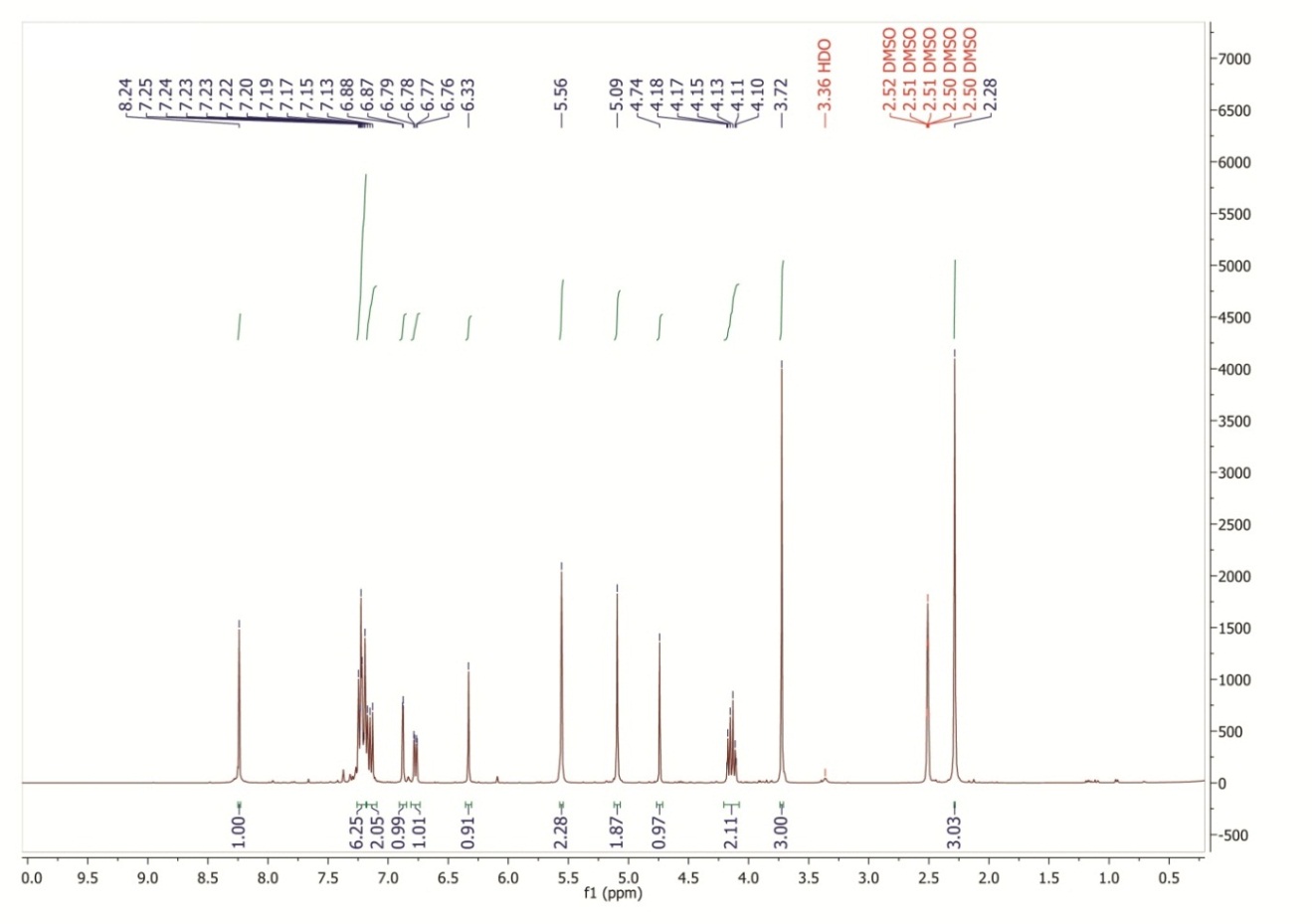


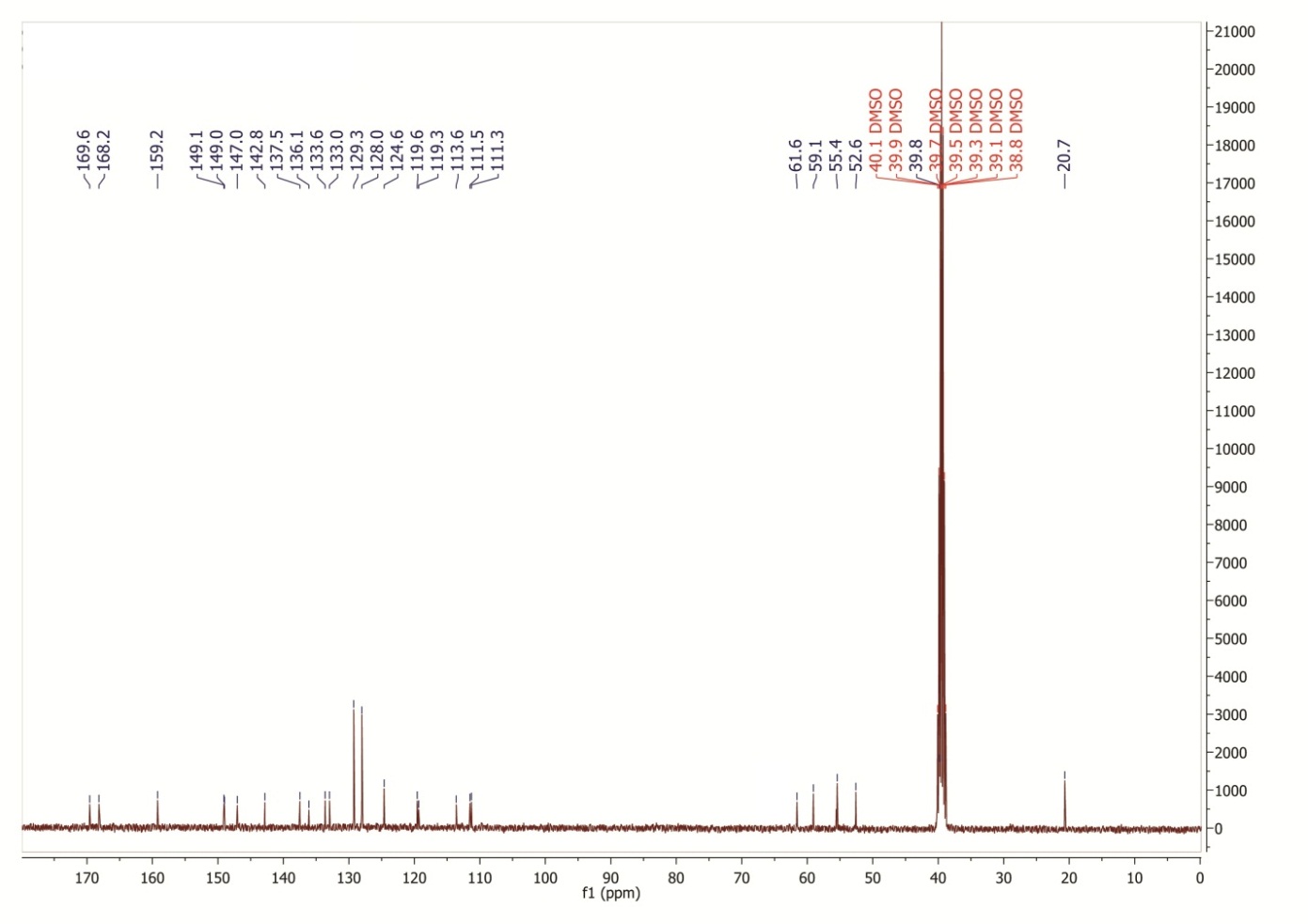

# 2-Amino-6-(hydroxymethyl)-4-(3-methoxy-4-((1-(4-methoxybenzyl)-1*H*-1,2,3-triazol-4-yl)methoxy)phenyl)-8-oxo-4,8-dihydropyrano[3,2-*b*]pyran-3-carbonitrile (6n)


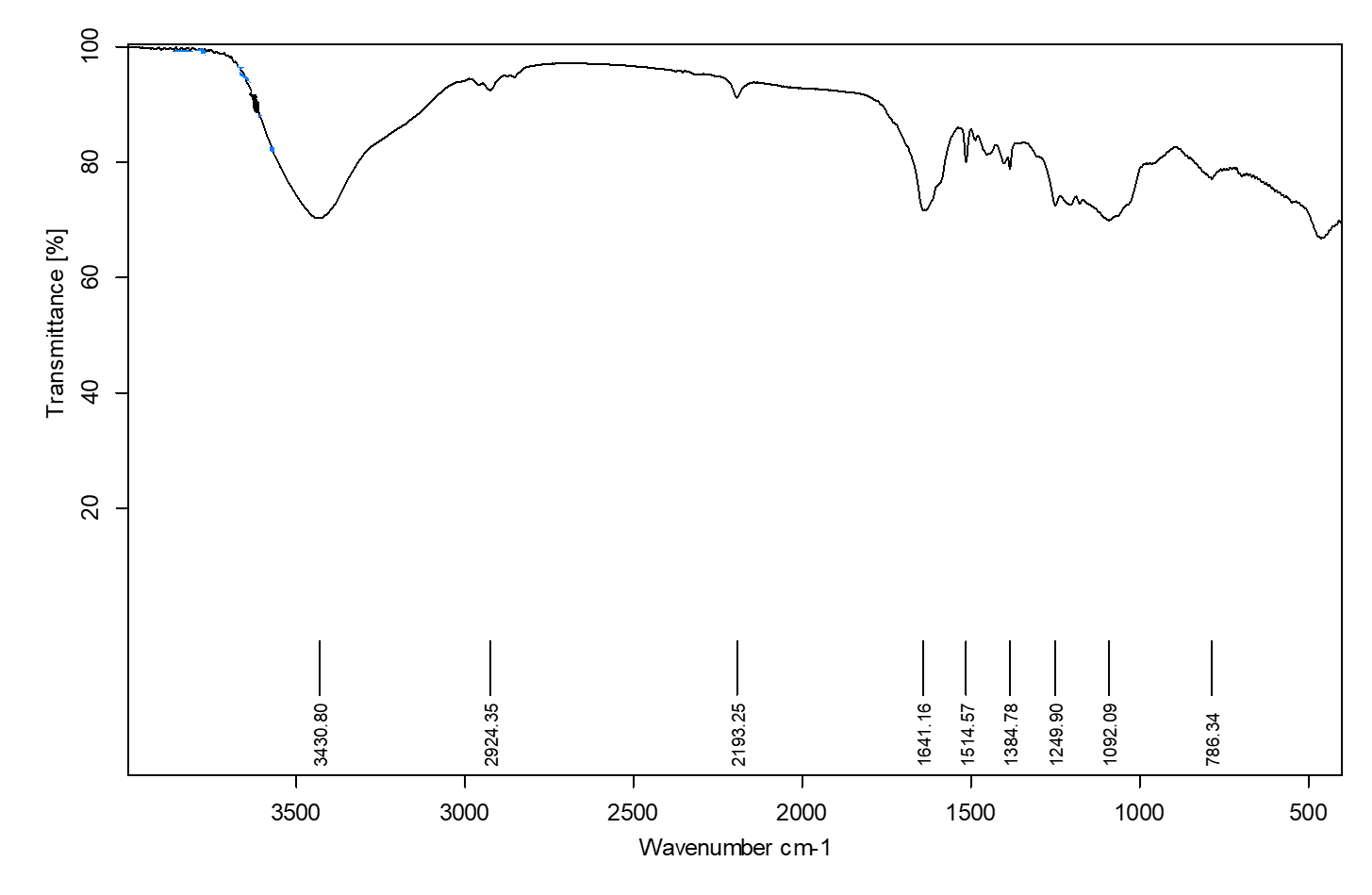


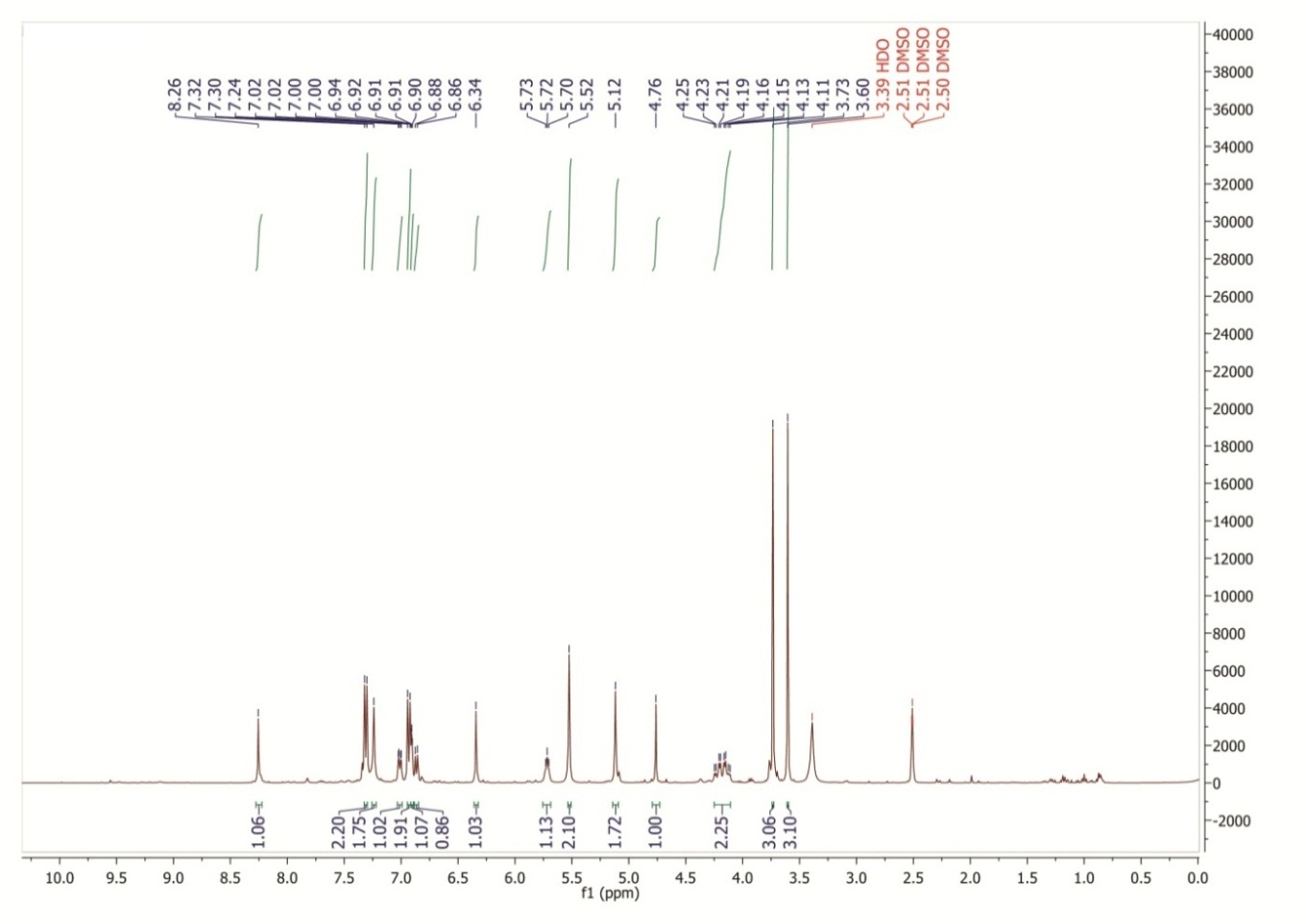


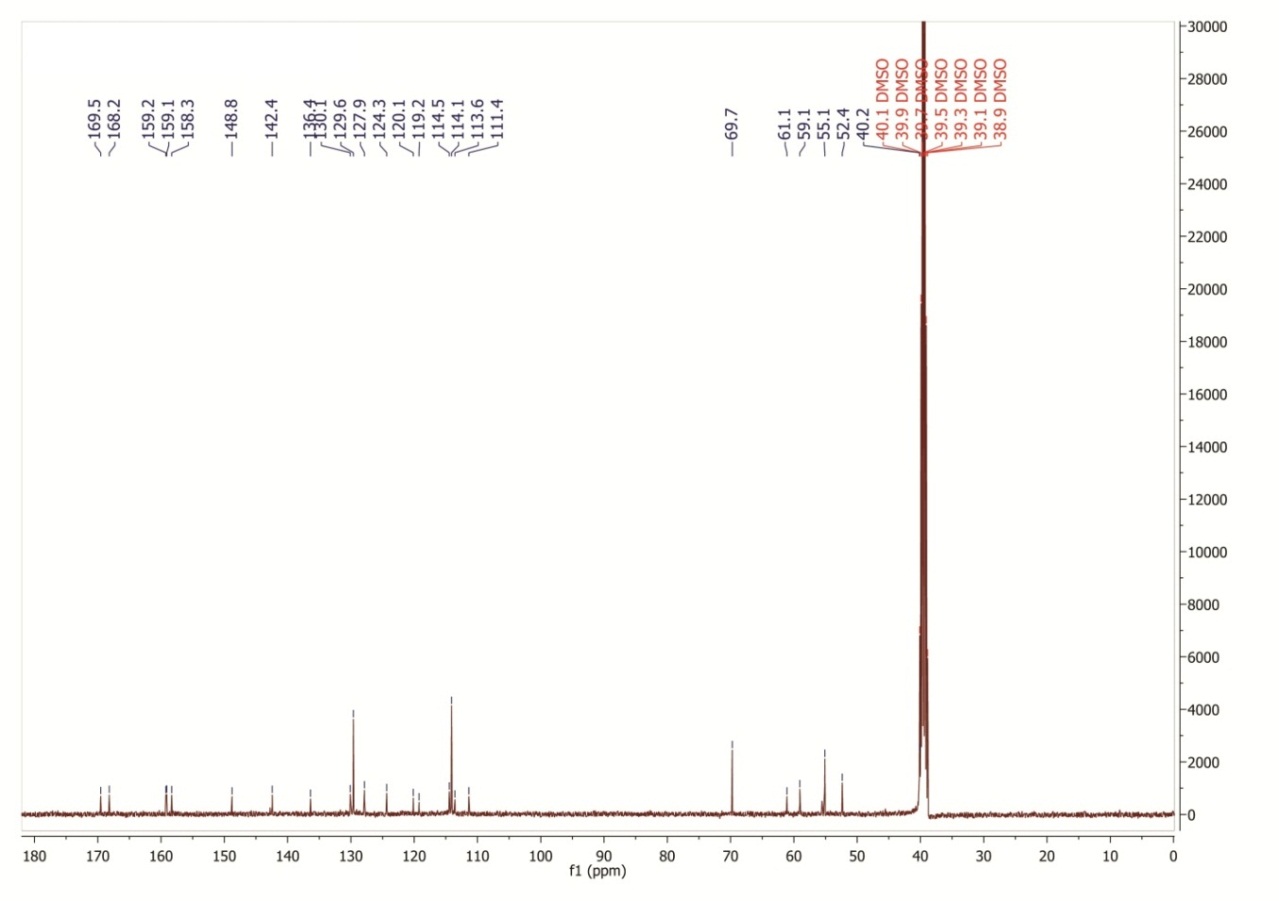

# 2-Amino-4-(4-((1-(4-fluorobenzyl)-1*H*-1,2,3-triazol-4-yl)methoxy)-3-methoxyphenyl)-6-(hydroxymethyl)-8-oxo-4,8-dihydropyrano[3,2-*b*]pyran-3-carbonitrile (6o)


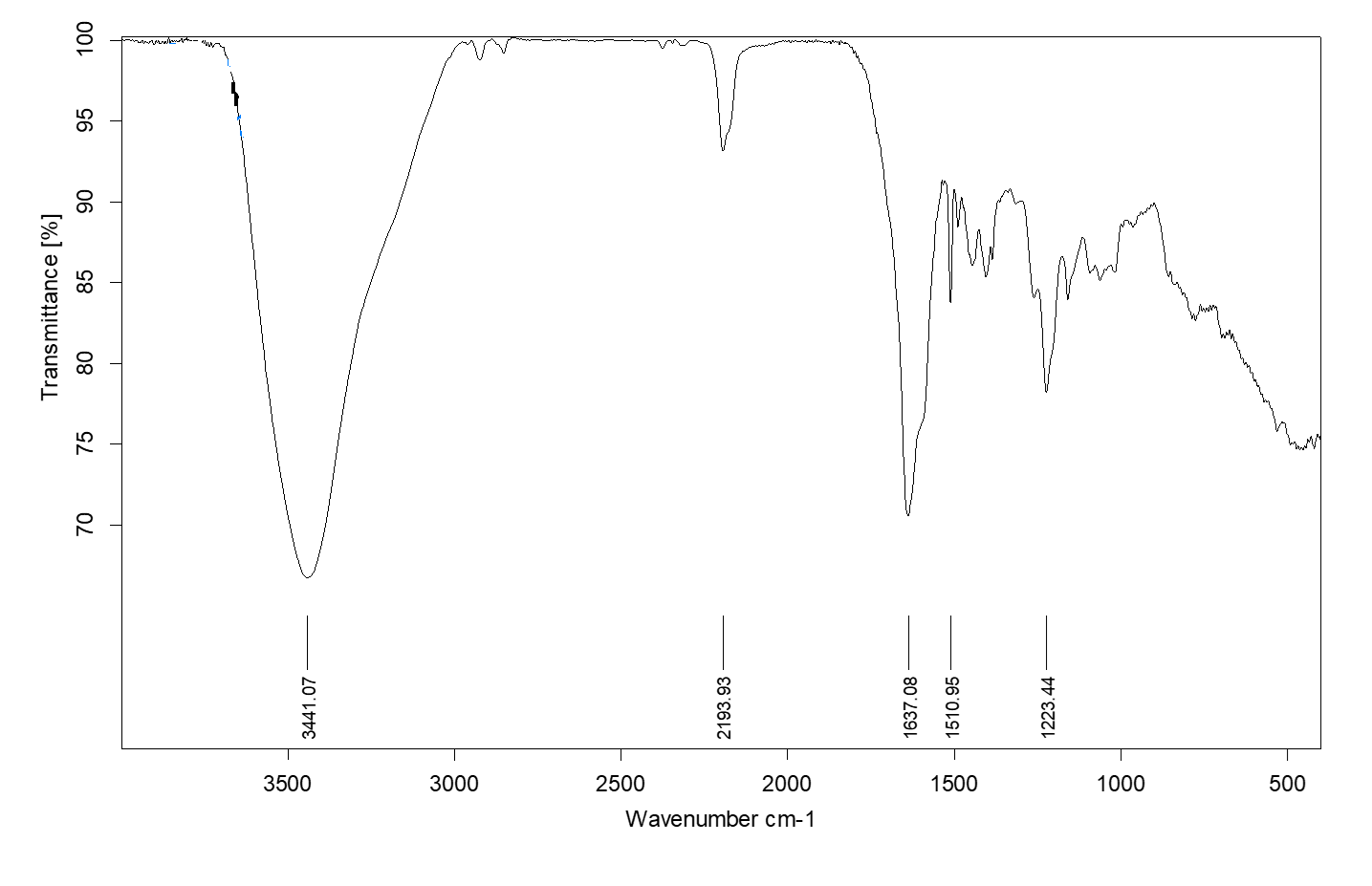


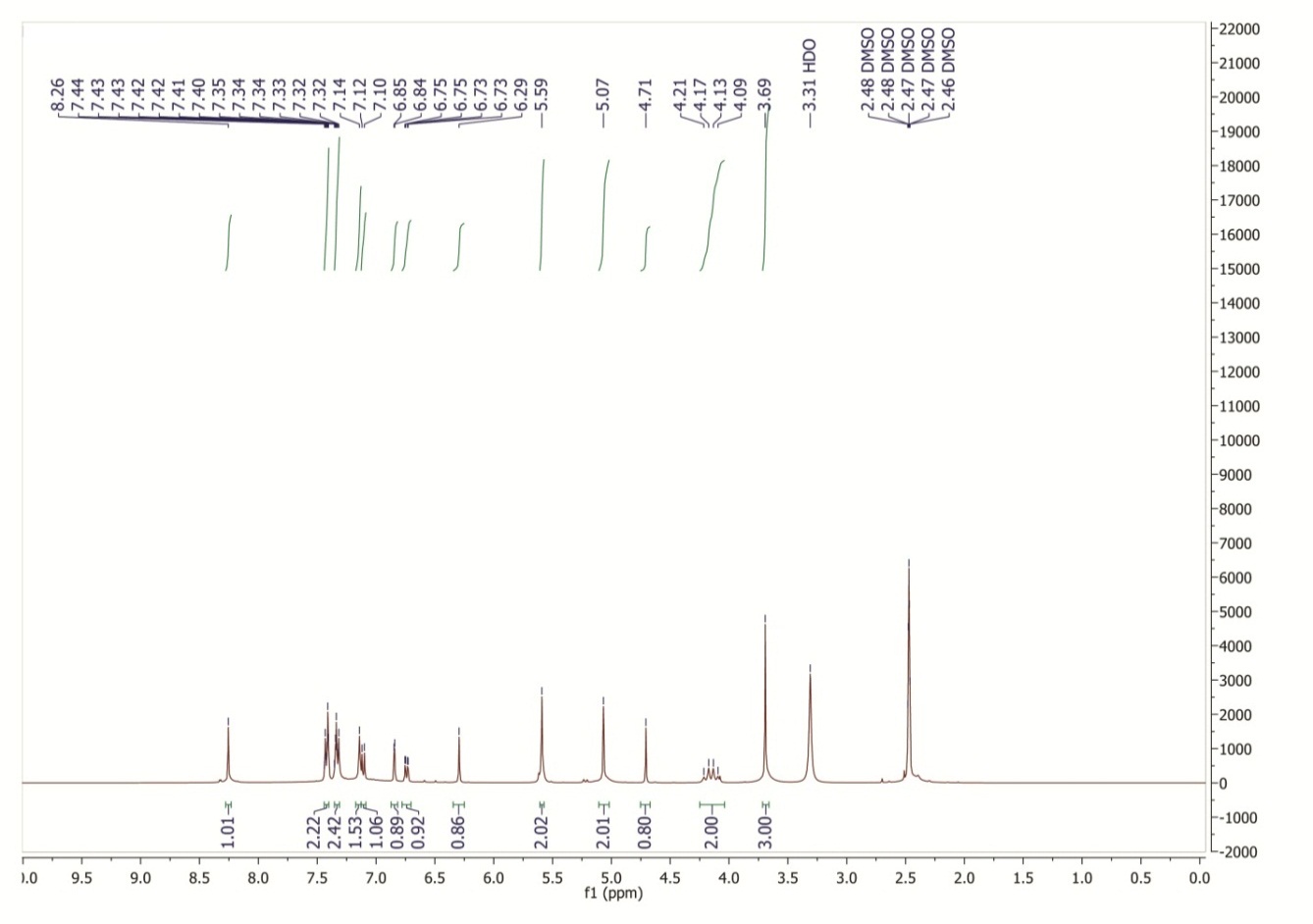

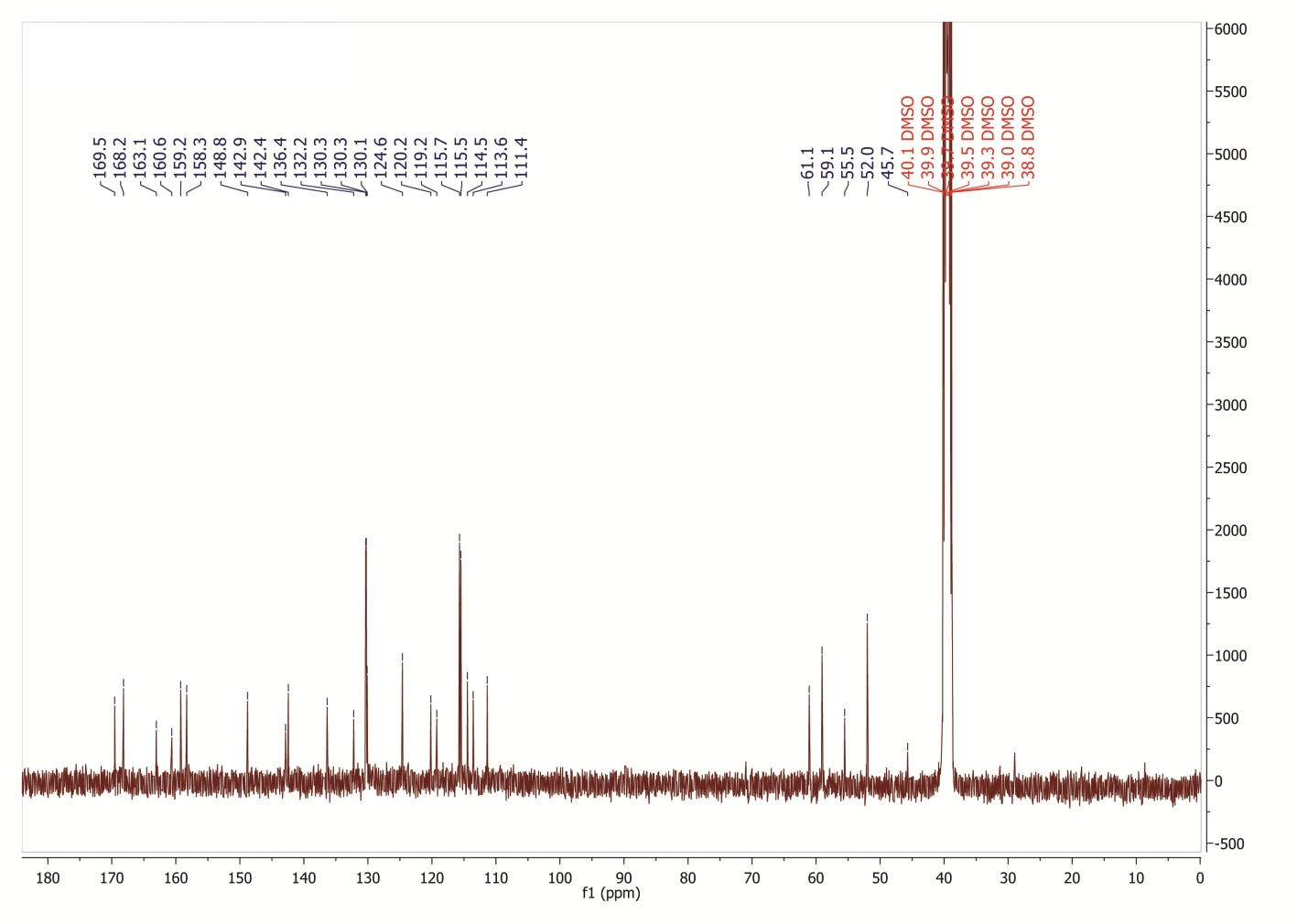

Supplement: Supplementary file 1 — Supplementary Information. [file 41598_2022_24089_MOESM1_ESM.docx]
